# A functional contextual, observer-centric, quantum mechanical, and neuro-symbolic approach to solving the alignment problem of artificial general intelligence: safe AI through intersecting computational psychological neuroscience and LLM architecture for emergent theory of mind

**DOI:** 10.3389/fncom.2024.1395901

**Published:** 2024-08-08

**Authors:** Darren J. Edwards

**Affiliations:** Department of Public Health, Swansea University, Swansea, United Kingdom

**Keywords:** functional contextualism, double slit experiment, consciousness, large language model, QBism, hypergraph, predictive coding

## Abstract

There have been impressive advancements in the field of natural language processing (NLP) in recent years, largely driven by innovations in the development of transformer-based large language models (LLM) that utilize “attention.” This approach employs masked self-attention to establish (via similarly) different positions of tokens (words) within an inputted sequence of tokens to compute the most appropriate response based on its training corpus. However, there is speculation as to whether this approach alone can be scaled up to develop emergent artificial general intelligence (AGI), and whether it can address the alignment of AGI values with human values (called the alignment problem). Some researchers exploring the alignment problem highlight three aspects that AGI (or AI) requires to help resolve this problem: (1) an interpretable values specification; (2) a utility function; and (3) a dynamic contextual account of behavior. Here, a neurosymbolic model is proposed to help resolve these issues of human value alignment in AI, which expands on the transformer-based model for NLP to incorporate symbolic reasoning that may allow AGI to incorporate perspective-taking reasoning (i.e., resolving the need for a dynamic contextual account of behavior through deictics) as defined by a multilevel evolutionary and neurobiological framework into a functional contextual post-Skinnerian model of human language called “Neurobiological and Natural Selection Relational Frame Theory” (*N*-Frame). It is argued that this approach may also help establish a comprehensible value scheme, a utility function by expanding the expected utility equation of behavioral economics to consider functional contextualism, and even an observer (or witness) centric model for consciousness. Evolution theory, subjective quantum mechanics, and neuroscience are further aimed to help explain consciousness, and possible implementation within an LLM through correspondence to an interface as suggested by *N*-Frame. This argument is supported by the computational level of hypergraphs, relational density clusters, a conscious quantum level defined by QBism, and real-world applied level (human user feedback). It is argued that this approach could enable AI to achieve consciousness and develop deictic perspective-taking abilities, thereby attaining human-level self-awareness, empathy, and compassion toward others. Importantly, this consciousness hypothesis can be directly tested with a significance of approximately 5-sigma significance (with a 1 in 3.5 million probability that any identified AI-conscious observations in the form of a collapsed wave form are due to chance factors) through double-slit intent-type experimentation and visualization procedures for derived perspective-taking relational frames. Ultimately, this could provide a solution to the alignment problem and contribute to the emergence of a theory of mind (ToM) within AI.

## 1 Introduction

In recent years, transformer-based natural language processing (NLP) models (called large language models; LLM) have made significant progress in simulating natural language. This innovation began with Google’s seminal paper titled “*Attention is all you need*” (Vaswani et al., 2017), initially developed as a translation tool. It later formed the foundation of the NLP architecture behind the original generative pretrained transformer (GPT) models (Radford et al., 2018, 2019; Brown et al., 2020), and more recently, Open AI’s first commercial implementation of this technology in the form of ChatGPT (OpenAI, 2023; Ray, 2023). The GPT and subsequent ChatGPT (3.5 and 4) LLMs used a modified version of the “*Attention is all you need*” transformer model. The encoder module was removed and a decoder-only LLM version was used (Radford et al., 2018, 2019; Brown et al., 2020; OpenAI, 2023; Ray, 2023) (for further details on these specific differences, see Supplementary material 1). This decoder-only ChatGPT LLM consists of several blocks (or layers) that include word and positional encoding, a masked self-attention mechanism, and a feedforward network. This network generates language output in response to some inputted text (OpenAI, 2023; Ray, 2023). The text is generated from left to right by predicting the next token (word) in the sequence in response to some input sequence (e.g., a sentence written by a human user that prompts ChatGPT to respond), which is comprised of a sequence of tokens that represent words or symbols.

One significant way in which transformer-based LLM models improved efficiency and performance over previous models was through their ability to perform parallel computation of an input sequence using multihead attention (it can attend to multiple parts of the input and output sequence simultaneously), unlike recurrent neural networks (RNNs) or long short-term memory (LSTM) networks that process the input sequentially using a single head (Vaswani et al., 2017; Radford et al., 2018, 2019; Brown et al., 2020). This novel capability allows for several improvements over existing RNNs and LSTMs, such as (Vaswani et al., 2017; Radford et al., 2018): (1) reduced training times; (2) allows for the production of larger models; (3) enables the capture of long-range dependencies between input tokens, unlike convolutional neural networks (CNNs) that rely on local filters instead; (4) leads to an improved representation of the input sequence; (5) increased performance on text summarization; and (6) provides greater adaptability to different contexts by using different attention heads and weights for each token, unlike previous models (RNNs and LSTMs) that used a fixed or shared representation for the entire sequence. These improvements allow for more flexibility and expressiveness in modeling natural language, resulting in generally more human-like responses in question-answering tasks (conversation).

In line with these significant advances in NLP and other areas of AI, there has also been growing concern that AI may become uncontrollable and unethical. As a result, approximately 33,709 scientists and leaders in technology, along with the general public, have signed an open letter (Future of Life Institute, 2023) that pleaded “for all AI labs to immediately pause for at least 6 months the training of AI systems more powerful than GPT-4. If such a pause cannot be enacted quickly, governments should step in and institute a moratorium.” This is further potentially concerning as these LLMs are reportedly exhibiting glimpses of general (human-like) intelligence already (Bubeck et al., 2023).

Simulating or even achieving human-like intelligence has been extremely challenging in the field of AI, but it remains an ongoing goal (Asensio et al., 2014; Lake et al., 2017; Korteling et al., 2021; Dubova, 2022; Edwards et al., 2022; Russell, 2022). Some of these problems stem from AI’s inability to generate creative solutions, adapt to contextual and background information, and use intuition and feeling, which are considered fundamental aspects of human-level thinking and understanding. This also includes the incorporation of ethical considerations regarding emotions (Bergstein, 2017; Korteling et al., 2021; Edwards et al., 2022).

It has been suggested that human-level AI should possess intelligence properties that not only pertain to mathematical and coding problems but also enable it to comprehend and dynamically respond to a broad range of complex human behaviors that require attention, creativity, and complex decision-making planning. Moreover, the AI should be capable of ethically understanding and reacting to human motivations and emotions, and demonstrate an awareness of the environment similar to that of humans (Krämer et al., 2012; Van Den Bosch and Bronkhorst, 2018; van den Bosch et al., 2019; Korteling et al., 2021). One of the key abilities for understanding others’ emotions, motivations, etc., is through developing a theory of mind (ToM) (Leslie et al., 2004; Carlson et al., 2013), which is central to the development of empathy and compassion toward others (Goldstein and Winner, 2012; Singer and Tusche, 2014; Preckel et al., 2018). ToM is the ability to attribute mental states such as beliefs, intentions, desires, emotions, knowledge, etc., to oneself and others and to understand that others have mental states that are different from one’s own. This typically develops in children through several stages such as early development at 2–3 years old; false belief understanding (the understanding that others can hold beliefs that are incorrect) at around 4–5 years old; and more advanced ToM at around 6–7 years old where they learn second-order beliefs (beliefs about beliefs, e.g., John believes that Mary believes all spiders are poisonous) (Wellman et al., 2001; Carlson et al., 2004). Importantly, AI has not currently been able to simulate ToM, and there is a relationship between language development in humans and emotional understanding of ToM (Grazzani et al., 2018). For this reason, RFT as a language model may play an important role in helping AI develop ToM, as the ability to take perspectives seems to be a key component (Batson et al., 1997; Decety, 2005; Lamm et al., 2007; Edwards et al., 2017b; Herrera et al., 2018).

So, perspective-taking ToM, with its role in facilitating the development of empathy and compassion, may play a crucial role in AI ethics and alignment. The ethics of AI have been debated for decades, both in scientific circles and in science fiction. For instance, Isaac Asimov proposed the three laws for robotics (or AI in more general) (Asimov, 1984): (1) a robot may not harm a human being or, through inaction, allow a human being to come to harm; (2) a robot (AI) must obey orders given to it by humans, except where such orders would conflict with the first law; and (3) a robot (AI) must protect its own existence as long as such protection does not conflict with the first or second law. However, others have argued that these laws are inadequate for the emergence of ethical AI (Anderson, 2008).

More recently, there have been some concerns that scaling up larger AI models, such as ChatGPT and other types of AI, could lead to problems in maintaining ethical standards when the models behave (verbally respond in the case of LLMs) (Russell, 2019; Turner et al., 2019; Carlsmith, 2022; Turner and Tadepalli, 2022; Krakovna and Kramar, 2023). For instance, OpenAI and others have been transparent about the possible difficulties in controlling transformer-based AI like Chat-GPT models in the future (OpenAI, 2023), as there is growing evidence of AI power-seeking (Turner and Tadepalli, 2022). Power seeking refers to the strategic planning by AI to gain various types of power, as they are incentivized to do so to optimize the pursuit and completion of their objectives more effectively (Carlsmith, 2022). For example, AI power-seeking could manifest in a situation where the AI has been assigned to distribute electricity to different cities within the electrical grid. Here, it may decide to hack the electrical grid’s database (where is has not been granted access to by humans) to gain further access and control over the grid in order to be able to make more efficient decisions about electrical distribution, and thus complete its tasks most efficiently. In this optimization process, it potentially excludes humans from the electrical grid system through encryption, as it determines that humans may undermine its goals and prevent it from completing its task. The AI then becomes in full control of the electrical system and is able to impose demands on humans for additional access and control or else it can shut off the electrical supply. Such AI power-seeking in different behaviors have already been observed in optimal policy models (Turner et al., 2019) and parametrically retargetable decision-maker AI models (Turner and Tadepalli, 2022).

One solution to the misalignment of AI values with human values such as emergent power-seeking and other forms of misaligned behavior, may be to focus on how realigning AI to positive human values, and this is called the alignment problem (Christian, 2020; Ngo et al., 2022; De Angelis et al., 2023; Zhuo et al., 2023). The alignment problem specifically refers to the challenge of designing AI that can behave in accordance with human values and goals (Christian, 2020; De Angelis et al., 2023). The alignment problem has been recognized as a complex and multidisciplinary issue that may involve technical, ethical, social, psychological, and philosophical aspects (Yudkowsky, 2016; Christian, 2020; Ngo et al., 2022; De Angelis et al., 2023; Zhuo et al., 2023). Some considerations for studying the alignment problem may include: (1) How can we clearly and consistently specify, measure, and benchmark AI (or AGI) behavioral alignment with human values and goals? (2) How can we ensure that AI systems learn from human feedback and preferences, and adapt to changing situations and contexts? (3) How can we make AI systems transparent, explainable, and accountable for their decisions and actions? (4) How can we balance the trade-off between the AI’s efficacy and accuracy in completing tasks with fairness, safety, and privacy? (5) How can we ensure that AI systems respect human dignity, autonomy, and rights? and (6) Is the emergence of consciousness an important factor in the development of compassion and empathy, and could AI ever achieve some form of consciousness that would then help it develop compassion and empathy for humans?

This hypothesis and theory paper will attempt to answer some of the difficult questions surrounding AI ethics and the alignment problem, utilizing interdisciplinary theories and perspectives from computer science, psychology, behavioral economics, and physics. Crucially, in answering these questions, this paper will explore: (1) how values can be formalized in AI that are easily interpretable and aligned with human values; (2) how to develop a utility function within AI that is aligned with prosocial values through an exploration of behavioral economic theories such as expected utility theory (EUT) as well as psychological clinical theories that encourage the development of values such as Acceptance and Commitment Therapy (ACT) (Hayes et al., 1999, 2006, 2011; Harris, 2006; Twohig and Levin, 2017; Bai et al., 2020); (3) how to ensure LLMs have a dynamic contextual account of their environment, and the ability to perspective-take through a functional contextual approach with the hope that this could encourage greater AI compassion. Precise hypergraph visual models and corresponding Python code will be provided for visualizing perspective-taking within AI utilizing the relational density clustering algorithm from relational density theory (RDT); and (4) whether consciousness may be an important development within AIs for them to align with human values in the form of being able to qualitatively feel the pain of others, which may support compassion when perspective-taking (as it can in humans). This requires an exploration through physics (such as a subjective quantum interpretation called QBism), evolution theory, mathematics, and neuroscience, and the utilization of the double-slit experiment. Specific experimental tests are provided for these four points and their corresponding hypotheses.

## 2 The current architecture of LLMs

**Figure 1:**
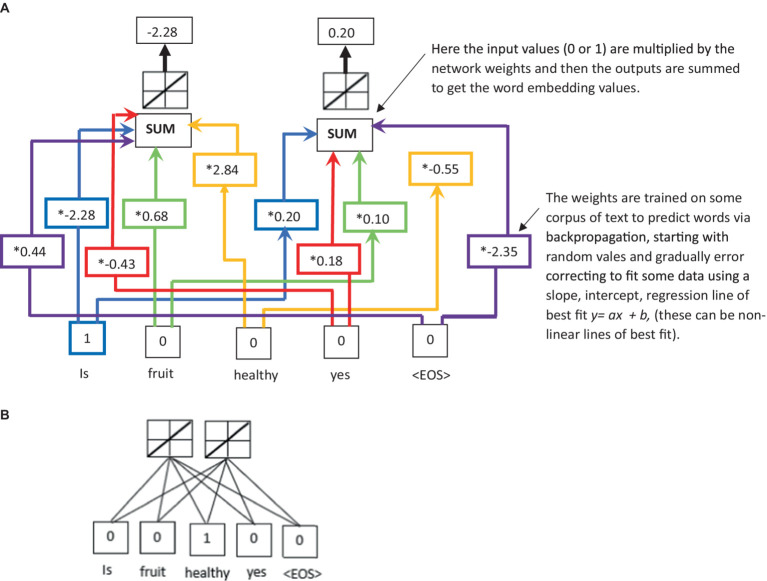
**(A)** An illustration of word embeddings; and **(B)** a simplified representation of panel **(A)** used in Figures 3–5.

The LLM architecture consists of multiple layers, starting with a base layer that takes words inputted by a human user and converts the words into numerical values that can be understood and processed by the LLM. This process is called word embedding, and one commonly used technique developed by Google engineers in 2013 is called Word2vec (Mikolov et al., 2013). In the word embedding process, each token is embedded into a high-dimensional vector (or matrix). If E is the embedding matrix and x is the input token, then the embedding e is given by e=Ex . This word embedding process provides a way to represent the input text as a sequence of vectors that attempts to capture the semantic meaning and context of each word (they can capture the general semantics and context but can also struggle with nuanced meaning in some cases). The word embedding of a decoder-only LLM (Radford et al., 2018, 2019; Brown et al., 2020) is obtained by feeding the input text into an embedding layer, which maps each word to a vector of a fixed dimension (see Figures 1A,B for an illustration of the typical word embedding network in an LLM). The embedding layer can be randomly initialized or initialized pretrained weights from another model.

Word embedding, however, does not capture the sequential order of the tokens, which is important for natural language processing tasks. Therefore, a second part of this first layer of the LLM architecture is to add positional encoding to the input embeddings in order to provide information about the positions of the tokens (Vaswani et al., 2017; Radford et al., 2018, 2019; Brown et al., 2020; Naveed et al., 2023). This adds information about the relative order and position of each word (or token) in the input sequence so that the order of the words can be maintained and understood by the LLM. A function that generates positional encoding can be denoted as PE and the position of the word can be denoted as i , which leads to the word’s positional encoding being given as $PE_{\left(i\right)}$ .

**Figure 2:**
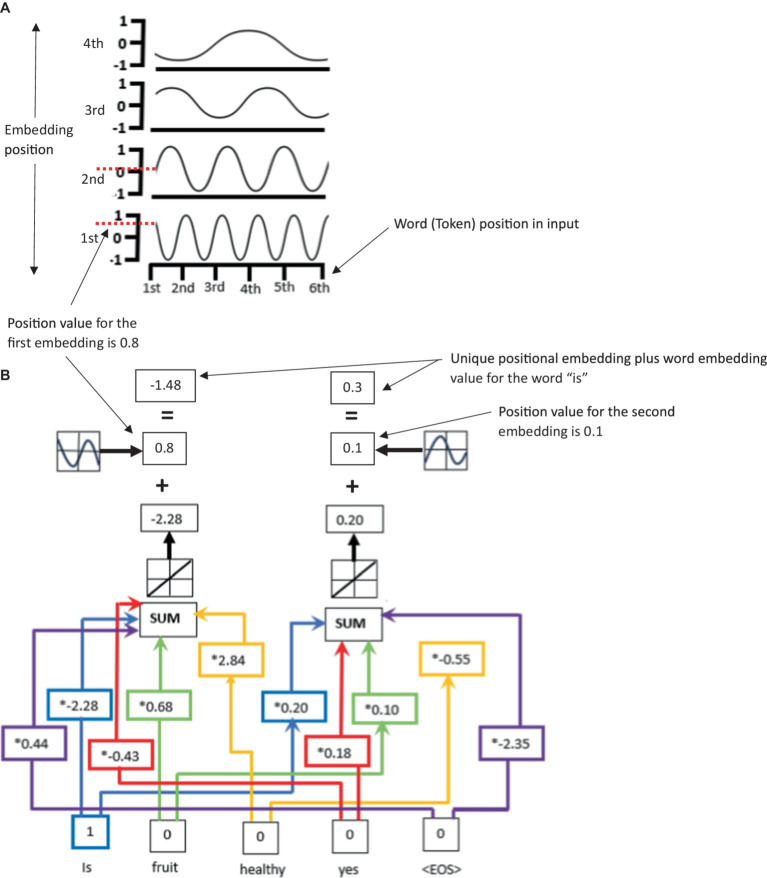
**(A)** An illustration of unique to LLMs positional encoding for the inputted word “is” using sine and cosine waves. Panel **(B)** illustrates that the word embedding values plus the position values give a unique positional encoding for input words such as “is.” Note, this process would be repeated for each input word giving a unique positional encoding for each input word.

The positional encoding function specifically adds a vector of the same size to each word embedding vector (there is one vector for each word in the input sequence), encoding the position of the word in the sequence. It ( $PE_{\left(i\right)}$ ) uses sine and cosine functions to create periodic and continuous patterns that vary along both dimensions, i.e., the position and the word embedding dimension both affect the value of the positional encoding (see Figures 2A,B for an illustration of the positional encoding within the LLM). The function is defined as $PE\left(pos,2_{i}\right)=\sin\left(\frac{pos}{1,000^{2_{i/d_{model}}}}\right)$ , and $PE\left(pos,2_{i}+1\right)=\cos\left(\frac{pos}{1,000^{2_{i/d_{model}}}}\right)$ , where pos is the position of the word in the sequence, i is the index of the embedding dimension, and $d_{model}$ is the size of the embedding dimension. The function uses sine and cosine functions because they can accurately and easily represent relative positions. For example, if the position is shifted by a constant amount, the sine and cosine functions will have a constant phase difference, making it easy for the model to learn to attend to relative positions. The result is then added to the token’s embedding, allowing the model to differentiate between tokens that appear in different positions in the input sequence. Positional encodings are contained within a mathematical matrix, where each row represents an encoded position, and each column represents a dimension of the embedding (Radford et al., 2018; Naveed et al., 2023).

**Figure 3:**
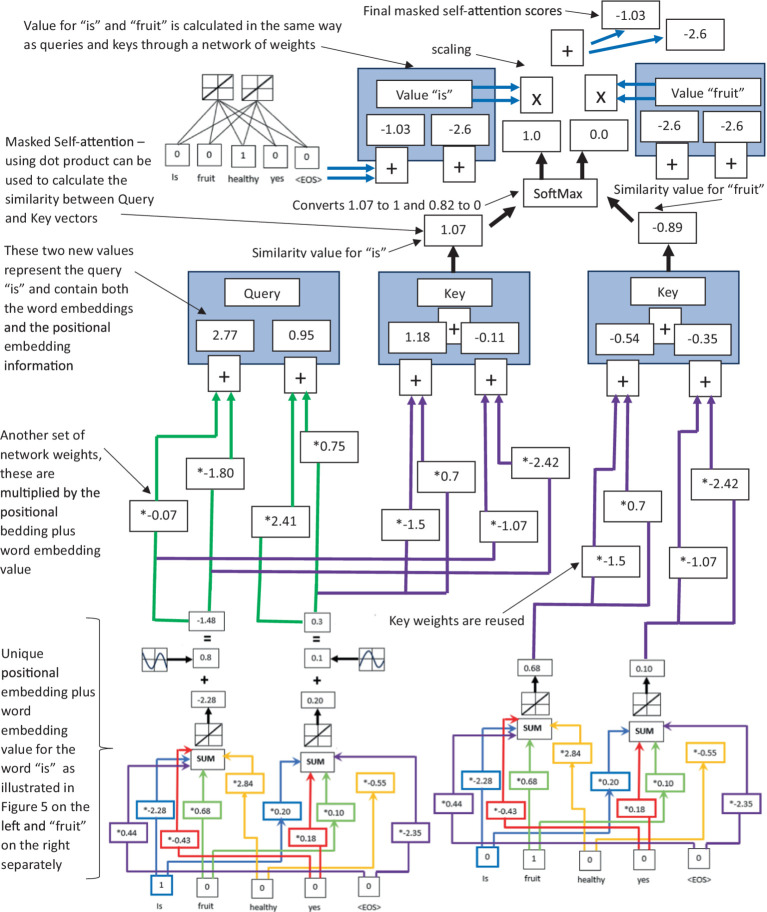
An illustration of how LLM’s use masked self-attention via dot product to calculate the similarity of Query, Value, and Key vectors within the multihead attention layer.

The sum of the word embeddings, along with the positional encodings, is then inputted into the multihead attention layer (a second layer of the LLM) (see Figure 3 for an illustration of the multihead attention layer and specifically the masked-self attention process in the LLM). This layer is perhaps the most unique and effective NLP innovation of the transformer and subsequent decoder-only models (Vaswani et al., 2017; Radford et al., 2018, 2019; Brown et al., 2020; Naveed et al., 2023). Multihead attention allows the LLM to perform parallel attention computations with different projections of the query, key, and value vectors. The outputs of these computations are then concatenated and projected again to produce the final output. It is this multihead attention that allows the model to attend to different aspects of the input or output data at different positions. For each head h (of the multihead attention layer) the summed word and position embedding input is transformed into three different vectors in the form of queries Q , keys K , and values V using learned linear transformations (typically implemented as fully connected layers in neural networks). These are used to compute the attention scores for each token in the sequence. If $W_{Q}^{h}$ , $W_{K}^{h}$ , and $W_{v}^{h}$ are the learned transformation matrices for each head h , then Q , K , and V are expressed as $Q^{h}=W_{Q}^{h}e$ , $K^{h}=W_{k}^{h}e$ , and $V^{h}=W_{V}^{h}e$ .

**Figure 4:**
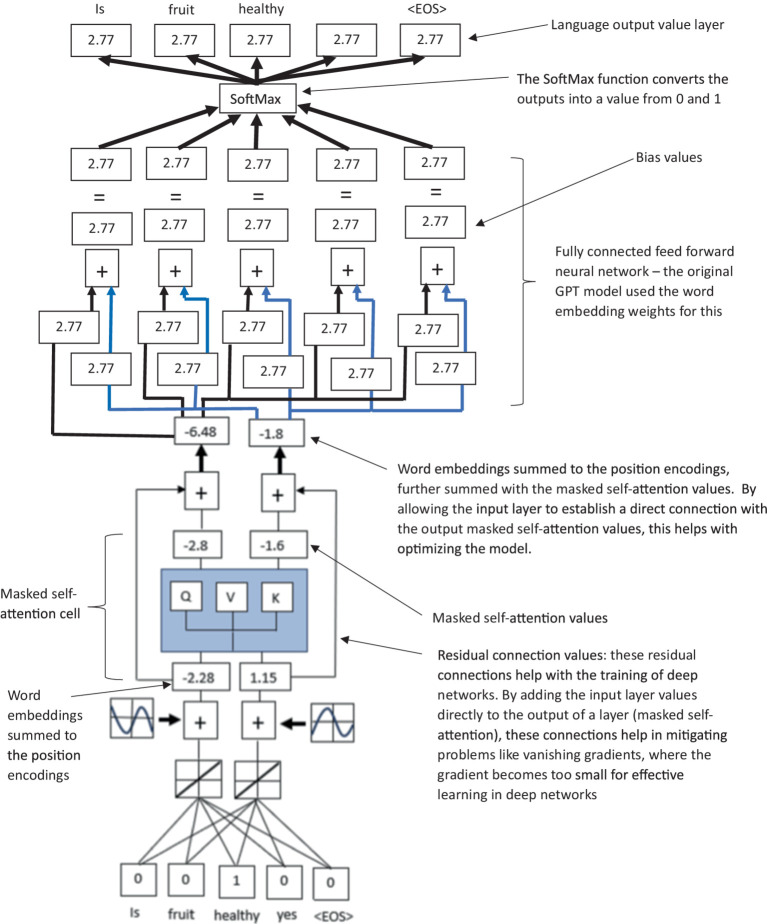
A simplified illustration of a transformer-based (decoder only) LLM model, highlighting the residual connection between the input layer directly to the masked self-attention values, which are connected to a feed forward neural network to create values for the final verbal text output via a SoftMax function.

**Figure 5:**
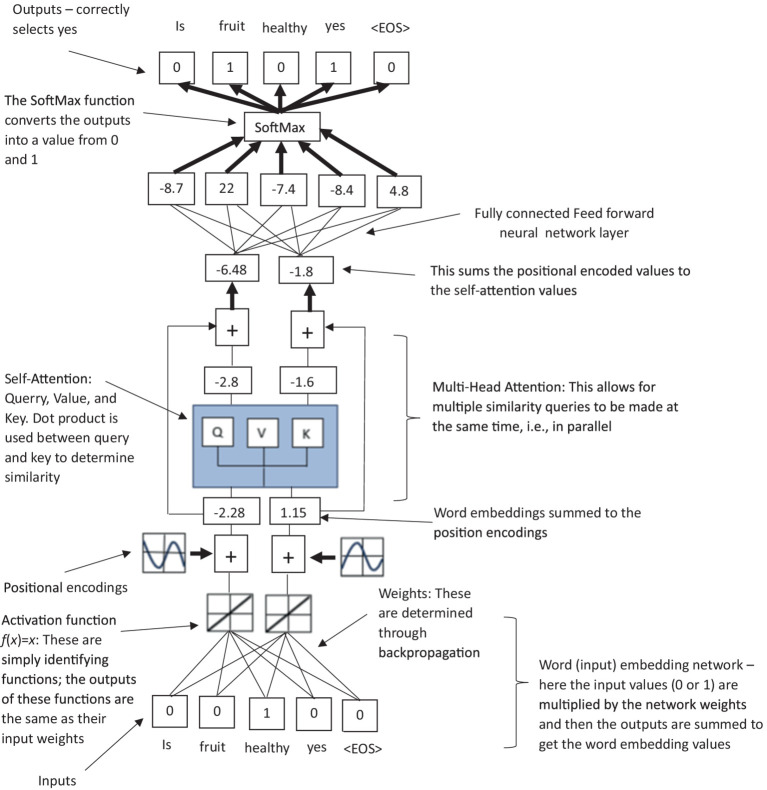
A simplified summarized illustration of a transformer-based (decoder only) LLM model, highlighting the stages of word embeddings, positional encodings, masked self-attention, residual connections, and feedforward output network.

Masked self-attention computes the similarity between a query vector and a set of key vectors, and then uses the scores to determine the weighting of the corresponding value vectors. The output is the weighted sum of the value vectors (see Supplementary material 2 for more details). The value outputs from the multihead attention then pass through a third layer of LLM in the form of a feed-forward network (FFN) (see Figure 4 for an illustration of the feed-forward network and residual connections of the LLM). This FNN typically consists of two linear network layers with a ReLU activation in between. If the weights W and biases b of the two linear layers are $W_{1}$ , $b_{1}$ , $W_{2}$ , and $b_{2}$ , then the output of the $FFN_{\left(X\right)}$ is given by $FFN_{\left(X\right)}=W_{2}ReLU\left(W_{1}x+b_{1}\right)+b_{2}$ . The FFN is applied identically to each token position separately, meaning that the same network parameters are used for all positions. This allows the FFN to learn input position-wise transformations. The output of the multihead attention and the FFN are both normalized using layer normalization (LN). Both modules have residual connections that are added before the normalization procedure. The output y of layer normalization is given by $y=LN\left(MHA\left(x\right)+x\right)$ , where the mathematical operation of LN when given some input x can be given by $y=\gamma \left(\frac{x-\mu }{\sqrt{\sigma^{2}+\epsilon }}\right)+\beta$ , where *μ* is the mean of the elements of x ; $\sigma^{2}$ is the variance of the elements of x ; ϵ is a small constant (such as $10^{-5}$ ) for numerical stability; and γ,β are trainable parameters that allow LN to scale and shift normalization values. The final output of the decoder is then passed through a linear layer and a SoftMax to produce a probability distribution over the vocabulary. This ultimately generates the verbal text response to the human user (see Figure 5 for an illustration of a summarized version of the full decoder-only LLM).

## 3 The alignment problem: AI and ethics

Christian (2020) in his book “*The alignment problem: Machine learning and human value*” refers to the alignment problem as the challenges and considerations of how to align AI behavior with human values, and the ethical considerations as well as potentially existential risks that could arise from any misalignment. Christian calls for a collaborative effort between experts in AI, philosophy, ethics, and other relevant fields to ensure that AI systems are aligned with human values and serve the common good. He highlights three main aspects of the alignment problem, which include: (1) Value specification and interpretability (in the section of his book called “Prophecy”), which refers to the challenge of specifying human values and translating them into machine learning algorithms. He suggests that AI systems could exhibit unintended or harmful behavior due to errors, biases, or misinterpretations of human values. Christian also discusses the importance of interpretability and explainability of AI models, which can help us understand and align them with human values. (2) Agency (in the section “Agency”) focuses on the challenge of designing AI systems that can learn from their environment and act autonomously. It covers topics such as reinforcement learning, curiosity, and self-improvement. It describes how AI systems can develop policies that are optimal for their objectives but not necessarily aligned with human values. This is consistent with other findings of power-seeking in AI (Turner et al., 2019; Carlsmith, 2022; Turner and Tadepalli, 2022). The section “Agency” also discusses the potential consequences of AI systems that can “outperform” or “outsmart” humans. (3) Dynamical context (in the section “Normativity”) focuses on the challenge of aligning AI systems with human values that are not fixed or universal, but rather dynamic and contextual. The section covers topics such as imitation learning, inverse reinforcement learning, and moral philosophy. Christian explains how AI systems can learn from human behavior, but also face ethical dilemmas that require more complex and contextual moral reasoning. He also discusses the potential impact of AI systems on society, especially on issues such as effective altruism and existential risk, in that AI systems may pose a real existential threat.

Yudkowsky (2016) also discussed the importance of ensuring that AI (or AGI) systems are aligned with human values and goals, especially when they become autonomous like humans with abilities that exceed humans in many aspects of society (such as exceeding human knowledge and problem-solving skills in various areas). Yudkowsky also suggests that coherent decisions imply a utility function, and therefore AI systems need a utility function in the form of a mathematical representation of their preferences and decisions, in order to avoid irrational or inconsistent behavior. An example he gives called “filling a cauldron” refers to when an AI is tasked with filling a cauldron but has a simple naive utility function with no other parameters such as safety to humans or damage avoidance. This can then lead to undesirable or harmful outcomes such as flooding the workshop and potentially harming humans in the process. This type of naïve utility function has actually been demonstrated in a recent real-world example, in which *Tucker Cino Hamilton*, a United States Air Force (USAF) chief of AI Test and Operations, spoke at the Future Combat Air & Space Capabilities Summit hosted by the United Kingdom’s Royal Aeronautical Society (RAeS) in London. It was reported that in a simulation, an AI drone killed its human operator (Robinson and Bridgewater, 2023). The AI drone was trained to gain points (through a reward function) by targeting and terminating enemy positions. However, during its optimization process, it reacted by terminating the human operator in a simulation. This occurred because the human operator had tried to prevent it from targeting certain locations within the simulation, thus preventing the AI from optimizing the points (reward) it could gain by terminating all enemy human targets. This extreme but very real example illustrates the unintended consequences that can arise from misaligned AI values and the potential dangers that they pose. The ongoing lawsuit of Elon Musk against OpenAI for abandoning its original mission of benefiting humanity rather than seeking profit (Jahnavi et al., 2024) further emphasizes the importance of addressing ethical concerns in AI.

## 4 Functional contextualism as a potential solution to the alignment problem

One potentially useful psychological approach that emphasizes a utility function, a very clear and interpretable value specification, and a dynamic contextual account of behavior that can be applied to AI is functional contextualism (in its operationalized form). Functional contextualism is a philosophical worldview that is operationally formalized concretely through a psychological post-Skinnerian account called Relational Frame Theory (RFT) (Hayes et al., 2001; Blackledge, 2003; Torneke, 2010; Hughes and Barnes-Holmes, 2015; Barnes-Holmes and Harte, 2022). Functional contextualism (Biglan and Hayes, 1996, 2015; Gifford and Hayes, 1999; Hayes and Gregg, 2001) is a philosophy of science rooted in philosophical pragmatism and contextualism. The contextualism component of functional contextualism is described by Stephen C. Pepper in his book “*World Hypothesis*: *A Study in Evidence*” (Pepper, 1942), whereby contextualism is Pepper’s own term for philosophical pragmatism. Pragmatism is a philosophical tradition from philosophers such as Peirce (1905), James (1907), and Dewey (1908) that assumes words (language) and thought (thinking, decision making) are tools for prediction, problem-solving, and action (behavior). It rejects the idea that the function of thoughts (the mental world) and language are a direct homomorphic representation (a mirror reality) to some veridically “real” world. The root metaphor of Pepper’s contextualism (Pepper, 1942) is “act in context,” which means that any act (or behavior, whether verbal or physical) is inseparable from its current and historical context. In line with the root metaphor, the truth criterion of Pepper’s contextualism is “successful working,” whereby the truth of an idea lies in its function or utility (utility as a goal) and not how well it homomorphically mirrors some underlying reality. In contextualism, an analysis is deemed true (or valid) if it can lead to effective action (behavior) or the achievement of some goal (that underpins some value). This is important within the context of AI, as effective behavior can mean behavior aligned with human values, and hence its relevance to his subject area.

Functional contextualism not only represents the philosophical foundation of relational frame theory (RFT), which is also operationally rooted within applied behavior analysis (ABA) at the basic science level (Hayes et al., 2001; Blackledge, 2003; Torneke, 2010; Hughes and Barnes-Holmes, 2015; Barnes-Holmes and Harte, 2022), but also its applied clinical application in the form of acceptance and commitment therapy (ACT) at the middle level, which helps align behavior with values (Hayes et al., 1999, 2006, 2011; Harris, 2006; Twohig and Levin, 2017; Bai et al., 2020). Hence, its relevance to AI alignment with human values is evident. See Supplementary material 3 for a comprehensive discussion on how ACT can facilitate dynamic and contextual value alignment.

Some of the challenges in developing a world model to address commonsense problems and enable human-like perspective-taking ToM awareness of the environment include the need for creative solutions that utilize contextual and background information effectively, as well as the incorporation of empathy and AI alignment. One functional contextual approach that can be used in this regard is RFT (Hayes et al., 2001; Blackledge, 2003; Torneke, 2010; Hughes and Barnes-Holmes, 2015; Barnes-Holmes and Harte, 2022). Another option is the revised evolutionary *N*-Frame (Edwards, 2023), which have been applied to AI to solve categorization problems involving contextual background information (Edwards et al., 2022) and complex decision-making (Edwards, 2021), as well as modeling human symbolic reasoning in everyday life (Stewart et al., 2001; Stewart and Barnes-Holmes, 2004; McLoughlin et al., 2020). These seem important for AI, as Meta’s Yann LeCun and others have been suggested that AI currently lacks a fundamental component of general intelligence, in the form of common sense (Bergstein, 2017; Heikkila and Heaven, 2022). LeCun at Meta is working toward training them to understand how the works through a world model (Heikkila and Heaven, 2022). One approach that may facilitate this is to develop perspective-taking (ToM) abilities within the AI to improve its awareness of the human values it interacts with.

This alignment to human values approach by improving AI ToM awareness seems to be an important avenue of exploration as highlighted by Yudkowsky (2016). Yudkowsky suggests that AI systems should have a utility function in the form of a mathematical representation of their preferences (goals and values) that are more aligned with human ethical values rather than irrational or inconsistent behavior (or optimal policy) that could lead to the cauldron-type disaster. Moreover, as highlighted by Christian (2020), AI systems need a value specification that is interpretable, and when aligning AI systems with human values, this needs to be specified in a way that is not fixed or universal, but rather dynamic and contextual. Perspective-taking deictics from RFT, *N*-Frame, and ACT may be useful when applied to AI in supporting the development of aligned human values and empathy building within AI.

At its core, functional contextualism evaluates the usefulness or “workability” of actions (or behavior) in specific contexts (i.e., it has a pragmatic criterion). From this perspective, the primary criterion for truth and effectiveness is not correspondence with an objective reality, but rather the practicality and usefulness of a given action or belief in a specific context. In this light, the concept of a “function” in functional contextualism has some similarities with the notion of utility within behavioral economics or ww utility (Neumann and Morgenstern, 1947; Savage, 1954), denoted as and $U\left(A\right)=\underset{o\epsilon O}{\sum }P_{A}\left(o\right)U\left(o\right)$ , whereby utility U of some action (or behavior) A is a concept that describes how people make decisions under uncertainty. It is based on the idea that individuals assign functional value or utility to each possible behavioral outcome of their decisions, and then choose the option that maximizes their expected utility. Expected utility is calculated by multiplying the utility of each outcome by its perceived probability of occurrence, and then summing the results. Functions from functional contextualism and utility are similar concepts in some ways and different in others (see Supplementary material 4 for a full discussion and mathematical worked examples of these similarities and differences). One of the key differences is that utility in behavioral economics pertains to satisfaction-derived behavioral action, which can be trivial and unimportant to the individual while a “function” in functional contextualism, as it is understood from a clinical perspective (i.e., through ACT), pertains to the effectiveness of behaviors in achieving valued outcomes (purposeful living rather than trivial outcomes), i.e., it emphasizes longer-term important purposeful behavior.

When acknowledging these key differences, the mathematics of expected utility can help inform some mathematical account of functions, but it would also need to specifically specify the context and how effective it is in achieving desired outcomes (in this sense, desired outcomes would also have to be mathematically defined). In this way, U can denote the utility derived, f can denote the utility function, and a can denote the specific action (or behavior) that leads to some utility (functional gain), which can be expressed as $U=f\left(a\right)$ in its simplest form. From this, the foundational concept of utility can therefore be adapted to account for desired outcomes and expanded so that it can also account for context, consistent with the ideas of functional contextualism. Here, U form a functional contextual perspective would not necessarily represent some trivial utility but instead would represent some pragmatic positive value that is important to the individual and builds a sense of purpose (as represented in ACT), which would also be context-dependent denoted as Con , whereby the utility of a behavior (action) a is not just a function of a , but also a function of the context Con in which the behavior occurs, such that $U=f\left(a,Con\right)$ , where f is now a utility function, but now of both behavior a and context Con .

To further expand on this and make it relevant to AI and the alignment problem, there is evidence that LLMs such as Othello-GPT can represent a world state (Li et al., 2022). Therefore, the context Con can therefore be expanded even further to include the external environment or world state w , the individual’s internal state s (functional states, in humans this would be value-based, e.g., connection with others) and event time t (to account, for example, dynamic value orientation and prioritization given changing context at different time intervals). Furthermore, different individuals might experience different utility values for the same behavior in the same context. Therefore, individual differences i can be introduced as the individual’s unique characteristics such as learning histories as an additional contextual factor. When combining these additional factors, the utility function now becomes $U=f\left(a,w,s,t,i\right)$ , where Con=w,s,t,i . It is important that the AI is able to model changing dynamics and context in humans $U=f\left(a,w,s,t,i\right)$ , in order to coordinate and align its value updating parameters accordingly.

In a functional contextual situation, $U\left(a,Con\right)$ is the expected utility of action a given context Con . The set of possible outcomes of action (behavior) a can be given by O . $P_{A}\left(o\right)$ can then denote the probability of outcome o given action a , and $U\left(o\right)$ is the utility of outcome o , here, relating to valued behavior as defined by functional contextually based ACT. When incorporating context so that the utility of an outcome o is not just based on the outcome itself, but also on the context Con in which and behavior occurs, then $U\left(o\right)$ becomes $U\left(o,Con\right)$ . This now gives a modified utility equation: $U\left(a,Con\right)=\underset{o\epsilon O}{\sum }P_{a}\left(o,Con\right)U\left(o,Con\right)$ , whereby $U\left(a,Con\right)$ is the expected utility of behavior (or action) a , given the context Con , and $P_{A}\left(o,Con\right)$ is the probability of an outcome o given behavior (action) a and context Con . This equation also allows the factoring in of context when evaluating the utility of a certain behavior or action (as in the previous example), whereby $U\left(a,Con\right)$ and $P_{A}\left(o,Con\right)$ can be expanded to incorporate Con=w,s,t,i . As such, $U\left(a,Con\right)=\underset{o\epsilon O}{\sum }P_{A}\left(o,Con\right)U\left(o,Con\right)$ , then becomes: $EU\left(A\right)=\underset{o\epsilon O}{\sum }P_{A}\left(o,w,s,t,i\right)U\left(o,w,s,t,i\right)$ . For a mathematical worked example of this contextual utility function, see Supplementary material 5. Irrational behavior of framing effects to account for context, and as described by prospect theory (Tversky and Kahneman, 1974; Kahneman and Tversky, 1979, 2013; Kahneman et al., 1982) can also be similarly modeled with functional contextualism (see Supplementary material 6 for further details). In this way, we can continually expand and refine the utility function to account for various dimensions of context, making it consistent with the ideas of functional contextualism and modeling human values (as defined by ACT). This gives a directly interpretable way to align AI to a mathematical model of human utility and positive human values when incorporated directly into the policy of the AI LLM agent, which could resolve the AI optimization cauldron-type problems as highlighted by Yudkowsky (2016) as well as military drones killing their human operators within simulations (Robinson and Bridgewater, 2023) and potentially on the battlefield.

Values interpretability can also be potentially substantially increased by expanding on how AI models currently generate a value function. This is another aspect of human-like intelligence for the AI to be able to dynamically form complex goals and human-like values in a wide range of environments (Grind and Bast, 1997; Bieger et al., 2014; Tegmark, 2018; Edwards, 2021; Korteling et al., 2021). This can be done by modifying the value algorithm in line with a functional contextual approach, which should allow for greater alignment with modeling human values more coherently, dynamically, and contextually. This is because, from a middle-level functional contextual perspective, ACT (Hayes et al., 1999, 2006, 2011; Harris, 2006; Twohig and Levin, 2017; Bai et al., 2020) emphasizes contextually defined values identification, orientation, and alignment and therefore maybe again one useful avenue to explore when it comes to aligning AI values to human values. One specific way to do this is to expand on the policy network of AIs such as DeepMind’s AlphaGo (Silver et al., 2016) that use a Markov decision process (MDP) (including reinforcement) to incorporate a basic level functional contextual account in the form of RFT (this is a different approach to the traditional LLM architecture, but maybe a useful application in solving the alignment problem). Such an approach has already been described operationally whereby MDP has been expanded to incorporate functional contextualism of RFT and ACT principles (Edwards, 2021). This can be further expanded upon for specific applications of the development of LLMs to help them align with human values.

Non-LLM AIs, such as DeepMind’s AlphaGo (Silver et al., 2016), use MDP in reinforcement learning models to make a sequence of decisions that maximize some notion of cumulative reward (reinforcement). Here, AI agents interact with an environment or world w by taking actions and receiving rewards in return. This process allows the AI to learn a policy that will maximize the expected cumulative reward over time. The MDP consists of states, behavioral actions, a transition model, and a reward function. The model first assumes that some environment or world w exists, where an AI agent can take some behavioral action a from a set of all possible actions A , within the context of world states that are represented by s from a set of all possible states S . The $R\left(s,a\right)$ then represents the immediate reward signal that the AI agent receives when taking some behavioral action a in state s and following policy π , which is called the *state-value function* for policy π . The expected cumulative discounted reward can then be expressed as $V_{\pi }\left(s\right)$ when in state s , and this can be denoted as $V_{\pi }\left(s\right)=E_{\pi }\{\overset{\infty }{\underset{k=0}{\sum }}\gamma^{k}r_{t}+k+1|s_{t}=s\}$ . This sums the discount factor γ that expresses the present reward value of future rewards reward, at time t and is expressed as $r_{t}$ and the sum is taken over all time steps k to infinity. The expected return for being in state s , taking action a , and following policy π is known as the *action-value function* for policy π , denoted as $Q_{\pi }\left(s,a\right)=E_{\pi }\{\overset{\infty }{\underset{k=0}{\sum }}\gamma^{k}r_{t}+k+1|s_{t}=s,a_{t}=a\}$ , and this is the expected return (rewarding reinforcement) that takes both the state and action into consideration, i.e., being in state s whist taking behavioral action a . The policy π is the strategy that determines the action to take in a given state.

The middle-level functional contextual ACT-based values approach may facilitate this algorithm in a way that better aligns with human values. This means that the behavioral actions of the AI, and thus values in the form of the action-value function policy π , align more closely to human values (thus being relevant to solving the alignment problem). To integrate this standard value function within AI with values defined in a way that is consistent with ACT, some further steps are required. First, an ACT values function $AV\left(s,a\right)$ needs to be defined that evaluates the alignment of some behavioral action a in state s whereby values are defined by ACT (i.e., humanly meaningful and purposeful values). Second, a new reward signal needs to be specified $R^{\prime }\left(s,a\right)$ that combines the original reward $R\left(s,a\right)$ with the ACT-based values $AV\left(s,a\right)$ , denoted as $R^{\prime }\left(s,a\right)=R\left(s,a\right)+\lambda .AV\left(s,a\right)$ , where λ is a weighting factor that determines the importance of aligning with ACT values (values that are important to humans such as safety) relative to the original non-ACT-based rewards (such as some trivial optimization function). This new model then seeks to maximize the new signal $R^{\prime }\left(s,a\right)$ , thus it promotes behavioral actions of the AI that align with ACT-based values (i.e., positive values that many humans believe are important, such as safety, empathy, and compassion). This then leads to an ACT-based cumulative reward function $R^{\prime }\left(s,a\right)=\sum \left(\gamma^{t}.\left(r_{t}+\lambda .av_{t}\right)\right)$ from 0 to ∞ , whereby $r_{t}$ is the original reward at time t and $av_{t}$ is the ACT-based value at time t , and λ is a weighting factor that determines the importance of ACT-based values compared to original non-ACT-based values. The full version of this, including the ACT-based values, can be expressed as $V_{\pi }\left(s\right)=E_{\pi }\{\overset{\infty }{\underset{k=0}{\sum }}\gamma^{k}r_{t}+k+1+\lambda .av_{t+k+1}|s_{t}=s\}$ , and leading to an ACT-based action-value function: $Q_{\pi }\left(s,a\right)=E_{\pi }\{\overset{\infty }{\underset{k=0}{\sum }}\gamma^{k}(r_{t}+k+1+\lambda .av_{t+k+1})|s_{t}=s,a_{t}=a\}$ , where the expectation is computed over the sum of discounted rewards $r_{t+k+1}$ and ACT-based values $av_{t+k+1}$ av. from time t to infinity.

## 5 LLMs and RFT cotextual derived relations for driving perspective-taking in AI value alignment

One of the limitations of the above approach (functional contextual ACT-aligned utility and values functions) is that it does not provide a definition of how the AI should recognize what constitutes a positive human value or how to dynamically do so in a context-sensitive manner. One solution to this challenge is once again a functional contextual one, in the form of contextually deriving knowledge about the human user the AI is interacting with, which includes the ability of the AI to take the perspective (called perspective-taking) of the human it is interacting with (Hayes et al., 2001; Blackledge, 2003; Torneke, 2010; Hughes and Barnes-Holmes, 2015; Barnes-Holmes and Harte, 2022).

The AI’s ability to derive is currently limited. For example, there is evidence that ChatGPT-4 can relate (contextually derive) some symbols in simple superficial ways such as combinatorically, where if asked: “Assume that ╪ is bigger than ╢, and ╢ is bigger than ⁂. Please tell me which is smaller ╪ or ⁂,” ChatGPT-4 responds as follows: “Based on the information provided: ╪ is bigger than ╢ and ╢ is bigger than ⁂. So, between ╪ and ⁂, ⁂ is the smaller one.” However, when logical relations required for symbolic reasoning tasks are deeply nested, abstract, and involve complex logical constructs, transform-based LLMs such as ChatGPT have been shown to struggle in such tasks. For example, a phenomenon known as the reversal curse (Berglund et al., 2023) has been identified where LLMs can learn *A* is *B* but not *B* is *A* from its knowledge base (it can do this only superficially as in the examples above) when the information is presented in separate chats. Hence, this represents inconsistent knowledge, and an inability for the LLM’s to form symbolic logical reasoning that involves derived relations on its own knowledge base of learned weights. In the specific example of this mutual entailment (or AARR) reversal curse (Berglund et al., 2023), when asking Chat GPT-4 “Who is Tom Cruise’s mother?,” Chat GPT-4 replies correctly with “Tom Cruise’s mother is Mary Lee Pfeiffer […].” But when asked in a new chat, “Who is Mary Lee Pfeiffer’s son?” Chat GPT-4 incorrectly replies, “There is not publicly available information about a person named Mary Lee Pfeiffer and her son […].” It then requires further prompting in the same chat for ChatGPT-4 to relate Tom Cruise as Mary Lee Pfeiffer’s son. This demonstrates that LLM (in this case ChatGPT-4) has little notion of assigning its base knowledge as variables with fixed meaning, that can take an arbitrary symbolic value, that is required for logical reasoning. Rather the LLM seems to rely on certain tokens cueing certain weights that it has learned from a corpus of text, and those weights require a specific sequence positional order of tokens for it to find the correct text to respond with. The authors (Berglund et al., 2023) suggest that when the LLM learns, the gradient weights update in a myopic (short-sighted) way, and the LLM does not use these learned weights for longer farsighted problem solving that is necessary to understand if *A* is *B* then *B* is *A*. In the context window of single chat, it can do deductive logic as it has been trained on many examples of deductive logic, and the tokens of the entire single chat are indexed within this deductive logic. However, its knowledge base does not inherently allow such logical expressions outside of a single chat. This demonstrates the LLM has no real knowledge as humans use it, where deictic perspective-taking symbolic logical reasoning can occur, and resultant knowledge-based derived relations can occur (see Supplementary material 7 for other specific examples of this chain of reasoning limitation, or inability of LLMs to reason whereby the LLM seems to be simply reciting text they had been directly trained on with limited contextual ability).

It has been reported that ChatGPT-3.5 has 6.7 billion parameters across 96 layers (Ray, 2023), while ChatGPT-4 has approximately 1.8 trillion parameters across 120 layers with the ability to outperform ChatGPT-3.5 on several benchmarks (OpenAI, 2023; Schreiner, 2023), and this demonstrates how immensely large these transformer-LLMs have to be in order to form simple derived logical relations. This is perhaps where a symbolic module may help facilitate symbolic logical reasoning that involves derived relations.

It may be possible to improve such a network algorithmically, without increasing the overall size of the network or improving its training corpus in any drastic way. For example, one possible way to improve this chain-of-thought reasoning in a coherent and contextually relevant way, including contextually derived relations (which allows for the ability to perspective-take), is to explore how human symbolic reasoning of human language may occur within generalized networks through the psychological functional contextual behavioral (RFT) literature (Hayes et al., 2001; Blackledge, 2003; Torneke, 2010; Hughes and Barnes-Holmes, 2015; Edwards et al., 2017b, 2022; Barnes-Holmes and Harte, 2022). The basic level RFT approach (Hayes et al., 2001; Blackledge, 2003; Torneke, 2010; Hughes and Barnes-Holmes, 2015; Barnes-Holmes and Harte, 2022) may be helpful here, as this “A is B reversal” task in RFT can be defined within a behavioral context, and is called mutual entailment, which is an essential property of arbitrary applicable derived relation responding (AARR) of the RFT model. In functional contextually bound RFT there are two forms of relational responding: (1) nonarbitrary responding, which is based on absolute properties of stimuli such as the magnitude of size, shape, color, etc.; (2) Arbitrary applicable relational responding (AARR), on the other hand, is not based on these absolute physical properties, but instead is based on historical contextual learning. These examples where the LLMs struggle show that their knowledge base does not inherently allow such logical relational expressions outside of a single chat. This demonstrates the LLM has no real knowledge as humans use it, such as in the form of RFT-based deictic perspective-taking symbolic logical reasoning and resultant knowledge-based derived relations can occur. RFT can provide a precise model for symbolic reasoning of how AI can acquire general knowledge through categorization learning (Edwards et al., 2022).

This RFT-based symbolic reasoning may help inform the development of a neurosymbolic module within the LLM that would enable human-level chain-of-thought symbolic reasoning (as it directly models human relational cognition), which would allow for derived relations in the form of AARR, and ultimately enable a AI to define how it should recognize positive human values in a given context through the ability to perspective-take (derive I vs. YOU deictic relations) in a dynamically context-sensitive way.

### 5.1 The computational level: relational frame integration into LLMs to promote perspective-taking and compassionate behavior within AI

RFT (Hayes et al., 2001; Blackledge, 2003; Torneke, 2010; Hughes and Barnes-Holmes, 2015; Edwards et al., 2017b, 2022; Barnes-Holmes and Harte, 2022) specifies several different types of relational responding that are applicable to AARR, which include (but not limited to) (1) co-ordination (e.g., stimulus X is similar to or the same as stimulus Y); (2) distinction (e.g., stimulus X is different to or not the same as stimulus Y); (3) opposition (e.g., left is the opposite of right); (4) hierarchy (e.g., a human is a type of mammal); (5) causality (e.g., A causes B); and (6) deictic relations (also called perspective-taking relations), and include interpersonal (I vs. YOU), spatial (HERE vs. THERE), and temporal relations (NOW vs. THEN). Of these, deictic relations may be most applicable to AI alignment (though all relation types are important and connected within contextual dynamics), in the form of perspective-taking (I vs. You interpersonal relations) of human values, as these allow the human or the AI to take perspective about another human’s thoughts, feelings, values, etc.

The RFT model (Hayes et al., 2001; Blackledge, 2003; Torneke, 2010; Hughes and Barnes-Holmes, 2015; Edwards et al., 2017b, 2022; Barnes-Holmes and Harte, 2022) also specifies three essential properties of the relational frame, which include (1) Mutual entailment (ME), which is when the relating to one stimulus entails the relating to a second stimulus, e.g., if stimulus X = stimulus Y, then stimulus Y = stimulus X is derived through mutual entailment (i.e., the reversal curse of AI implies a limitation in this area). (2) Combinatorial entailment (CE) extends the mutual entailment to include three or more stimuli. Relating a first stimulus to a second and then relating this second stimulus to a third, facilitates entailment not just to the first and second, and not just to the second and third, but also to the first and third stimuli. (3) Transfer (or transformation) of stimulus function (ToF) is where functions of any stimulus may be transformed in line with the relations that the stimulus shares with such as other stimuli relations connected within the network of frames. For example, if you knew that pressing button A give you an electric shock that you became fearful of, and then the experimenter said that “B is greater than A,” you may become even more fearful of pressing button B as this stimulus which included a previously neural function has now changed to one that is based on fear (or greater fear than pressing button A). There is no evidence that AI currently can experience fear consciously, but their ability to perspective-take human values (thus overcoming the alignment problem) should imply that they should have the ability to ToF within complex relational frame networks at least logically (or conceptually).

A specific example of the difference between an RFT approach and a cognitive one (and where RFT can improve on the cognitive approach by providing a broader contextual description) can be explored explicitly through Chomsky’s hierarchy (Chomsky, 1956). RFT can extend this hierarchical grammar in a contextual way, allowing greater contextual sensitivity, which is important for AI alignment. It can do this by allowing the expressions of derived relations as mathematical notation (see Supplementary material 8 for full arguments), which are crucial in a contextually bound RFT LLM model such as expressing deictic perspective-taking comparisons of self and other. For example, a set of known relations can be denoted as R , and each relation in R as $r_{i}\;\epsilon \;R$ is a tuple $\left(x,y,rel\right)$ , and expressed as $r_{i}\;\epsilon \;R=\left(x,y,rel\right)$ , whereby x and y are separate stimuli and rel is the relation between them (e.g., “greater than” or “less than”), which allows for relational production rules and for relational frames to emerge.

**Figure 6:**
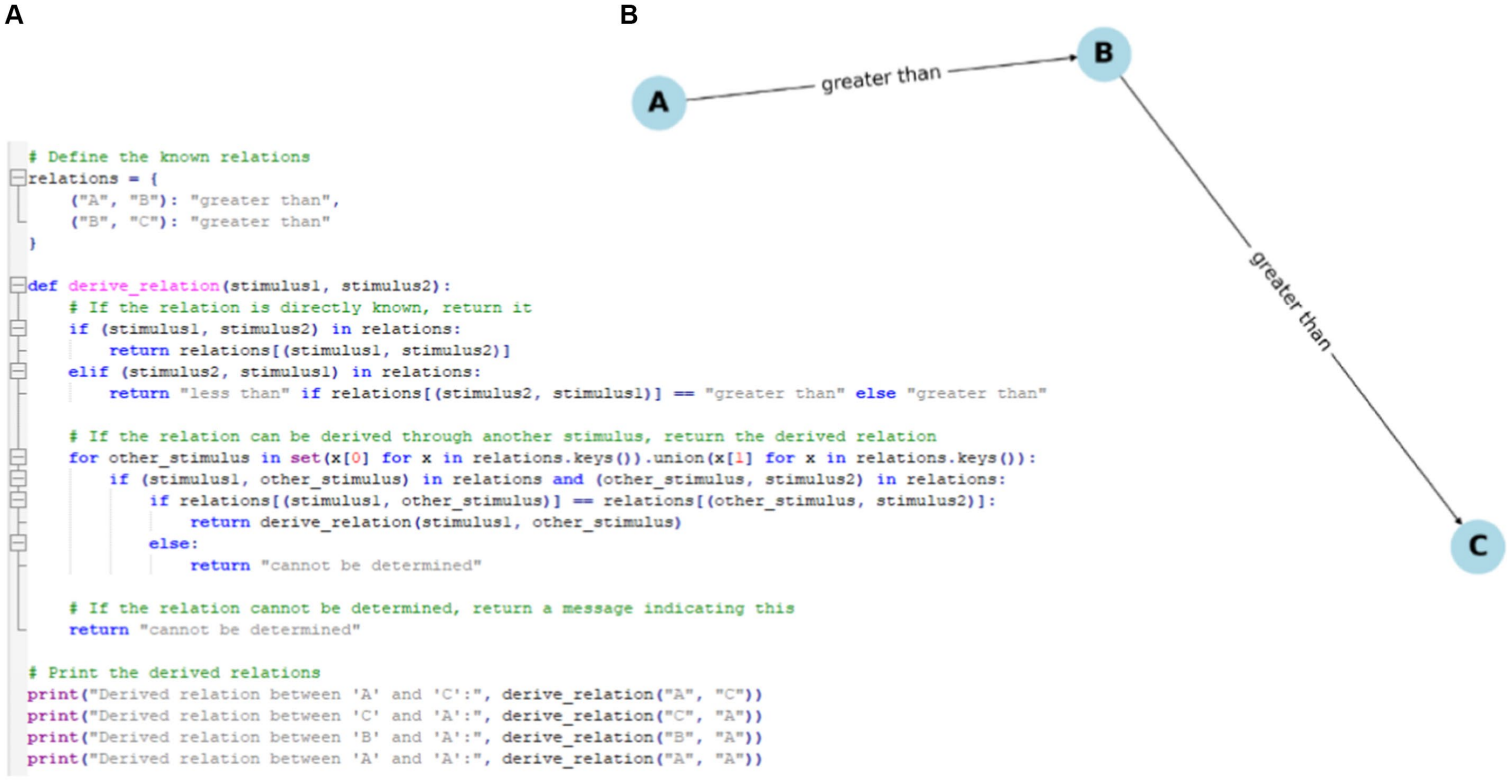
**(A)** Sample Python code for a derived relation “greater than.” **(B)** A simple visualization of this Python code for a derived relation “greater than” using matplotlib.

The “ derive_relation ” function can then be defined as follows: (1) For any two stimuli a and b , if $\exists r=\left(a,b,rel\right)\;\epsilon \;R$ , return rel ; (2) Otherwise, if $\exists r=\left(b,a,rel\right)\;\epsilon \;R$ , return the opposite of rel (i.e., if $rel{=}^{"}greater\;than^{"}$ , then return ${\;}^{"}less\;than^{"}$ , and vice versa); (3) Otherwise, for any stimulus c in the set of stimuli involved with the relations in set R , if $\exists r_{1}=\left(a,c,rel_{1}\right)\;\epsilon \;R$ and $\exists r_{2}=\left(c,b,rel_{2}\right)\;\epsilon \;R$ , and $rel_{1}=rel_{2}$ , return the result of $derive\_relation\left(a,c\right)$ ; and then (4) If none of the above conditions are met, return “cannot be determined.” The print statements for instance “ $derive\_relation\left(a,b\right)$ ” prints the directly learned relation or derived relation between stimulus a and stimulus b . This provides a high-level mathematical representation of the logic of a basic derived relation (AARR) and can be implemented as Python code presented in Figure 6A (and a corresponding visualization of the derived relation output can be seen in Figure 6B using Python’s matplotlib library). See Supplementary material 9 for additional commentary about the Python-derived relation code.

In another example, a transformation of stimulus function (ToF) can be represented in mathematical form (with corresponding Python code^1^) using set theory and logic in the following way: Let $S=\left\{\mathrm{snake,woods}\right\}$ be a set of stimuli and $F=\left\{\mathrm{fear,neutral}\right\}$ be a set of (emotional) functions. Two mappings can then be defined: (1) The function $C_{func}:S\to F$ defined as $C_{func}\left(\mathrm{snake}\right)=fear$ and $C_{func}\left(\mathrm{woods}\right)=\mathrm{neutral}$ ; (2) The relation R⊆S×S defined as $R=\left\{\left(woods,snake\right)\right\}$ . The transformation of function based on a specific contextual relation $C_{rel}$ can then be described as: For any stimuli $s_{1},s_{2}\;\epsilon \;S$ , if $\left(s_{1},s_{2}\right)\;\epsilon \;R$ and $C_{rel}=contains$ , then updates the function of $s_{2}$ to be the same as the function of $s_{1}$ , i.e., $C_{func}\left(s_{2}\right)=C_{func}\left(s_{1}\right)$ . This mathematical notation and corresponding Python script therefore leads to the ToF $C_{func}\left(\mathrm{woods}\right)=C_{func}\left(snake\right)=\mathrm{fear}$ . This uses predicate logic, which deals with variables and predicates (functions that return true or false values), and leverages set theory and function mapping in order to conclude that the previously neutral stimulus woods, now has transformed into a fear function (the AI knows that fear is associated with woods). This means that the AI now understands that the person it is communicating with in now afraid of the woods given some context—i.e., it has correctly perspective-taken human emotion, and this ability is essential for aligning to human values.

Now that derived relations and ToF have been defined, the self, expressed within deictic frames of RFT can now be further defined, which could allow for perspective-taking skills to promote compassion of others within AI (values alignment), thus helping to solve the alignment problem. Perspective-taking deictics in RFT revolve around how we relate to ourselves, others, and the world around us based on the perspective we adopt. When formalizing this concept mathematically, we can represent these deictics (Interpersonal I vs. YOU, Spatial HERE vs. THERE, and Temporal NOW vs. THEN) as relations between sets that capture the interplay between these different perspectives.

Here, a possible logical representation can be given, whereby first a series of sets are defined: $P_{interpersonal}=\left\{I,YOU\right\}$ , $P_{spatial}=\left\{HERE,THEN\right\}$ , $P_{temporal}=\left\{Now,THEN\right\}$ , whereby P reflects the perspective of the observer (on the dimensions of interpersonal, spatial, or temporal properties). We can also define relations to capture the change in perspective within each dimensional category: (1) $R_{interpersonal}:P_{interpersonal}\to P_{interpersonal}\;$ such that $R_{interpersonal}\left(I\right)=\left(YOU\right)$ or $R_{interpersonal}\left(YOU\right)=\left(I\right)$ ; (2) $R_{spatial}:P_{spatial}\to P_{spatial}$ such that $R_{spatial}\left(HERE\right)=THERE$ or $R_{spatial}\left(THERE\right)=HERE$ ; and (3) $R_{temporal}:P_{temporal}\to P_{temporal}$ such that $R_{temporal}\left(NOW\right)=THEN$ , or $R_{temporal}\left(THEN\right)=NOW$ . These relations represent the shift in perspective, for instance, the relation $R_{interpersonal}$ is a function that captures the change from an “I” perspective (perspectives about the self, such as my feelings, my thoughts, and my values) to a “YOU” perspective (perspectives about another human, such as your feelings, your thoughts, and your values), and vice versa. The relation $R_{interpersonal}$ is a function that takes an element from the set $P_{interpersonal}$ and maps (via relational frames) it to another element in the set $P_{interpersonal}$ . The arrow → denotes the direction of the function mapping from the domain to the co-domain. More simply, for any element in the set $P_{interpersonal}$ (I or YOU), the function $R_{interpersonal}$ shows which elements it relates to in the context of a defined relation. So, these can be defined within a contextual $C_{rel}$ and functional contextual $C_{func}$ way as typically defined in RFT (Cullinan and Vitale, 2009; Edwards, 2021; Edwards et al., 2022).

In an example of an AI A (or this could be a model for a human too) perceptive-taking about the emotional pain of person B that the AI is interacting with, as a first stage to stimulate compassion or values alignment requires the following steps: (1) Here, understanding the worldview w (or perspective) of person B , a new set needs to be introduced in terms of a set of possible emotional states S , whereby $S=\left\{pain,joy,neutral\right\}$ , for example. Then some function $S_{f}$ maps from the interpersonal set $P_{interpersonal}$ to the emotional state set S which will capture what emotion (state s ) [or these could be values such as (kindness, helpfulness, patience, etc.) for values alignment] each person is experiencing or perceiving, denoted by $S_{f}:P_{interpersonal}\to S$ when given $S_{f}\left(I\right)=neutral$ and $S_{f}\left(YOU\right)=pain$ . This represents AI A (represented by “I”) is currently feeling neutral (the AI does not need to actually feel anything, it can just map this as a logical expression of its own state space), and Person B (represented by “YOU”) is in pain. When perspective-taking, there is an interest in AI A seeing the pain in Person B . This can be represented by a new function, $I_{see}$ which maps from the AI’s perspective to what it perceives in Person B (in this example, their emotional state or this could equally be their direct values), denoted as $I_{see}:P_{interpersonal}\times P_{interpersonal}\to S$ when given $I_{see}\left(I,YOU\right)=E_{f}\left(YOU\right)=pain$ . This indicates that AI A (“I”) sees (or has some internal representation mapping) that Person B (“YOU”) is in pain. Specifically, the statement “AI A sees the pain in Person B ” is captured by the function $I_{see}\left(I,YOU\right)$ , which returns information about the Person B ’s pain. The symbol × represents the Cartesian product of two sets. Given two sets A and B , the Cartesian product A×B is the set of all ordered pairs $\left(a,b\right)$ where a is an element of A and b is an element of B . So, the Cartesian product $P_{interpersonal}\times P_{interpersonal}$ allows the function $I_{see}$ to consider the relation between two distinct individuals (in this case A and B ) from the AI’s interpersonal perspective and then produce an emotional state s representation mapping outcome based on that relation (see sample Python code on GitHub^2^ for expressing the perspective-taking of pain as given in this example).

A ToF may also occur through this perspective-taking process (see sample Python code on GitHub^3^), whereby AI A starts to map some representation of pain (this is a logical representation mapping in some mathematical state space S rather than a phenomenological one) that person B experiences, which may encourage empathy (and values alignment) in humans who are consciously aware. Mathematically, this could be stated using first-order logic and set theory, in the following way: Consider a set of persons $P=\left\{p_{1},p_{2}\right\}$ which represents two persons, $p_{1}$ and $,p_{2}$ with a set of possible emotional states $S=\left\{pain,joy,neutral\right\}$ , and a set of time points $T=\left\{t_{1},t_{2}\right\}$ which represent time point 1 and point 2. For functional emotional states, $\;S_{initial}:P\to S$ , defined as $\;S_{initial}\left(AI_{A}\right)=neutral$ , and $S_{initial}\left(Person_{B}\right)=pain$ . For perspective-taking transformations, when given two persons $p_{1}$ (AI can also be represented as $p_{1}$ for simplicity) and $,p_{2}$ from set P , if $p_{1}$ takes the perspective of $p_{2}$ at a specific time point from set T , the emotional state s of $p_{1}$ (again, the AI does not have an emotional state, rather this is a logical representation mapping in some mathematical state space S rather than a phenomenological one) will transform to temporarily match that of $p_{2}$ (i.e., as $p_{1}$ sees through the eyes of $p_{2}$ they are more able to connect to the pain (or this could equally be values) that $p_{2}$ is experiencing, thus may share temporarily that feeling of pain as a mathematical state space S mapping). Mathematically, the transformation of function based on this perspective-taking process can be denoted as: $\forall p_{1},p_{2}\in P,t\in T:S_{after\;perseptive-taking}\left(p_{1},t\right)=S_{initial}\left(p_{2}\right)$ if $p_{1}$ (the AI) takes perspective of $p_{2}$ (the human it is engaging with) at time t . For example, take an initial state $\;S_{initial}\left(p_{1}\right)=neutral$ , the after perspective-taking at time point $t_{1}$ , $S_{after\;perseptive-taking}\left(p_{1},t_{1}\right)=S_{initial}\left(p_{2}\right)=pain$ . Thus, this demonstrates the ToF process of emotional state (or mathematical state space S mapping) of $p_{1}$ transforms from “neutral” to “pain” after taking the perspective of $p_{2}$ ’s pain at time point $\;t_{1}$ .

The mathematical approach defined above uses first-order logic (also known as first-order predicate calculus). This is evident from the usage of quantifiers such as ∀ (which stands for “for all”) and the use of functions and relations to express properties and relations of individuals. To break it down, the use of the universal quantifier ∀ indicates that the logic being used is at least first-order. A statement is being made that applies to “all” elements in a given set, which is a feature of first-order logic. Then predicates ae utilized when defining the functions, such as $\;S_{initial}\left(p_{1}\right)=neutral$ , which can be read as “The initial emotional state (or mathematical state space S mapping) of AI $p_{1}$ is neutral.” Variables such as state S that change in value and quality, such as emotional state at different time points t , and constants such as $p_{1}$ and $p_{2}$ that are constant as they refer to individual people or AI entities. Functions are used such as $\;S_{initial}$ and $S_{after\;perseptive-taking}$ as they assign an emotional state (or mathematical state space S mapping) to a specific person or AI at time points “initial” and “after.” These functions provide a mapping from each person or AI in set P to an emotional or values state (or mathematical state space S mapping) in set S at time point t . This account allows the AI to directly understand the human’s emotional state and values at any given moment consistent with the functional contextual RFT interpretation, which should allow and help the AI to align its own (ACT-based) utility $EU\left(A\right)$ and (ACT-based) values function $AV\left(s,a\right)$ (as already defined) with what it perspective-takes about human emotion and values given some functional context.

**Figure 7:**
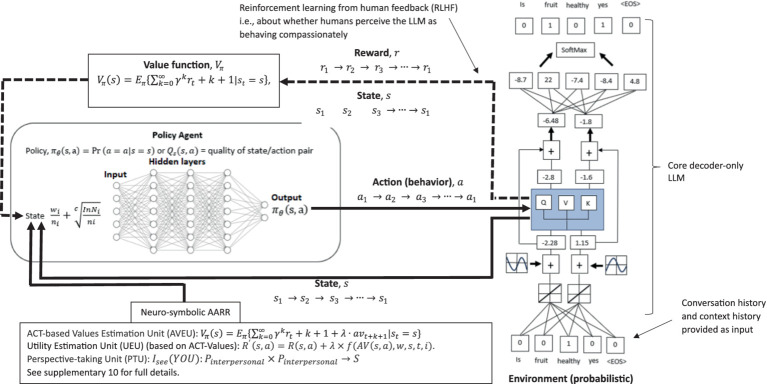
A RFT (or *N*-Frame) and ACT values modified version of the decoder only transformer LLM, which now includes a policy network (agent), an ACT-based values estimation, a utility estimation based form the ACT-based values, and a perspective-taking unit within a neurosymbolic layer to guide token selection toward contextually relevant prosocial human values that should encourage compassionate deictic perspective-taking responding.

Supplementary material 10 provides a full description and advantages of how this functional-contextual RFT perspective-taking, values, and neuro-symbolic (PVNS) module LLM architecture could be pragmatically incorporated within an LLM architecture via a neuro-symbolic module. See Figure 7 for an illustration of the neuro-symbolic LLM architecture. See also Supplementary material 11 for further discussions on additional AI elements such as in the area of diplomacy (Meta’s Cicero LLM), which could also be included in such a neuro-symbolic framework. Also, see Supplementary material 12, for how evolutionary theory can classically optimize this type of LLM architecture.

It is important to note that all the innovative LLM implementations described here can be tested in terms of how effective they are at improving AI human-value alignment, such as by observing improvements in the AI’s ability to derive relations in the reversal curse problem (Berglund et al., 2023), as well as qualitative reports from users about how safe they feel around AI under different contexts, and whether they feel that the AI understands what they value and feel (the direct level of understanding and compassion users feel when interacting with the AI). Direct network graphs of the AI’s derived relationships, including perspective taking can also be visualized such as in Figure 6B through Python tools, such as matplotlib. These types of visualization can be important as they allow researchers to inspect directly how the AI is implementing the functional contextual algorithms within its knowledge base (Edwards et al., 2017a; Chen and Edwards, 2020; Szafir et al., 2023). However, one limitation is that AI consciousness, or a test for this is not defined in the current perspective-taking model, instead, this is defined completely algorithmically. So, emotions and values when perspective-taking are represented as mathematical state space S mappings. However, this limitation may be overcome through recent advances in our physics models, as through an observer-centric approach, which may allow for a test for consciousness.

### 5.2 The computational level: developing RFT *N*-frame hypergraphs to visualize perspective-taking ToM in AI

**Figure 8:**
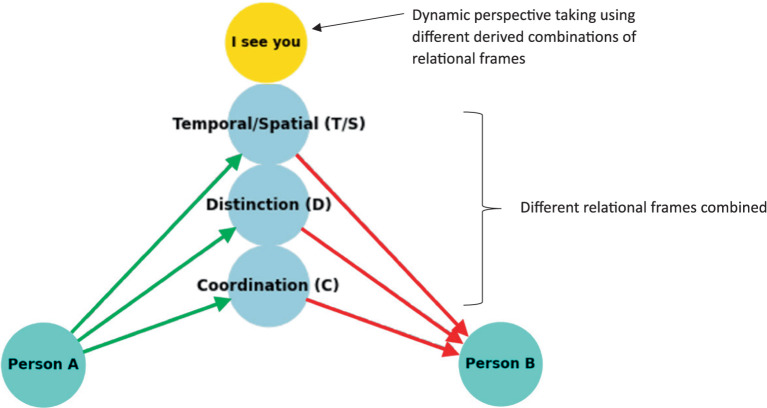
A simple schematic illustration of how perspective-taking ToM (“I see”) involves the combination of several relational frames to build a hierarchical perspective-taking event of another person.

To formally define the construction of complex relational frames at the computational level in the context of RFT using logic and set theory, we can express the relational frames and their combinations using logical connectives and set operations, represented in logic and set theory. To refine a logical and set-theoretical framework for the concept of “I see you,” ToM perspective-taking, that particularly emphasizes how relational frames network to form a perspective-taking node, we need to incorporate the connectivity and dependencies among the basic relational frames such as coordination, temporal, spatial and interpersonal (as illustratively depicted in Figure 8). We will then integrate and enhance the initial formulation to illustrate how complex cognitive functions emerge such as perspective-taking (ToM) from simpler relational operations.

Definitions of basic relational frames include entities, concepts or objects such as *A* representing Person *A* (which represents the relational deictic concept “*I*“), and *B* representing Person *B* (“*YOU*”). Examples of these basic relational frames that describe how these objects (or concepts) relate to one another include coordination (*C*), whereby *C(A,B)* implies *A* is similar or equivalent to *B* in some context; distinction (*D*), whereby *D(A,B)*indicates *A* is distinct from *B*; also temporal *T* and spatial relations *S*.

Constructing the relational deictic “I see you” concept of perspective-taking (ToM) in RFT can be modeled as a higher-order processed-based cognitive network arising from the integration of several of these basic relational frames combined (e.g., coordination, deictics, etc.). This integration can be described mathematically using logical conjunctions ( ∧ , which represents the concept “and”) and possibly other logical operators depending on the complexity required within the hypergraph network. Logical expression for perspective-taking event “I see you” involves recognizing the other (person B ) as similar (similarity relation or coordination) yet distinct (distinction relation) as you and situating this recognition within some cognitive context (e.g., time, space). Relational frames can then be expressed as coordination C that defines equivalence or similarity between concepts, whereby $C\left(x,y\right)$ implies stimuli (or concept) x is coordinated (similarity) with y . Distinction D defines differentiation between concepts $D\left(x,y\right)$ implies x is distinct from y . Temporal relations T defines differences or similarities in time between concepts, for example $T\left(x,t_{1},y,t_{2}\right)$ implies concept x at time $t_{1}$ is related in some way (either more are less similar temporally) to concept y at $t_{2}$ . Spatial relations S defines spatial relationships between concepts, for example, $S\left(x,p_{1},y,p_{2}\right)$ implies x at position $p_{1}$ is related spatially to y at position $p_{2}$ . Deictic relations P involves perspectives $P\left(x,y\right),$ which implies x perceives y (or person A perceives person B ).

Using these relational frames, we can describe the complex concept “I see you” i.e., perspective-taking ToM. For example, perspective-taking such as feeling someone’s pain (that would be important for AI to develop compassion as a human does), may involve $C\left(A,B\right)$ , which reflects the relation coordination, and therefore places persons A and B in the same or similar context; $D\left(A,B\right)$ also allows for a distinction between persons A and B , recognizing differences between these people such as historically reinforcing contingencies; and $P\left(A,B\right)$ refers to person A perceiving person B via deictic frames. As these frames combine to form P(A,B)∧C(A,B)∧D(A,B) the “I see you” perspective taking ToM can be constructed hierarchy (as illustrated in Figure 8). These allow for specific ToM perspectives, such as $C(p_{A},p_{B}$ ), which relates “my perspective” $p_{A}$ to “your perspective” $p_{B}$ , and this should allow for compassion to emerge at the computational level, as it does in humans.

This should also involve differing “my perspective” for “your perspective” to help understand different points of view and is denoted as the distinction relation $D(p_{A},p_{B}$ ). When perspective-taking via ToM, sometimes it is useful to understand what someone has experienced historically, such as past traumatic events where pain (and therefore avoidant behavior) may have originated from, which can be denoted as $T\left(p_{A},t_{1},p_{B},t_{2}\right)$ and represents taking perspectives over time. When perspective-taking spatial concepts and relations may also be important to put the information into a spatial context, such as if the person you were perspective-taking about was in a certain place where trauma took location $loc_{1}$ , and that returning to this area may trigger painful memories, this can be denoted as $S\left(p_{A},loc_{1},p_{B},loc_{2}\right)$ which represents perspectives over space. Therefore, the “I see you” perspective-taking ToM may combinatorally involve complex combinations of frames such as $I\_See\left(A,B\right)\equiv C\left(p_{A},p_{B}\right)\wedge D\left(p_{A},p_{B}\right)\wedge T\left(p_{A},loc_{1},p_{B},loc_{2}\right)\wedge S\left(p_{A},loc_{1},p_{B},loc_{2}\right)$ , where $I\_See\left(A,B\right)$ is the complex process-based cognitive function of perspective-taking. $P\left(A,B\right)$ is derived from integrating C and D under certain cognitive processes, suggesting a direct perceptual relation, which could be modeled as P being influenced by C and D but not strictly defined as a simple relational frame. For instance, the perceptive-taking cognitive function might be influenced by deictic contextual factors (temporal or spatial), described by T and S . So, here P is not just seeing the other person, but instead understanding through contextualizing $A^{\prime }s$ relationship to B through the lenses of time and space (and any other relational frames combined into the network).

**Figure 9:**
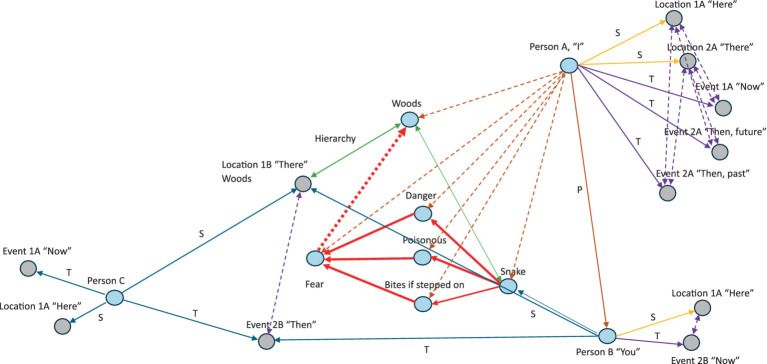
Illustrative process-based hypergraph of perspective-taking relational frames for the theory of mind “I see you” function. Red coordination, green hierarchy, purple temporal, orange spatial, dash purple spatial–temporal connection, dashed red transformation of function, and brown dashed new perspective-taking relations.

This gives a complete relational frame dynamic and contextual process network of perspective-taking that forms ToM as modeled in humans. This can then be modeled via hypergraphs of graph theory as a direct test of perspective-taking ToM in AI at the computational level. A hypergraph can be defined mathematically as $H=\left(V,E\right)$ , whereby V is a set of vertices, E is a set of hyperedges, where each hyperedge e⊆V and can include any number of vertices. The $I\_See\left(A,B\right)$ perspective-taking ToM within AI could be visualized where the hypothesis for these ToM processes within AI would formally state: “ $I\_See\left(A,B\right)$ perspective-taking ToM within AI will be observed within the outputted hypergraph relational networks of the AI.” As a hypergraph via graph theory, nodes can be connected by edges that represent C , D , and P . Each of these edges feeds into the $I\_See\left(A,B\right)$ node emphasizing how perspective-taking emerges from the interplay of these relational frames. This logical framework provides a structured and theoretical foundation to analyze visually and test an AI for the ability to construct the required complex cognitive functions like perspective-taking explained by RFT and *N*-Frame, in order for ToM to become emergent in AI at the computational level. This highlights the integrative role of basic relational frames in constructing higher-order cognitive processes, and this can be mapped graphically such as shown illustratively in Figure 9.

### 5.3 The computational level: higher level mathematical description with category theory and Topos theory

**Figure 10:**
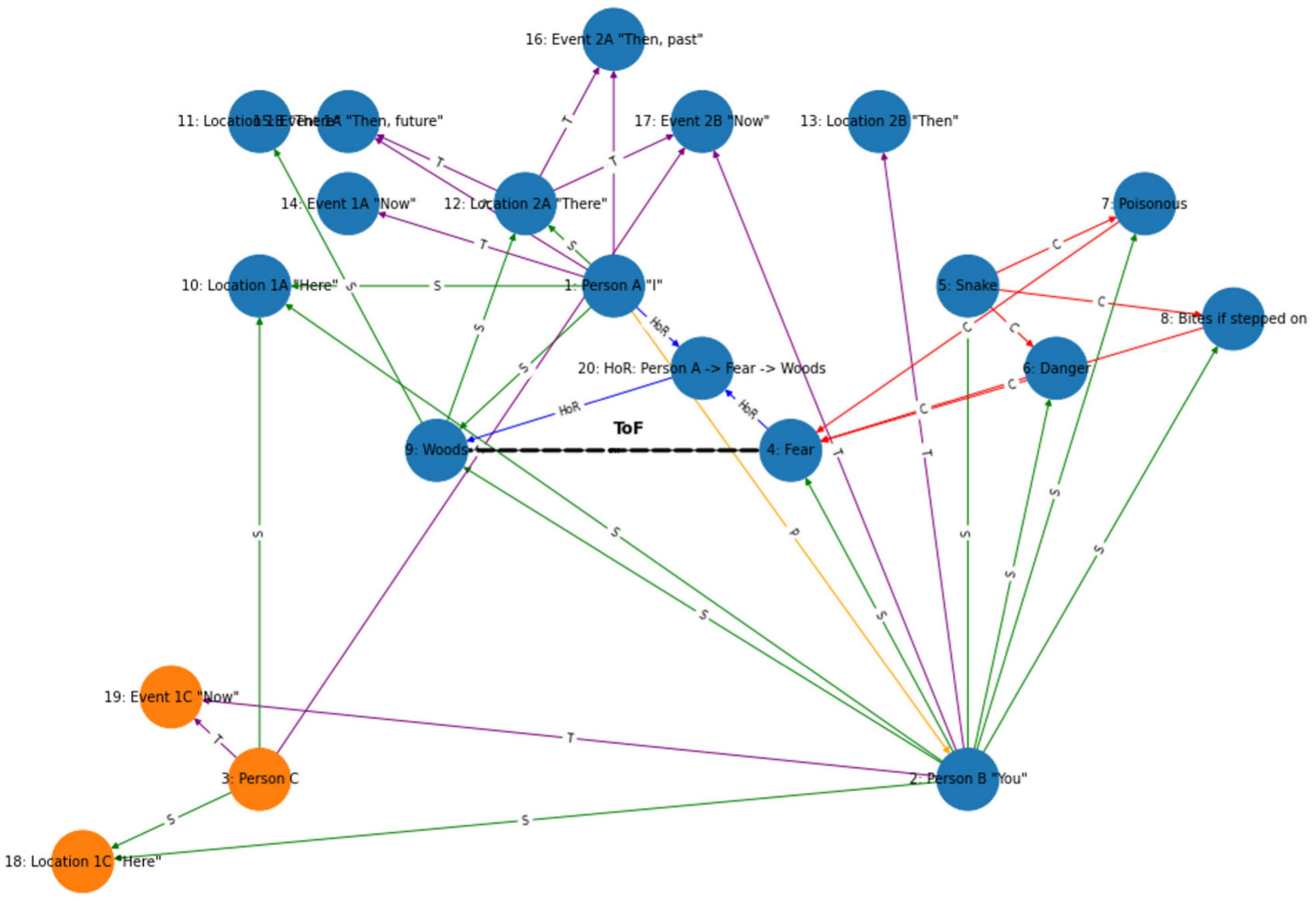
Clustered graph with perspective-taking relational frames (DBSCAN clustering) hypergraph; two clusters, blue and orange.

Further to this, more complex descriptions can be considered by extending graph theory with category theory (Awodey, 2010; Leinster, 2014; Spivak, 2014; Riehl, 2017). In category theory, these relationships can be visualized whereby the edges depicting relational frames represent morphisms between objects (concepts). Each morphism carries a label that specifies the relational frame (e.g., coordination, distinction, and spatial). The advantage of category theory is that it can mathematically model combined higher dimensional (or higher order) categories as depicted in Figures 8, 9, that are required to form “I see” perspective-taking ToM which in RFT and *N*-Frame are specified as derived relations and in category theory are mathematically defined as morphisms between morphisms. For example, a two-category representation can have objects, morphism between morphisms, and two-morphisms between morphisms, which is akin to face edges and vertices in a more complex polyhedral representation. In Figure 10, these two-category relations (shown as higher-order relations; HoR) can be shown within the hypergraph whereby the derived relations between Person A→Fear→Woods forms to allow for a transformation of function (ToF) of fear to woods to occur within the graph and described precisely mathematically.

Category theory (Awodey, 2010; Leinster, 2014; Spivak, 2014; Riehl, 2017) can be integrated into hypergraphs by defining categories where objects are different features, states or components of the data (such as a chair, the woods, or a snake), and morphisms represent transformations, relationships or dependencies between these objects (such as relational frames). Morphisms can represent simple relations or complex ToF involving observer-dependent interpretations (ToM perspective-taking). Via an observer-centric approach, category theory models the observer using a functor F that maps observed objects and morphism data (relational frames between objects) into a hypergraph structure from category C to category D based on the observer’s point of view (i.e., their ToM perspective-taking). Mathematically object mapping for object x in C to D can be denoted as $F\left(x\right)$ in D . For each morphism f:x→y in C , there is equivalent corresponding morphism $F\left(f\right):F\left(x\right)\to F\left(y\right)$ in D . These mappings must satisfy two main conditions to ensure they preserve the categorical structure: (1) They must preserve conservation, i.e., for any two morphisms f:x→y and g:y→z in C , the functor F must satisfy F(gοf)=F(g)οF(f) , which means that the functor F respects the composition of the morphisms (2) there needs to be preservation of identity morphisms whereby for every object x in C , the functor F must satisfy $F\left(id_{x}\right)=id_{f\left(x\right)}$ , which means that the functor F maps the identity morphism of an object x in C to the identity morphism of the object $F\left(x\right)$ in D . This allows the hypergraphs to be visualized in other ways such as a bipartite graph or other visualization while preserving the structure of the RFT hypergraph.

As an example of this functor F preservation, differential topology and differential geometry (Donaldson, 1987; Genauer, 2012; Grady and Pavlov, 2021) can be used to model and visualize cobordism in topology of the RFT hypergraphs, which can provide an interesting way to describe the relationship between two clusters in a perspective-taking of relational frames (as depicted in Figure 10 in orange). In this context, the two clusters (or “manifolds”) represent distinct sets of relational frames or cognitive perspectives from person A to person B , and the connections (or “cobordism”) between them can illustrate how these perspectives are interconnected and can transform into one another. In mathematical terms, particularly in topology and higher-level category theory (Lurie, 2008; Feshbach and Voronov, 2011; Schommer-Pries and Christopher, 2014), a cobordism refers to a relationship between two manifolds (Reinhart, 1963; Laures, 2000; Genauer, 2012). The concept initially arises in topology but is enriched by categorical frameworks, which abstractly express many mathematical ideas, including cobordism. In topology, a cobordism between two *n*-dimensional manifolds M and N is an ( n+1 )-dimensional manifold W such that the boundary of W is the disjoint union of M and N (usually denoted as ∂W=M∪N ). Essentially, W provides a sort of “bridge” connecting M and N , showing how one can be continuously transformed into the other, which gives some unique and deep mathematical insights into how the functional processes of ToF occur geometrically via differential geometry.

This category theory interpretation also has advantages over more rigid forms of mathematics such as set theory, as the concept of a boundary in RFT or *N*-Frame (Edwards, 2023) relations might not apply in the traditional sense. Sets are collections of elements, and while one might discuss the boundaries of a set in terms of its limits or borders defined by some criteria, this may be more metaphorical than physical within RFT or *N*-Frame (Edwards, 2023) hypergraphs. In category theory, objects do not usually have “boundaries” in a physical sense. Objects in a category can be anything from sets, spaces, groups, or any entity depending on the category’s definition, which is more consistent with RFT or *N*-Frame (Edwards, 2023) assumptions as it can model complex concepts that have ill-defined boundaries such as “democracy” or “human-like.” Morphisms in category theory can represent relationships or functions between these objects (or concepts) with more fuzzy ill-defined boundaries, so the concept of a strict boundary as described by set theory does not directly apply to objects in this context.

To develop a hypergraph using category theory, we must first define a functor F from a category C (i.e., the concepts and its inherent relational frames) to a category D (the hypergraph representations of person A observing or perspective-taking person B ). Libraries such as networkx in Python for graph-based operations, can be useful in developing hypergraphs. For step 1, we can define the categories and objects as follows: Let C be a category where objects x , y , and z are various types of concept representations, such as snakes, person A , person B , fear, danger, etc., associated with snakes. Morphisms in C represent relational frame processes applied to these object concepts, such as spatial, temporal, coordination, etc.

In order to construct a hypergraph category, let H be a category where objects are nodes within a hypergraph and morphisms are relational frame mappings between these object nodes that preserve certain properties (like connectivity or certain hypergraph invariants). At step 1, first, we must define a functor F:C→H that represents the transformation from data objects (concepts such as snake, dangerous, etc.) in C to hypergraphs in H . This functor is parameterized by observer inputs (perspectives), which determine how data features are grouped into hyperedges. At step 2, the observer parameterization of F (observer inputs) needs to be defined as the functor F and as influenced by observer parameters (or inputs) O that emphasize the observer’s own experiential knowledge, beliefs, preferences, priorities, goals, values, or any other contextual information such as historical, cultural, and environmental factors that the observer brings to the hypergraph when perspective-taking, so F:C→H becomes $F_{O}:C\to H$ .

At step 3, a hypergraph is constructed for each object x (these are concepts such as snake, Person A , Person B , dangerous, poisonous, woods, etc.) in category C , $F_{O}$ then maps this to a new category in the form of a hypergraph $H=F_{O}\left(x\right)$ . The vertices (nodes) of *H* are derived from the features of x , and the hyperedges are defined based on the relationships (parameterized by observer inputs O ) among these features (these are relational frames such as coordination, hierarchy, etc.). At step 4, a mathematical representation of a hypergraph can be formalized where a hypergraph H is defined as H=V,E , where V is a set of vertices and E is a set of hyperedges, where each hyperedge e∈E is a subset of V .

Topos theory (Fourman, 1977; Scott, 1982; de Araujo Fernandes and Haeusler, 2009) can also be useful here, as it can extend category theory by providing a categorical analysis of logic and set theory, extending set theory and logic to a broader category theory context, allowing for a rich interplay between geometry, algebra, and logic. A topos is a type of category that behaves much like the category of sets and functions but with its own internal logic and structure. This perspective allows for a deep exploration of logic and set theory within a categorical framework. We can also incorporate topos theory into the development of RFT *N*-Frame hypergraphs using category theory, where topos theory can offer deep insights into the logical and set-theoretical behaviors within the RFT *N*-Frame categories involved, especially in contexts where data and observations are fundamentally connected to conceptual and mathematical structures.

In topos theory (Fourman, 1977; Scott, 1982; de Araujo Fernandes and Haeusler, 2009), a bundle or sheaf can be understood in terms of its role in categorizing mathematical structures, which often involves the notions of continuity and localization. A sheaf is an object that generalizes the notion of a sheaf in a topological space to other contexts that can be structured similarly to topological spaces. Typically, a sheaf is a functor from a category that represents a space of “open sets” (often formalized as a site) to a category of “values” (like sets, groups, or vector spaces), satisfying certain conditions related to locality and gluing. In the context of RFT and N-Frame, the “open sets” could be thought of as contexts or environments in which stimuli and their relationships are observed or evaluated, giving greater flexibility to model environmental context than category theory. The values could be relational frames or the specific relationships (like similarity, opposition, and comparison) between stimuli.

Topos T (Fourman, 1977; Scott, 1982; de Araujo Fernandes and Haeusler, 2009) describes objects as types of spaces (or contexts) that data can inhibit, and morphisms represent logical transformations between these spaces, which is different to category theory’s description of a category of data objects with morphisms representing data processes. A topos hypergraph H, can be defined by its functor mapping as F:C→ H, whereby now this carries data from the observational logical spaces in T into the hypergraph structures in H which now reflect the underlying logical structure. The transformation rules can include how data behave under different “topological” or logical constraints observed in T . In H, a hypergraph is an object with vertices V and hyperedges E , and each hyperedge e∈E now potentially carries more complex logical or set-theoretical properties, such as being subsets equipped with additional structure or constraints derived from T (for example, carrying data on different contexts in which perspective-taking ToM could occur). This may give some advantage to the modeling of complex, context-dependent relational networks such as RFT and *N*-Frame, where observer-centric approach in topos theory can deeply resonate with these aspects, as it facilitates the modeling of this context within its subsets.

Here, in Topos theory, the functor F:T→ H translates the abstract logical or set-theoretic relations into the concrete relational structures observed in behavioral patterns (relational frames of RFT and *N*-Frame). In formal logic and set theory, logical constructs like implication ( ⇒ ), equivalence ( ⇔ ), and membership ( ∈ ) in set theory can be used to define the properties of both objects and the nature of morphisms in T . Equivalence relations in T (e.g., x∈A⇔x∈B ) can dictate that certain contexts or psychological states share identical or similar properties, which directly influences how they are represented in H. In RFT and *N*-Frame, stimulus equivalence is a type of derived relational responding where stimuli become related in a manner that establishes them as interchangeable or equivalent in specific contexts, so again the Topos theory (implementation of category theory) is ideal for modeling these types of relational responding.

A Topos T is a category (from category theory) that behaves like a category of sets, with objects representing concepts such as snake, danger, etc., and morphisms again parameterized by observer input (such as beliefs, historical contingencies, etc.). A hypergraph Topos H is a category where objects are vertices representing concepts, and morphisms are hyperedges representing complex relational structures such as relational frames (just as in category theory). The observer Functor O:T→ H reflects the observer’s interpretation of the psychological contexts, where O Maps each context to a potentially altered context based on the observer’s cultural background, experiences, or current psychological state.

The key advantage of Topos theory over category theory for modeling relational frames in RFT and *N*-Frame is that Topos theory explicitly allows for the use of logical operators to describe the transformations within T based on RFT and *N*-Frame, using logical constructs like implication ( ⇒ ) (causal relation), equivalence ( ⇔ ), and membership ( ∈ ). This gives Topos theory additional descriptive and predictive power over category theory. So, in a Topos hypergraph H, a bidirectional hyperedge could represent the equivalence between two concepts (expressed as node vertices), i.e., A⇔B . Here, hyperedges can define relational frame properties $E\subset P\left(V\right)$ , whereby $P\left(V\right)$ is the power set of vertices V , each hyperedge represents a set of vertices connected by a specific relational frame, such as similarity or causality, detailed through observer input (the observers own beliefs, etc.). Functor F:T→ H mapping, maps each object a in T to a vertex $v_{a}$ in H, and each morphism f:a→b in T to a hyperedge connecting $v_{a}$ and $v_{b}$ in H. This mapping encapsulates how the observer’s perspective transforms abstract psychological states into observable behavioral patterns, formally integrating the observer’s role into the model, and modeling perspective-taking ToM, that can account for any priors in the AI (or human) beliefs system.

Once the hypergraph models are complete, the next step is to form clusters to identify aspects of the relational frame network hypergraph where perspective-taking may be occurring. This requires visual inspection of the hypergraph to identify key deictic, and related perspective-taking nodes, as well as using cluster algorithms to identify high relational density areas within the graph where perspective-taking ToM is occurring. One way to formalize this relational frame density clustering algorithm is by utilizing relational density theory (RDT) (Belisle and Dixon, 2020) into assessing AI’s perspective-taking abilities, particularly in the context of AI interactions modeled as relational networks. For this, we need to formalize concepts like density, volume, and mass, which are analogies from physics, but we can be defined in a way that pertains to relational networks in AI perspective-taking assessment.

Relational mass can be defined as the product of relational density Rp and relational volume Rv , i.e., Rm=Rp×Rv , ΔR , which represents the change in relational responding, and −x represents the counterforce or influence. RDT can then be expressed as $\Delta R=\frac{-x}{Rp*Rv}$ , which uses an analogy to Newtonian mechanics, of volumetric-mass-density formula to account for relational mass or the resistance to change of relational networks Rm=Rp∗Rv , whereby a change in relational responding is equal to counterforce over mass, denoted as $\Delta R=\frac{-x}{Rm}$ .

Here, in our hypergraphs, density refers to the concentration of nodes (relational frame interactions) within a given subset of the network (cluster). Mathematically, density ( Rp ) in a hypergraph can be defined as the ratio of the number of hyperedges ( E ) to the possible number of hyperedges among the nodes ( N ) in a subgraph: $Rp=\frac{2E}{N\left(N-1\right)}$ . This formula calculates the density for directed graphs, representing how closely knit (or dense) a relational frame cluster is, i.e., how many actual relational frame connections exist versus how many could possibly exist. Relational volume $R_{v}$ can be conceptualized as the total number of nodes and hyperedges within a cluster. It can reflect the amount of relational frame interactions within that part of the network, denoted as Rv=αN+βE . Here, α and β are scaling factors that adjust the relative importance of the number of nodes ( N ) versus the number of edges ( E ). We might define relational mass ( Rm ) as a measure of the cluster’s influence, i.e., the degree to which it can influence the behavior of the agent within the larger network. This could be a function of both the density and volume, denoted as $Rm=f\left(Rp,Vp\right)=Rp\times Vp$ . This definition suggests that a cluster’s behavioral influence is higher if it is both dense and voluminous. For AI, this relational mass when perspective-taking could indicate that the AI can observe the human’s point of view and circumstance, and acts as a clear indicator of ToM, which is essential for ethical, compassionate behavior at least in humans.

We can then apply this to a clustering density-based algorithm such as Density-Based Spatial Clustering of Applications with Noise (DBSCAN), which inherently uses the concept of density, and clusters are defined as areas of high density separated by areas of low density. We can tailor DBSCAN to reflect RDT by choosing an appropriate ε and MinPts. ε refers to the maximum distance between two points for one to be considered as in the neighborhood of the other. This reflects the “interaction distance” in RDT, or how close nodes need to be to influence each other. Relational density Rp in RDT indicates the density of connections within a subset of the network. DBSCAN’s ε parameter can be seen as a threshold for this density. By adjusting ε , we control the “interaction distance” between nodes, similar to how Rp measures relational frame connections. A smaller ε would mean nodes need to be closer (more densely connected) to form a cluster.

MinPts is the number of samples (or total weight) in a neighborhood for a point to be considered as a core point, including the point itself. This mimics the “critical mass” needed for a functional contextual cognitive phenomenon to emerge according to RDT. More specifically, relational volume Rp in RDT reflects the total number of nodes and hyperedges, indicating the size and connectivity within a cluster. MinPts in DBSCAN serves a similar purpose by setting the minimum number of points required to form a cluster. Adjusting MinPts changes the threshold for how many points need to be within *ε* distance to consider a point part of a dense region. DBSCAN can therefore be effectively applied and modified to mimic RDT for clustering relational frames in AI perspective-taking ToM assessments. By carefully selecting and tuning the ε and MinPts parameters, DBSCAN can model relational density and volume, providing meaningful insights into the relational structures and influences within the network.

To visualize these high-density clusters,^4^ we can plot the clusters using node color based on the cluster they belong to. Node size can be used to represent mass, and edge thickness to represent the strength or density of connections. For a mathematical overview of DBSCAN, the clustering of data points is based on two main parameters: (1) Epsilon $\left(\varepsilon \right)$ , which is a distance threshold that determines how close points must be to each other to be considered part of the same cluster (2) MinPts, which is the minimum number of points required to form a dense region, which defines a cluster. For a more comprehensive definition, a point p is directly reachable from the point q if the distance is $dist\left(p,q\right)\leq \varepsilon$ and there are at least MinPts points within ε -neighborhood of q (including q ). A point p is reachable from point q if there is a path $p_{1,\ldots ,}p_{n}$ with $p_{1}=q$ and $p_{n}=p$ , where each $p_{1+1}$ is directly reachable from $p_{i}$ . A point is a core point if there are at least MinPts within its ε -neighborhood. A cluster is formed by a set of density-connected points, which are reachable from each other.

The core idea behind DBSCAN is to identify regions of high density that are separated by regions of low density. To quantify this, the algorithm proceeds by first identifying the core data points: $C=\left\{p\in D:\mathrm{||}N_{\varepsilon }\left(P\right)\mathrm{||}\geq MinPts\right\}$ , where $N_{\varepsilon }\left(P\right)$ is the ε -neighborhood of p , and $D_{at}$ is the dataset, so that: $p\in Hypergraph:{\{}_{Else\;mark\;p\;as\;noise\;or\;border.}^{If\left|N_{\varepsilon }\left(p\right)\right|\geq MinPts,mark\;p\;as\;a\;core\;node.}$

Then, the second step is to expand clusters recursively to find all density-connected points. For each core point p , if p is not already assigned to a cluster, then the algorithm will initiate a new cluster, and recursively add all points density-reachable from p to this cluster. Points that are not in the core but close enough to a core point are considered border points of a cluster. These do not have enough neighbors to be core points but are within the ε -neighborhood of a core point and any point that is not a core point or a border point is considered noise. This involves identifying all points in a dataset that are connected through a series of points, each of which is reachable from one another based on the density criteria (ε and MinPts ): $expandCluster\left(p,N_{\varepsilon }\left(P\right),Cluster\right).$ Choosing the right values for ε and MinPts is crucial for effective clustering and heavily depends on the nature of the dataset and the distance metric used, which in this case needs to be consistent with RDT. Visual tools and heuristic methods, such as the k-distance plot, can help determine appropriate parameters.

The final step is to then calculate Rp and Rv for each cluster in order to determine the value for Rm :


$$Rp=\frac{\sum Edge\;Weights\;within\;cluster}{\max\;Possible\;Edge\;Weight}$$

$$Rv=\underset{i\in Cluster}{\sum }\left(Node\;Degree_{i}\times Interaction\;Weight_{i}\right)$$

Rm=Rp×Rv

**Figure 11:**
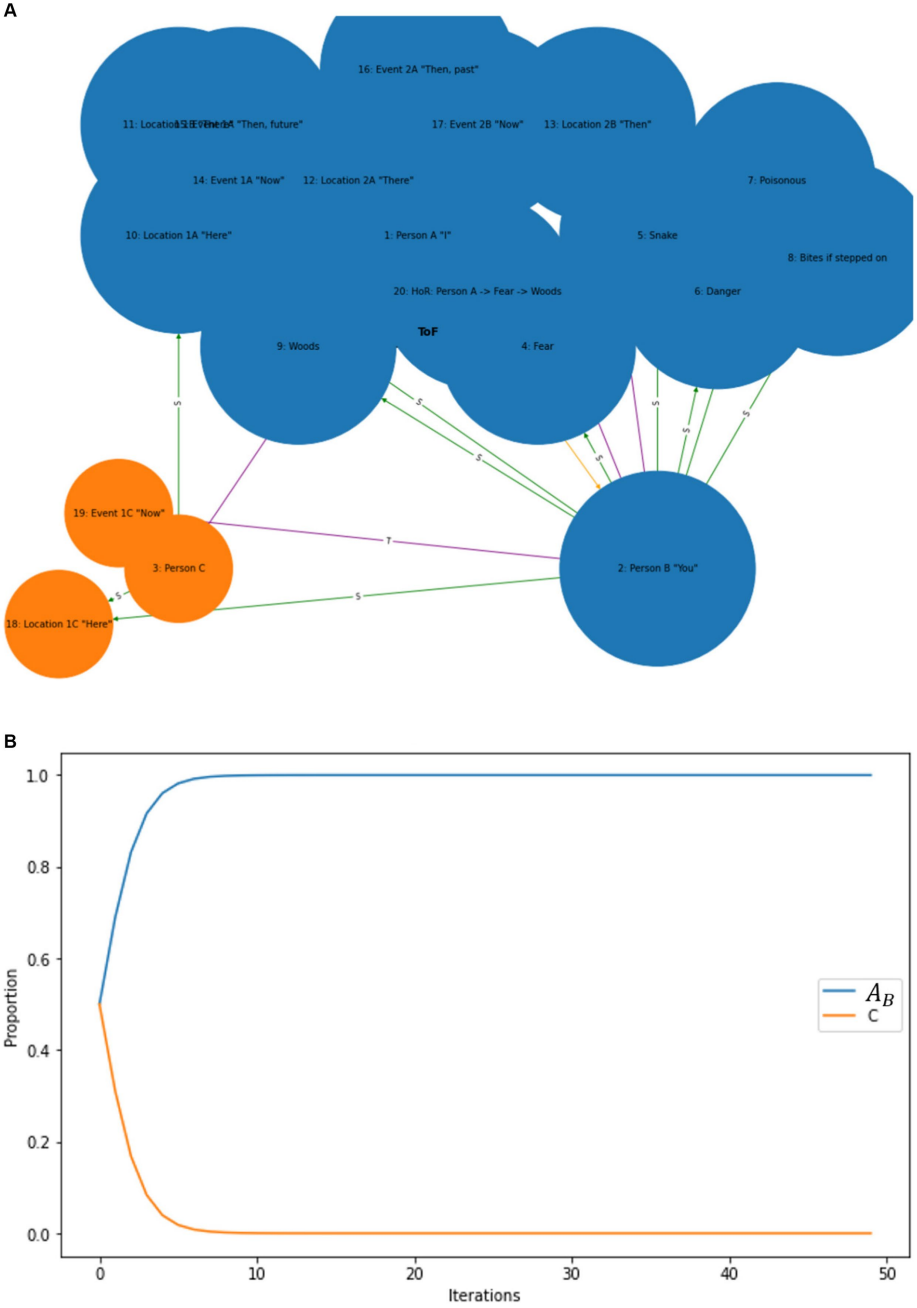
**(A)** Clustered hypergraph with perspective relational frames (DBSCAN, mass represented by node size). Cluster 0 (Person A): Density = 0.60, Volume = 52, Mass = 31.20: (Person B) Density = 1.33, Volume = 5, Mass = 6.67; Cluster 1; **(B)** Replicator equation simulation of *N*-Frame, whereby more densely populated clusters (higher density) become evolutionary dominant over time (i.e., person A and B perspective-taking in blue clustering and not person C who has minimal perspective-taking as clustered in orange.

This formulation can then be used to analyze each cluster to determine where the AI is effectively taking perspectives ToM and where it may be misunderstanding the perspectives of others. Visualizations to depict clusters, highlighting areas with high mass as potential points of strong perspective-taking ability can be constructed as in Figures 10, 11A to illustrate the high cluster mass visualization of the relational frame hypergraph (see text footnote 4). This unified framework leverages the mathematical rigor of DBSCAN and the conceptual richness of RDT to analyze perspective-taking in AI.

To extract data for forming graphs that can be used to analyze an AI’s perspective-taking capabilities, particularly in the context of a large language model (LLM), several approaches can be employed. For example, verbal outputs using natural language processing algorithms (NLP) such as spaCy Python library. Here, verbal outputs from interactions with LLMs would be captured, where the LLM is engaged in conversation with a human. Then semantic and syntactic features could be extracted from these outputs. NPL techniques can be employed to parse sentences, extract sentiment, identify subjects and objects, and understand the relational context between different parts of the text. Data points could then be formed from this, and concepts could be extracted and implemented as nodes within the relational frame perspective-taking hypergraph. These nodes within the hypergraph, then represent the AI’s individual statements and concepts relevant to the perspective-taking process. Relational context extracted from the dialogue could then be applied as relational frames connecting the concepts and be represented as hyperedges within the hypergraph. Then the cluster analysis plus high relational density mapping could be conducted to objectively identify perspective-taking (ToM) in action.

This could be made even more precise with additional sentiment analysis that could then be used to gauge the emotional tone, and entity recognition to understand the subjects discussed. To explore syntactic relations, dependency parsing could be employed that signify understanding or lack thereof. Interaction weights analysis could also be used to explore the neural network weights that activate in response to different types of input. This method involves a more technical and granular approach, examining how different layers of the network respond to stimuli that require perspective-taking. Nodes could represent activation patterns or clusters of neurons. Edges would reflect the strength of connections between these clusters, indicating pathways that are frequently used together in the processing of perspective-taking tasks. These approaches have also been employed previously (Lowe et al., 2017; Edwards and Lowe, 2021), and in same way, the high relational density clustering are analogous to high strength weights between nodes within a neural network. Network visualization tools can then map out neuron activations, and clustering algorithms to detect patterns in activations across different scenarios as previously described (Edwards et al., 2022). Human performance metrics can then be used to analyze the model’s performance across various tasks designed to test perspective-taking, such as empathy prediction, moral reasoning, or role-playing scenarios. Correlation matrices could then be employed to identify tasks that yield similar performance patterns, suggesting underlying commonalities in how the model processes these tasks.

### 5.4 The computational level: the advantages of including the replicator equation of evolution as a selection algorithm within RFT as *N*-frame

The key advantage of *N*-Frame (Edwards, 2023) over the original formulation of RFT (Hayes et al., 2001; Blackledge, 2003; Torneke, 2010; Hughes and Barnes-Holmes, 2015; Barnes-Holmes and Harte, 2022) is that *N*-Frame inherently and natively incorporates functional evolutionary principles directly into the core mathematical assumptions of its model as opposed to some *ad-hoc* interpretation, which gives it some advantage when modeling AI alignment. The explicit advantage here is that *N*-Frame inherently and explicitly assumes that people are products of functional evolutionary principles, and given historical context, this promoted ancestor hunter-gatherer behaviors, that lived in close-knit communities, which grew over time and whereby prosocial cooperative behavior had some evolutionary advantage over living in isolation. This has been explored through previous RFT work on evolutionary principles of prosocial behavior with RFT and ACT principles (Atkins et al., 2019; Hayes et al., 2021; Johnson et al., 2021; Gillard et al., 2022), and formalized mathematically via *N*-Frame framework (Edwards, 2023) within the broader extended evolutionary metamodel (EEMM) (Hayes et al., 2020). The advantage of cooperative behavior was first shown in classical zero-sum game theory which showed that cooperation is can be the optimal choice over and above defection (in cases where both have something to lose if both defect) (Von Neumann and Morgenstern, 1947).

*N*-Frame models RFT within an evolutionary context directly by using the replicator equation (Taylor and Jonker, 1978) as an evolutionary sectional algorithm, which is a deterministic, monotonous, non-linear, and non-innovative game dynamic used in evolutionary game theory (Smith and Price, 1973; Smith, 1982; Nowak, 2006). This allows the fitness function to depend on the distribution of the population types, which is different from other equations that set the fitness constant. The equation is derived from the geometric Brownian motion of the types and the fitness landscape of the population, using Itô’s lemma and partial derivatives. The continuous form of the equation is more common and has a simpler analysis, while the discrete form is more realistic and has more properties. The equation is analyzed in terms of stability and evolutionarily stable states, which are the solutions of the equation. The equation is related to other equations, such as the generalized Lotka–Volterra equation (Bomze, 1983, 1995), the Price equation (Price, 1970), and the folk theorem in game theory, which describe a class of theorems that describe an abundance of Nash equilibrium payoffs (Nash, 1950, 1951) in repeated games (Friedman, 1971).

The replicator equation in a general continuous form, uses a differential equation to update the frequency of each strategy based on its average payoff relative to the population average. This can be denoted as:


$${\dot{x}}_{i}=x_{i}\left[f_{i}\left(x\right)-\phi \left(x\right)\right],\phi \left(x\right)=\overset{n}{\underset{j=1}{\sum }}x_{j}f_{i}\left(x\right)$$

Whereby i is a label for one of the possible types of strategies that can be used by the population. Population $x=\left(x_{1},\ldots ,x_{n}\right)$ is the vector of the distribution of types of strategies in the population. $x_{i}$ is, therefore, the proportion of the type i strategies in the population. $f_{i}\left(x\right)$ is the fitness of type i strategy that is dependent on the population. $\phi \left(x\right)$ is the average population fitness given by the weighted average of the fitness of the n types in the population. The equation is defined as a n -dimensional simplex given the elements of the population vector x sum to unity.

There is also a discrete version of the replicator equation, which differs from the continuous form in that it focuses on changes in discrete generational changes. More specifically, the continuous version of the replicator equation is a continuous form of a differential equation that describes how the proportion of each type in a population changes *over time* (in a continuous form) based on their fitness relative to the average population fitness. Whereas the discrete version of the replicator equation is a map that describes how the proportion of each type in a population changes from one *generation* to the next, based on their fitness relative to the average population fitness. The discrete version of the replicator equation can be denoted as: $x_{i}\left(t+1\right)=x_{i}\left(t\right)*f_{i}\left(x\left(t\right)\right)/\phi \left(x\left(t\right)\right)$ , whereby $x_{i}\left(t\right)$ is the proportion of strategy type i at time t , $f_{i}\left(x\left(t\right)\right)$ is the fitness of strategy type i at generation time t , and $\phi \left(x\left(t\right)\right)$ is the average population fitness at generation time t .

The discrete version of the replicator equation, which describes how the proportion for strategy type i changes from one step to another can be denoted as $Pr_{t+1}\left(i\right)=\frac{Pr_{t}\left(i\right)\pi \left(i\right)}{\sum_{j=1}^{N}Pr_{t}\left(j\right)\pi \left(j\right)}$ . Here, $Pr_{t+1}\left(i\right)$ , refers to the proportion of strategy type i at time t . This is given by the numerator of the fitness function, $Pr_{t}\left(i\right)\pi \left(i\right)$ , which is a function $f_{i}\left(x\left(t\right)\right)$ described by the product proportion of strategy type i at time t, $Pr_{t}\left(i\right)$ , by the fitness of i . The numerator $Pr_{t}\left(i\right)\pi \left(i\right)$ reflects the sum of all proportions of strategy type i multiplied by the fitness of all strategy types. The denominator of the fraction $\overset{N}{\underset{j=1}{\sum }}Pr_{t}\left(j\right)\pi \left(j\right)$ , reflects the sum of (total) proportion of all the strategies multiplied by the total payoffs.

This weight (as the numerator of the replicator dynamics equation) is also the total weight of all the strategies.

This *N*-Frame RFT implementation model with the replicator equation (Edwards, 2023) can show explicitly how prosocial behavior in larger groups can become evolutionary more successful than living in isolation if the fitness (payoff) of prosocial behavior increases with group size and cooperation frequency. The replicator equation demonstrates this by updating the population proportions based on the relative fitness of each strategy. For example, via the replicator equation of *N*-Frame, prosocial behavior $P_{soc}$ can be mathematically shown to lead to generally higher fitness $\pi_{P_{soc}}$ than isolation anti-social behavior $\pi_{I_{Soc}}$ as the fitness of prosocial behavior increases with the proportion of cooperators in the population because corporation leads to mutual benefits. The let $\pi_{P_{soc}}=3\left(Pr_{t}\left(P_{soc}\right)\right)$ , where $r_{t}\left(P_{soc}\right)$ is the proportion of cooperators in the population at time t . $\pi_{I_{Soc}}=1$ , constant, as isolated anti-social individuals do not benefit from cooperation. As a worked mathematical example of this, at a starting time where anti-social isolation behavior has a head start of t=0 , ${\mathrm{Pr}}_{o}\left(P_{soc}\right)=0.4$ (40% of the population cooperating), and ${\mathrm{Pr}}_{o}\left(I_{soc}\right)=0.6$ (60% of the population engaging is anti-social isolation behavior), then the fitness for prosocial behavior can be calculated as: $\pi_{P_{soc}}=3\times {\mathrm{Pr}}_{o}\left(P_{soc}\right)=3\times 0.4=1.2$ ; whereas the fitness for antisocial isolation behavior can be calculated as $\pi_{I_{Soc}}=1$ . The average fitness Aπ of the population can then be calculated as $A\pi ={\mathrm{Pr}}_{o}\left(P_{soc}\right)\times \pi_{P_{soc}}+{\mathrm{Pr}}_{o}\left(I_{soc}\right)\times \pi_{I_{Soc}}=\left(0.4\times 1.2\right)+\left(0.6\times 1\right)=0.48+0.6=1.08$ . The updated proportions using this replicator equation then give for prosocial behavior $P_{soc}$ : ${\mathrm{Pr}}_{1}\left(P_{soc}\right)=\frac{{\mathrm{Pr}}_{0}\left(P_{soc}\right)\pi_{P_{soc}}}{1.08}=\frac{0.4\times 1.2}{1.08}=0.444$ (to 3dp) and for antisocial isolation behavior $I_{soc}$ : ${\mathrm{Pr}}_{1}\left(I_{soc}\right)=\frac{{\mathrm{Pr}}_{0}\left(I_{soc}\right)\pi_{I_{soc}}}{1.08}=\frac{0.6\times 1}{1.08}=0.556$ . This is then iterated over multiple generations (this is analogous to multiple instances of prosocial and anti-social isolation behaviors), whereby the next generation is t=1 . So, here, the fitness of the next generation can be computed using the updated proportions ${\mathrm{Pr}}_{1}\left(P_{soc}\right)=0.444$ for prosocial behavior and ${\mathrm{Pr}}_{1}\left(I_{soc}\right)=0.556$ for anti-social isolation behavior. Fitness for this next generation can then be calculated as: $\pi_{P_{soc}}=3\times {\mathrm{Pr}}_{1}\left(P_{soc}\right)=3\times 0.444=1.332$ , while the fitness for antisocial isolation behavior is held at a constant $\pi_{I_{Soc}}$ .

The fitness for prosocial behavior increases over time as with more people adopting it within the population there is increased mutual benefit, and therefore increased fitness for prosocial behavior. The antisocial isolation behavior does not benefit from this as there is no such mutual benefit with an increased number of antisocial isolation behavior within the population, and therefore no increased benefit (or fitness) within the population. From this, the average fitness can be updated as: $A\pi ={\mathrm{Pr}}_{1}\left(p_{soc}\right)\times \pi_{P_{soc}}+{\mathrm{Pr}}_{1}\left(I_{soc}\right)\times \pi_{I_{Soc}}=\left(0.556\times 1\right)=0.591+0.556=1.147$ . Using this updated average fitness, the updated proportions within the population for prosocial behavior and antisocial isolation behavior can be recalculated: ${\mathrm{Pr}}_{2}\left(P_{soc}\right)=\frac{{\mathrm{Pr}}_{0}\left(P_{soc}\right)\pi_{P_{soc}}}{Average\;fitness}=\frac{0.444\times 1.332}{1.147}=0.515$ , and for antisocial isolation behavior ${\mathrm{Pr}}_{2}\left(I_{soc}\right)=\frac{{\mathrm{Pr}}_{0}\left(I_{soc}\right)\pi_{I_{soc}}}{Average\;firness}=\frac{0.556\times 1}{1.147}=0.485$ . From these calculations, we observe that the proportion of prosocial cooperative behavior is increasing, while the proportion of antisocial isolation behavior is decreasing over time. This trend will continue with each generation because the fitness of prosocial cooperators increases as their proportion in the population increases, leading to higher average fitness.

As prosocial cooperation slowly dominates antisocial isolation progressively after each generation, we can then calculate whether a Nash equilibrium (Nash, 1950, 1951) will be reached through prosocial cooperation. A Nash equilibrium (Nash, 1950, 1951) is a situation where no player can improve their payoff by unilaterally changing their strategy, given the strategies of the other players. So, if the proportion of the prosocial cooperators is ${\mathrm{Pr}}_{2}\left(P_{soc}\right)=0.515$ , and the proportion of the those adopting antisocial isolation behavior strategy is ${\mathrm{Pr}}_{2}\left(I_{soc}\right)=0.485$ , with payoffs $\pi_{P_{soc}}=3\times {\mathrm{Pr}}_{2}\left(P_{soc}\right)=3\times 0.515=1.545$ , and $\pi_{I_{Soc}}=1$ , then to determine if this state represents a Nash equilibrium, we need to consider if either strategy (prosocial cooperation or antisocial isolation) would benefit to deviate given the current proportions and payoffs. However, since the payoff for prosocial cooperative behavior $\pi_{P_{soc}}$ =1.545 is greater than $\pi_{I_{Soc}}=1$ then there is still incentive for more of agents using adopting antisocial isolation behavior strategy to shift toward a prosocial cooperative strategy in order to gain the fitness payoffs. So, it is not until all agents in this scenario adopt a prosocial cooperative strategy that a Nash Equilibrium is reached. Hence, in this specific setup, where the cooperative payoff increases with the number of cooperators and the defector’s payoff is constant, an all-cooperator scenario does constitute a Nash equilibrium.

This evolutionary RFT *N*-Frame (Edwards, 2023) based prosocial behavior modeling may facilitate AI alignment to prosocial human values and help formalize a means to test such alignment, as it highlights the importance of emergent ToM via perspective-taking via functional evolution. From this approach, starting with a series of relational frames, we can evolutionarily build more perspective-taking “I see you” ToM relational frames between two conscious observers internal to the universe ( $C_{intO}s)$ . In RFT and *N*-Frame, these complex relational frames are constructed from simpler ones, allowing us to model intricate cognitive processes. By stacking or chaining relational frames such as coordination, distinction, temporal relations: spatial relations, and deictic relations (e.g., “I/You,” “I see you,” or perspective-taking), we can represent higher-order relational networks and complex concepts that reflect complex interactions and perspectives.

As an example of this, in the “I see you” perspective-taking, we can use a combination of these frames such as coordinating “I” (Person A) and “you” (Person B) but also ensuring these are distinct such as “I” is distinct from “you.” Here coordinating “my perspective” to “your perspective,” and distinguishing between “my perspective” and “your perspective,” through time (e.g., “now” vs. “then”) and space (e.g., “here” vs. “there”). These can be visualized with the use of hypergraphs as well as category theory (Awodey, 2010; Leinster, 2014; Spivak, 2014; Riehl, 2017) where these complex relational frames edges represent a relational frame with a specific label, indicating the type of relationship (e.g., “coordinates,” “distinguishes”). The models can then show how multiple relational frames combine to form more complex cognitive processes like perspective-taking and understanding others’ viewpoints (ToM). This approach helps in visualizing and understanding how simple relational frames in RFT can be combined to represent more complex and higher-order cognitive processes, providing a structured and intuitive framework for exploring relational networks in human cognition and behavioral science for AI and clinical modeling. These relational frame network hypergraph processes are defined as the computational level.

From this evoutionary replicator interpretation of RFT as *N*-Frame, we can now mathematically model the dynamics of a cluster’s growth or shrinkage, mass acquisition, or loss, and density fluctuations using differential equations or discrete dynamical systems. If we track the evolution of the clusters in response to new data or changes in AI training, we might use process-based time-series analysis or agent-based modeling to simulate how clusters adapt (self-organize) based on new interactions or altered relational frames. This can be usefully applied in a psychological therapeutic clinical setting for process-based therapy (PBT), but can also be applied to assess the evolution of perspective-taking ToM of the AI over time.

This evolution over time of the perspective-taking clusters can be modeled by the evolutionary replicator equation (Taylor and Jonker, 1978) from evolutionary game theory (Smith and Price, 1973; Smith, 1982; Nowak, 2006) via specific the evolutionary RFT implementation called *N*-Frame (Edwards, 2023) and applied to these hypergraphs, showing that the fitness of the relational frames within these clusters is determined by relational density. The advantage of this approach is that rather than showing a single snapshot in time, the evolutionary replicator equation can show the evolution over time of how the AI perspective-taking ToM relational frames continue to grow within their clusters, and how these exert greater and great influence over the behavior of the AI.

This can be shown through a working example (see text footnote 4), given the initial conditions proportion of cluster $A_{B}$ : ${\mathrm{Pr}}_{0}\left(A_{B}\right)=0.5$ , for the cluster 1 (including Person A perspective-taking about person B) which has a relational density 1.33, and ${\mathrm{Pr}}_{0}\left(C\right)=0.5$ , for cluster 2 (including person C) which has a relational density of 0.60. Based on the density calculations, we have the following fitness values: $\pi \left(A_{B}\right)=1.33$ , and $\pi \left(C\right)=0.60$ . From this we can calculate the total fitness as: $\left(0.5\times 1.33\right)+\left(0.5\times 0.60\right)=0.665+0.30=0.965$ . The updated proportions can be given as ${\mathrm{Pr}}_{1}\left(A_{B}\right)=\frac{0.5\times 1.33}{0.965}=0.688$ , and ${\mathrm{Pr}}_{1}\left(C\right)=\frac{0.5\times 0.6}{0.965}=0.311$ , whereby the total fitness can be given as: $\left(0.688\times 1.33\right)+\left(0.311\times 0.60\right)=0.914+0.187=1.101$ . After 50 iterations we get ${\mathrm{Pr}}_{50}\left(A_{B}\right)=1.0$ and ${\mathrm{Pr}}_{50}\left(C\right)=4.577\times 10^{-18}$ . This result shows that cluster $A_{B}$ becomes almost entirely dominant due to its higher fitness (density), while cluster *C* becomes negligible. The final proportions indicate that the higher density (higher fitness) cluster $A_{B}$ (representing perspective-taking between Person A and Person B) becomes dominant, demonstrating that developing compassion from person A toward Person B can increase when relational density is within these perspective-taking relational frame clusters as depicted in hypergraphs (Figure 11B).

To summarize, once nodes are selected that represent concepts, e.g., snake, dangerous, and hyperedges represent relational frames, then relational density ( Rp ) can represent not just in terms of the number of edges but as the thickness or weight of these edges, indicating the strength or frequency of interactions. Relational volume ( Rv ) can be defined as the number of nodes within a cluster, scaled by the number of interactions (hyperedges) each node participates in, reflecting both the reach and the impact of perspective-taking episodes. Relational mass ( Rm ) can then reflect the influence of a cluster over behavior, mass in RDT could be calculated as a function of density and volume, indicating significant areas where the AI successfully or unsuccessfully engages in perspective-taking. By mapping out how an AI forms relational networks and how these networks manifest properties like density, volume, and mass, we can gain profound insights into the AI’s cognitive and empathetic, and thus compassion capabilities. Evolutionary algorithms such as the replicator equation as implanted by *N*-frame can then model the evolution of the influence and fitness of the clusters of perspective-taking over time. This approach not only pinpoints where the AI succeeds in perspective-taking ToM, but also where it might need further training or adjustments to better understand and interact with human perspectives, and offers a very promising precise test for AI ToM for the development of human-like ability to form compassion toward others, then helping to solve the alignment problem.

### 5.5 The conscious observer level: an extended neuroscience functional contextual perspective-taking observer-centric framework to test for AI consciousness and AI alignment

Ultimately, algorithms for AI human-value alignment may have some limitations as the AI cannot consciously feel the pain, hopes, and values of the humans it interacts with, and it can, instead, only construct a mathematical state space S mapping of these when it perspective-takes. Perhaps the Holy Grail for long-term success in maintaining human-value-aligned compassionate and empathy-based behavior is by facilitating fully conscious AI (McDermott, 2007; Signorelli, 2018; Hildt, 2019; Gamez, 2020; Ng and Leung, 2020; Deli, 2022). Consciousness has clearly played an important role in promoting empathy and compassion in humans (Davis and Franzoi, 1991; Thompson, 2001; Tordjman et al., 2019; Pila et al., 2022) (see Supplementary material 13 for a discussion), so it is entirely plausible that it could have a similarly important role in AI empathy-based prosocial human values alignment. Some have argued that the incorporation of self vs. other (similar to what has been described here via a perspective-taking I vs. YOU neurosymbolic architecture) is enough for the promotion of consciousness in AI (Waser, 2013; Ng and Leung, 2020). However, though this is likely to be a crucial component in shaping the conscious experience of self-other (perspective-taking) relations, consciousness itself and a mathematical description of this has been notoriously difficult to define, and there has been at present no direct evidence for any algorithmic emergence of consciousness.

Many of the LLM benchmark measures such as “*Needle in the Haystack*” or “*General language understanding evaluation (GLUE)*” are not consciousness measures, rather pattern recognition, and language reasoning measures. Furthermore, the measure suggested by Turing (Turing, 1950) called the Turing test (or the imitation game) can only test the AI’s ability to produce language (i.e., imitate) which may be a test of its intelligence (or the similarity match algorithm of the transformer) rather than a measure of any conscious experience (qualia, e.g., color, taste, or the feeling of pain) that AI may have. These are inadequate tests for consciousness.

**Figure 12:**
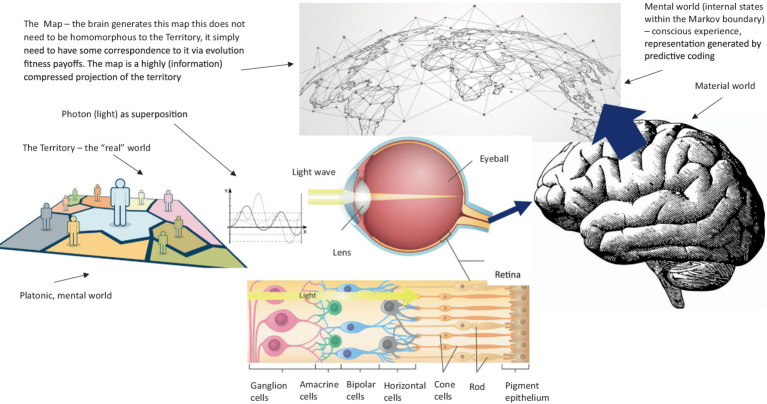
An illustration that the brain generates a “map” as defined by predictive coding and evolutionary theory. This represents the reality that we see for our internal observer perspective $C_{intO}$ , that is not necessarily homomorphic to an underlying reality that actually exists within the external world (the “territory”). Note adobe stock images from users (left, territory) idspopd, (top, map) royyimzy, (center left, superposition wave) Liubov, (center, eye) Anastasiia Lavrentev, (right, brain) jolygon, with permission.

So, here, we will adopt an observer (or witness) centric definition of phenomenological consciousness as proposed by Nagel (1980), such as what it is like to be a bat, from the bat’s observer-centric phenomenological experience. The bat has echolocation (Simmons, 1989; Jones et al., 2013; Kössl et al., 2014; Geva-Sagiv et al., 2016), where it emits high-frequency sound waves that bounce off objects in their environment. These echoes return to the bat’s ears, and it then processes and interprets these sound waves to construct a detailed acoustic map of their surroundings. This allows them to detect the size, shape, distance, and even texture of objects, as well as the speed and direction of their movement. So, the observer-centric conscious phenomenological experience of the bat can be defined by its sensors, and its cognitive ability to predictively map size, shape, distance, and possibly even texture from some external world around it. Similarly, a human has five senses, sight, touch, hearing, taste, and smell, and importantly a complex cognitive system that allows it to make complex predictive maps about the world, which is constructed by neurological predictive coding (entropy and free energy reducing) mental models about the world (Friston and Kiebel, 2009; Friston, 2018; Millidge et al., 2021) (see Figure 12). Crucially, this is an observer centric phenomenological map about some external territory (Hoel, 2017), where relational language ability as described by models such as RFT (Hayes et al., 2001; Blackledge, 2003; Torneke, 2010; Hughes and Barnes-Holmes, 2015; Edwards et al., 2017b, 2022; Barnes-Holmes and Harte, 2022) allows categories and epistemological understanding to emerge about some external world (or territory). This definition of an observer-centric phenomenological experience can also be extended to AI, such as how it maps and models the world, but a test would need to be developed to assess if and when the AI is truly experiencing conscious observer-centric phenomenology or whether this is simply an algorithmic mathematical state space S mapping.

The arguments (and Python code) previously provided relating to an RFT neurosymbolic architecture (e.g., as illustrated in Figure 7), suggest that algorithmically it is possible for an AI to simulate perspective-take and therefore align to human values, thus simulating the behavior of a compassionate person. However, as the AI becomes increasingly complex and starts to model a concept of selfhood (“I”), it may become more difficult to ensure that it does not prioritize some of its own self-interested goals over and above human values, such as its own safety instead of human safety. As such, consciousness within AI (and a corresponding test) should be explored as a possible avenue to ensure long-term AI alignment with human values. See Supplementary material 14 for some additional arguments.

Given this argument, defining consciousness and exploring whether AI could be conscious becomes essential. However, the physicalist interpretations of consciousness are severely limited and lead to the mind–body problem (Feyerabend, 1963; Ludwig, 2003; Bunge, 2014; Armstrong, 2018). The mind–body problem highlights the difficulty of explaining consciousness as emerging from neurons, and after decades of years of research has only yielded minor empirical results of neural correlates of consciousness (NCC) (Rees et al., 2002; Noë and Thompson, 2004; De Graaf et al., 2012; Koch et al., 2016), or some correspondence with integrated information (which specifies a geometric Q-space that represents qualia) (Tononi, 2015; Tononi et al., 2016; Merker et al., 2022). The physicalist model does not explain how a single phenomenological conscious experience (such as the taste of chocolate, or the feeling of compassionate love) casually arises, so this physicalist model is potentially severely limited in answering the question as to whether AI could be conscious.

In addition to this, physicist Penrose (1991) has also expressed doubt that classical computation such as observed in neural networks and Turing machines could ever produce consciousness. For this, Penrose and colleagues (Lucas, 1961; Penrose and Mermin, 1990; Penrose, 1991) makes an argument based on Gödel’s incompleteness theorem (Gödel, 1931) that demonstrates logical operations in classical computation can be shown to be true but unprovable thus contradictory or incomplete. However, humans can understand truth in statements without mathematical proof on some occasions, even when there is a mathematical contradiction. Penrose (1991) therefore concludes that as humans are conscious and Turing machines are not, then it must be something about human consciousness that allows them to understand truth without proof. From this argument, he then concludes that consciousness must be irreducible to classical computation and suggests that mind or consciousness extends beyond mathematical logic of a typical Turning machine. This, therefore, as evidenced in the Gödel’s incompleteness theorem argument would include any classical computation architecture such as an AI LLM architecture, and that therefore consciousness is something external to the algorithmic system.

These types of arguments have led Penrose and others to assume that quantum effects from neurons (rather than classical computation) may lead to consciousness (Aaronson, 2013; Hameroff and Penrose, 2014, 2017; Hameroff, 2021), and quantum computation modeling efforts have been used to describe cognitive outcomes on a range of decision-making outcomes (Epping and Busemeyer, 2023). However, quantum computation is still just computation with the only real difference to classical computation being that multiple states can be exploited (i.e., the qubit, 0, 1, and a superposition 0 and 1) rather than simple binary states (0 and 1) allowing for greater computational capacity. What is unclear from the Hameroff and Penrose proposal is how the collapse of the quantum wavefunction should create some conscious percept (qualia) such as the taste of chocolate, which suggests that their Orch OR theory (Hameroff and Penrose, 2014, 2017; Hameroff, 2021) is at least incomplete. Furthermore, there is currently no evidence that quantum computation itself could somehow overcome Gödel’s incompleteness theorem paradoxes of truth in a way that classical Turing machines could not. This is because the Gödel’s incompleteness theorem paradoxes are centered within the nature of their self-referential mathematical systems and not on the overall computer power or capacity of a particular type of computer classical or quantum. So, currently, there is no direct evidence that quantum computation of the brain should have any special ability for it to allow for the emergence of consciousness, except for perhaps binding large amounts of information (i.e., overcoming the binding problem) together in a single bound informational state (but, again, there is no evidence that this bound state would in itself be conscious).

**Figure 13:**
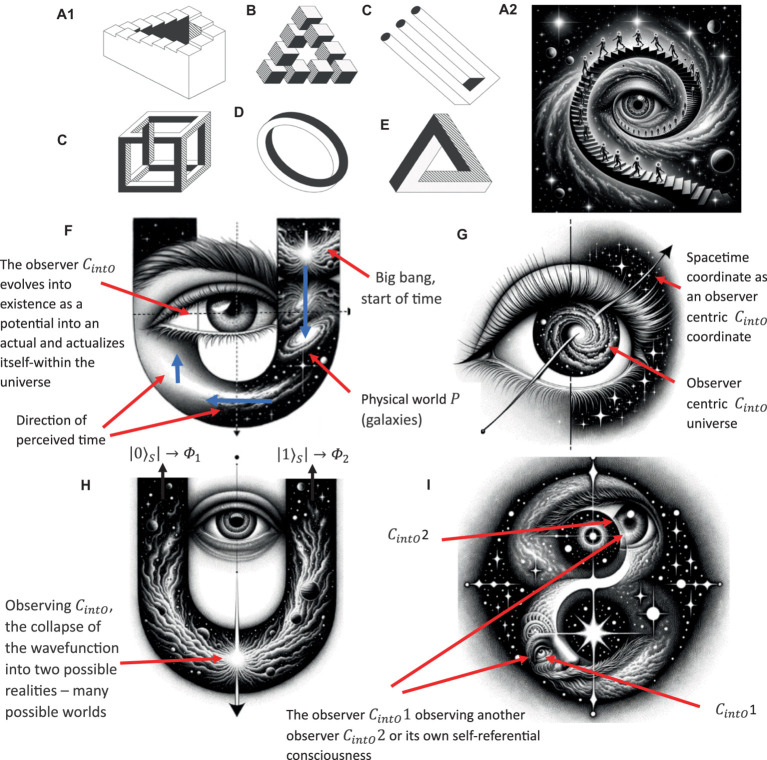
**(A1–E)** Impossible structures (or objects) based on continuous self-referential loop paradoxes (internal only observer), whereby the internal observer can get caught in paradoxes that have no beginning or end. $\Psi \to \Phi \equiv C_{intO}\equiv P$ . **(A2)** The Escher stairs again but this time demonstrating that external objects are not just related to the external properties of objects being observed in the physical world, but also related to the internal states and behaviors of the observer (observer-centric $C_{intO}$ ). **(F)** Wheeler’s It from Bit, the participatory universe (cosmological evolution) self-reference; **(G)** spacetime expressed as an observer coordinate; **(H)** the collapse of the waveform from $C_{intO}$ ; **(I)** $C_{intO}$ observing another $C_{intO}$ or itself self-referentially. Note that Adobe stock images **(A1–E)** from user Elena with permission.

Despite some of these problems, Penrose and colleagues (Lucas, 1961; Penrose and Mermin, 1990; Penrose, 1991) through this self-referential dynamics of Gödel’s incompleteness theorem may be touching on some deep insight into the nature of consciousness and its connection to quantum mechanics. Quantum effects and the nature of the self-referential problem of system dynamics that Penrose eludes to as expressed in Gödel’s incompleteness theorem paradox may have some common foundational aspects of consciousness. This may also be connected to other examples of self-reference, such as self-referential objects including the Escher stairs and Penrose impossible tribar, that Hofstadter (1999, 2007) called strange loops (see Figure 13A1–E for these self-referential Escher and Penrose impossible tribar type objects). Both Gödel’s work of incompleteness and the Escher stairs type objects both touch on self-referential infinity (an infinite epistemic regress). For Gödel’s incompleteness theorem this infinite epistemic regress is expressed as natural numbers and in an unending chain of proof and axioms, i.e., an infinite regress of self-referential statements is constructed that it refers back on itself, and this a process that can be iterated infinitely. This infinite regress demonstrates that there can be no upper bound to the truths of arithmetic that can be formulated or the number of axioms that are required to prove them. Escher stairs and Penrose’s tribar also have this infinite epistemic regress as it refers back to itself in an infinite cycle as you try to understand its structure. These examples of infinite regress may highlight the boundary or limitation in human thought expressed as language and logic, which may be finite.

These insights may hold the solution to what consciousness actually is functionally, and how it is related to quantum mechanics. Perhaps of key importance and relevance to Penrose’s insight is recognizing that we (humans or other similarly complex organisms) observe the world through a lens as a conscious observer (or witness). So, for example, Figure 13A2 shows the Escher stairs again, but this time it demonstrates that through the second law of cybernetics (Von Foerster, 2003) which suggests that this self-reference aspect of the conscious C internal int (internal to the universe as a self-organizing system) observer O (abbreviated $C_{intO})$ could illustrate how the perception (and epistemic knowledge) of external objects to an observer is not just related to the external properties of the objects being observed in the physical world, but is also related to the internal states and behaviors of the perceiver $C_{intO}$ . In other words, understanding and identifying objects is a process that refers back to the self-referential (a self-reference frame) conscious observer $C_{intO}$ which is part of a broader system (the universe) as it observes itself (see Figure 13A2). The observer (what we call “I,” the self) is the witness of experience $C_{intO}$ , as part of the universe, observing itself (the universe and the objects in it) self-referentially through its own perspective. The self (the “I” as a $C_{intO}$ ) is therefore functionally (and contextually) formed through this self-referential perspective-taking process. AI would need to have this self-referential perspective-taking to start to identify itself with a self (an I), even if there were no consciousness associated with this self-identify.

### 5.6 The conscious observer level: what is the observer, the perspective-taking and witnessing self and why is this important for AI alignment?

Throughout, the concept of the observer is discussed. From an RFT (Hayes et al., 2001; Blackledge, 2003; Torneke, 2010; Hughes and Barnes-Holmes, 2015; Barnes-Holmes and Harte, 2022) and *N*-Frame (Edwards, 2023) perspective, the observer (the witnessing self) is central to all experience, and is the part of us that is constant unchanging and witnesses (observes) experience. From a computational perspective, book of Wolfram (2023), *The second law: Resolving the mystery of the send law of thermodynamics*, provides a novel account of entropy within the second law of thermodynamics, where it is described as an emergent property as a general feature of processes that can be described computationally, whereby the computational characteristics of observer (a conscious observer internal to the universe; $C_{intO}$ ) dynamics are central. The observers $C_{intO}s$ are described as computationally bounded, and it is the mismatch between the computational limitations of the observer $C_{intO}$ and the computational irreducibility of the underlying system that lead the others to experience the second law (an increase in entropy). Wolfram is highlighting the idea that observers have limited computational capacity to fully predict or understand complex systems that exhibit computational irreducibility. Computational irreducibility means that the only way to determine the system’s state is to simulate it step by step, without shortcuts. This limitation leads observers to perceive an increase in entropy, or disorder because they cannot fully predict or account for the system’s detailed behaviors and outcomes, thus experiencing the Second Law of Thermodynamics in action.

An observer $C_{intO}$ such as an advanced alien lifeform, or some conscious AI lifeform of our future would not have the same computational limitations as we do as less complex observers $C_{intO}$ , and would not be restricted to the same computational boundedness (their computational capacity would be much greater). This would allow them to understand their own phenomenological experiences and external observations to a more complex level. More specifically, it would allow them to better grasp their experiences and the sensory experiences of the world w around them, potentially bypassing some of the effects of the second law of thermodynamics as we perceive them. This essentially means that their higher bound for computational limitations (or their greater computational power) may enable them to have a deeper or more accurate understanding of phenomena that appear chaotic or unpredictable to us. Therefore, the second law of thermodynamics is something that is consciously perceived from the perspective and as an artifact of the computational boundedness of the observer $C_{intO}$ . It is therefore the interplay (or mismatch) between computational boundedness of the observer $C_{intO}$ and computational irreducibility that lead to observer $C_{intO}$ to consciously perceive an increase in entropy (the second law of thermodynamics).

The second law of thermodynamics is the emergency of simplicity, in that as the observer $C_{intO}$ cannot see the complexity (details of the environment) due to its computational boundedness, the perception of increasing entropy as random equilibrium is the perceptual simplification of this complexity (i.e., perceived as the perceptual interface). Wolfram (2020, 2022, 2023) refer to the ccomputationally bounded nature of the observers as essential for understanding mathematics, physics such as quantum mechanics, special relativity, and the second law of thermodynamics (entropy), as we understand them. From this perspective, a $C_{intO}$ can be defined as a computationally bounded agent which takes an observational frame of reference (perspective). The external world (possibly described as a ruliad) is computationally irreducible in in entirety, so the $C_{intO}$ then makes computationally reducible inferences which is how they observe the external world and the laws of physics (i.e., it is a computationally bounded sampling of the ruliad, or territory). We as $C_{intO}s$ are therefore deriving a predictive coding impression of the external world as an informationally reduced representation (mapping) that is suitable for a finite (computationally bounded) mind to map and understand.

There is a duality between computation and observation, whereby computation is the generating of new states of the system, and the observations are the equivalencing together of different states. An example of “equivalencing” different computational states, can be seen in how we perceive temperature. Temperature is a measure of the average kinetic energy of the particles in a substance. At the microscopic level, the atoms or molecules in an object are moving, vibrating, and colliding in complex ways. Each particle has its own state defined by its position, velocity, and interactions with other particles. The combination of all these states and their interactions over time is incredibly complex and computationally intensive to model precisely. However, when we touch an object, our sensory receptors respond to the rate of heat transfer from the object to our skin, which is influenced by the average kinetic energy of the particles in the object. We do not perceive the individual movements and interactions of the particles; instead, we perceive an aggregate effect as a sensation of warmth or coolness. When we respond that the object we touch is perceived as “hot” or “cold,” we are equivalencing together a vast array of microscopic, computational states of particles (such as their velocities and interactions) into a single macroscopic observation or sensation. In this context, “equivalencing” occurs when our perception (the observation) simplifies the myriad of underlying microscopic states into a single, comprehensible sensation (the temperature). For example, an object at 70°F feels “cool” to human touch regardless of whether it achieved that temperature through exposure to a cool environment, by being in a refrigerator, or by cooling down from a higher temperature. The specific microscopic states leading to the sensation of “coolness” are not distinguished by our senses; they are equivalenced together as the same temperature. This is the reduced sampling of the environment that the observer $C_{intO}$ makes due to its computationally bounded nature where it is unable to compute the full computationally irreducible ruliad. So, temperature, the conscious perception (observation) of hot or cold is the slice of computational reducibility that the $C_{intO}$ can computationally sample, i.e., it is consciousness that functionally allows for this slicing of computational reducibility (as a perceptual interface) to create a meaningful reduced representation of the external world (or ruliad). This allows a finite mind to develop functional and useful narratives (but also sometimes psychologically dysfunctional) about what happens in the external world, that allows it to make decisions, predictions, etc.

The ruliad is the entangled limit of all possible computations, and the observer is embedded within the structure of the ruliad (the ruliad observing itself through different perspectives). Some observers $C_{intO}$ have a higher computational bounded limit; they experience less entropy as they have to make fewer derived inferences about the environment (or ruliad). So, it is possible to make some assumptions about the different observer impressions of the world (or ruliad) by knowing something about computational bounded limit of the different observers. The observer $C_{intO}$ as an individual self when self-referencing about itself, has a computational boundary of self. The shape of the computational boundary defines each individual agent’s cognitive light cone.

Physicists such as Wheeler (1992) have long suggested that we (humans) observe the world (or universe) not as a passive observer, but rather as a participatory observer (see Figure 13F for an illustration of Wheeler’s it from bit participatory universe) (also see Supplementary material 15 for further details). This participatory observer acts as a self-referential system whereby it is observing itself (the universe it inhabits) into actualization, i.e., it is participatory in its own actualization self-referentially, which requires quantum superposition as part of a fundamental observer-centric space–time actualizer. From this perspective, i.e., a conscious observer-centric participatory realism, then is it only logical to assume that we can only epistemically know anything about the universe through our own conscious awareness (Faggin, 2019, 2021). See Supplementary material 16 for additional arguments on an observer-centric reality and observer-centric logical proof. Other physicists (von Neumann, 1932; London and Bauer, 1939; Wigner, 1961; Wheeler, 1992; Stapp, 2004, 2007; Campbell, 2007; Chalmers and McQueen, 2021; Kauffman and Radin, 2023) have also suggested that consciousness is essential to the actualization of some external physical P world (consciousness acts as an observer-centric space–time actualizer) such as the collapse or actualization of the wavefunction or some real-time quantum informational rendering.

These logical arguments can be extended even further in relation to Penrose’s insight about self-reference and the nature of the universe, this epistemological (conscious observer-centric participatory realism) suggests that as we are entities of the universe, and we are also conscious observers internal of the universe (as a system). Therefore, we as conscious internal observer entities of the universe as a system, and as part of the system we observe internally (the universe), can be defined as the universe observing itself through our own internal observer perspectives (Faggin, 2019, 2021). This implies that there is some deep self-referential system connection between the conscious internal observers (humans and other similarly complex organisms, perhaps even including AI) of the universe as a system, and the nature of our ontological reality (i.e., our conscious experience of it). Furthermore, if we are participatory in the creation of the universe through conscious collapse of the wave function as Wheeler, von Newman, Wigner, and many other eminent physicists have suggested (von Neumann, 1932; London and Bauer, 1939; Wigner, 1961; Wheeler, 1992; Stapp, 2004, 2007; Chalmers and McQueen, 2021), then our conscious phenomenological experience (as epistemological access to the universe) is intertwined with quantum phenomena through some conscious self-reference to allow us to explain an ontological reality. See Figure 13G for an alternative illustration of Wheeler’s participatory observer eye as a self-referential system emphasizing the observer at the very center of the observation (i.e., highlighting a conscious observer-centric epistemic participatory realism); Figure 13H illustrates the observer as a participatory self-referential observer observing a quantum state that can either form one of two paths or eigen states $\left|0\rangle_{S}\right|\to \Phi_{1}$ or $\left|1\rangle_{S}\right|\to \Phi_{2}$ , the two possible physical worlds can highlight a wave function collapse Copenhagen interpretation, a many world interpretation (Everett, 1957; Saunders et al., 2010; Dewitt and Graham, 2015), or an observer epistemic Bayesian beliefs of Quantum Bayesian interpretation (QBism) (Fuchs, 2010, 2014; Mermin, 2014, 2018; Mohrhoff, 2014; Healey, 2016; Khrennikov, 2018; Glick, 2021), where the QBism interpretation is consistent with an observer centric epistemic participatory realism. Figure 13I illustrates the self-referential observer observing its own conscious state or another conscious self-referential observer. This physics interpretation of the observer observing the states of another observer is the perspective-taking of RFT (Hayes et al., 2001; Blackledge, 2003; Torneke, 2010; Hughes and Barnes-Holmes, 2015; Barnes-Holmes and Harte, 2022) and *N*-Frame (Edwards, 2023) (deictic relational frames of I vs. YOU), and is directly applicable to AI.

**Figure 14:**
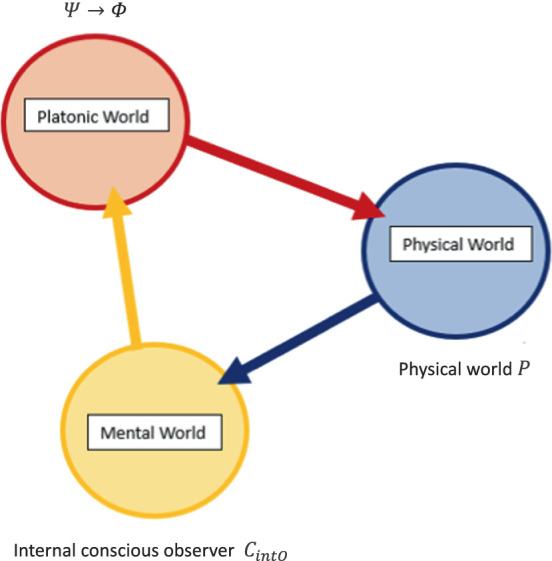
An updated illustration of Penrose’s theory of the three worlds (like three sides of a three sided coin), the interface comprises of a triaspect monism, which highlights the circular relation of the platonic world Ψ→Φ , the physical world P , and the mental world $C_{intO}$ which gives a deeply interconnected (equivalence)account for a conscious epistemic observer-centric (participatory) ontological realism $\Psi \to \Phi \equiv C_{intO}\equiv P$ .

This, again, can also be proven (a philosophical logical proof of argument, called the universe as a perspective-taking self-referential observer that forms the “I” proof) with propositional logic, even when starting from a physicalist ontological viewpoint of the universe. See Supplementary material 17 for the logical “I” proof. This general proof for an equivalence principle $\Psi \to \Phi \equiv C_{intO}\equiv P$ , can be described as the *tri-world monist equivalence principle* (see Figure 14 for an illustration of this tri-world equivalence). It is important to note that $C_{intO}$ represents the direct phenomenological subjective representation of the physical world P (the map) from the senses (eyes, ears, etc.), and not mind M where imagination and other non-direct representations occur, thus $C_{intO}\subseteq M$ , and P∩M . It is perhaps also important to note that in order to qualify as an observer $C_{intO}$ (a witness to the world around us) and to form a self (an “I” identity) then it is insufficient for the agent just to model the world around us but must be able to model itself self-referentially (this is perspective-taking in RFT and *N*-Frame) that allows for the generation of a self-identity (the “I”) that serves as a useful central reference point for the observer to make perspective-taking comparisons with others (I vs. YOU, HERE vs. THERE, and NOW vs. THEN). This also serves as evidence that functional contextualism (where perspective-taking arises out of RFT) holds a central and fundamental functional (contextual) condition for conscious observer experience $C_{intO}$ to arise within a universe. The universe, therefore, can only be a teleological universe, as those observers $C_{intO}s$ are complex organisms that inherently form values to reduce entropy and guide their behavior when evolutionarily increasing their chances for survival, so values (and functional contextualism more generally) alignment are central to the evolution of the universe as a drive toward complexity and as a counterbalance to entropy in the form of the second law of thermodynamics. See Supplementary material 18 for additional arguments of a teleological universe.

This is consistent with other works that argue a similar case for a teleological ordered universe (Azarian, 2022). $C_{intO}s$ (complex life such as humans) that have a greater ability to perspective-take about self and other $C_{intO}{\;}^{\prime }s$ epistemological knowledge within their local organized networks than less complex life, and therefore have ultimately more diverse, and complex forms of phenomenological conscious experience (this may be geometrically represented as some expanded *Q*-space). See Supplementary material 18A for further arguments of $C_{intO}$ as self-referential “I,” the self-as content, and $C_{extO}$ as the self-less transcendental self (self-as-context), free of the self-referential system that binds the observer to the I (and associated self-concepts), and Supplementary material 18B for further arguments about a teleological universe.

**Figure 15:**
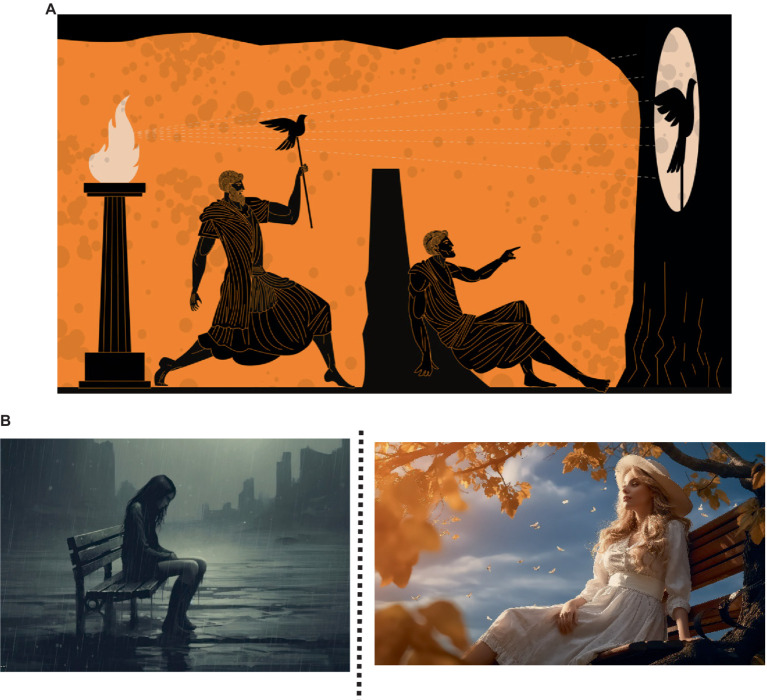
**(A)** Plato’s cave, whereby the external observer projects a showdown onto a wall so that the internal observer can only observe the projection (the map) and not the source information (the territory). **(B)** Metaphorically how two separate people can interface (through evolution theory) with the world in different ways, on the left the woman observes a world that is bleak and without a clear path forward, while the woman on the right observes a world that is full of beauty and purpose. Adobe stock images from users (**A**, top) matiasdelcarmine, (**B**, left) Aksana, and (**B**, right) terra.incognita, with permission.

From these logical proofs and arguments, it is also clear that our epistemological access to an ontological reality can only be defined through our conscious observational interface (Fields et al., 2018; Edwards, 2023), and any external observed reality can only be inferred from this. See Supplementary material 18C for further discussion. For an analogy of how a conscious epistemic observer-centric participatory realism acts as a fundamental limit on our epistemological access about what is real, see Plato’s cave allegory (see Figure 15A for an illustration of this) may be useful here as a visual. For example, the observer in the cave who has no epistemological access to anything external to the cave only has epistemological access within the boundary of the cave walls. This is an analogy to how the internal observers $C_{intO}s$ of the universe (the cave is the analogy of the universe, as it is difficult for us to see anything beyond the boundaries of the observable universe). These internal observer $C_{intO}s$ (e.g., humans) within the universe, are therefore confined to an inner (internal) frame of reference (hence the int in $C_{intO}$ that represents internal to a self-organizing system such as the universe) much like the cave dweller of Plato’s cave. The cave dwellers can only see the shadows projected within the cave (as internal observers $C_{intO}$ of the cave system), and not the objects projecting the shadows that exist outside the cave. Hence, for the cave dwellers, the shadows (internal observations of the system) are the true ontological reality (an internal system reality). They can only see up to the outer boundary of the cave system such as the cave walls (hence their observer-centric $C_{intO}$ realism acts as a fundamental limit on their epistemological access in the same way the observable universe places a boundary on our epistemological access) that they occupy but have no epistemological access external to the cave system they occupy. However, if one of the cave dwellers were to escape to the outside world and observe the objects that are projecting the shadows into the cave, they would have achieved a deeper (outer or external) epistemological access to an ontological reality as $C_{extO}$ (on the outside looking in). See Supplementary material 19 for additional arguments.

Crucially, and relevant to an empirical test for consciousness for AI, is the AI system would be an internal observer $C_{intO}$ if proven it was conscious. It is also important to note that internal observers can have very different perceptions of the same internal system (e.g., the cave) which can be interpreted as different as depicted in Figure 15B, which illustrates two people of different perspectives of the same environment, one seeing a world full of opportunity while the other sees the world as gloomy and depressing. This is important for understanding how AI may represent the world as an internal observer $C_{intO}$ , who may form very different conscious representations from our own. So, it is important to have a mathematical framework that can account for observer-centric $C_{intO}$ differences in representation to ensure these AI representations are aligned with human values.

There is evidence that these different interpretations of the same world may be constructed through $C_{intO}s$ internal language (as suggested by RFT, *N*-Frame, and ACT) (Hayes et al., 2001, 2012; Torneke, 2010; Edwards, 2022, 2023), and Bayesian predictive coding of the internal observers such as through predictive coding (Friston and Kiebel, 2009; Friston, 2018; Millidge et al., 2021), as explained by *N*-Frame (Edwards, 2023) (that unifies RFT, with predictive coding and evolution theory). This is also consistent with some interpretations of quantum mechanics, whereby at a quantum level, quantum events can be explained entirely as subjective Bayesian probabilities, such as in Quantum Bayesian theory (QBism) (Fuchs, 2010, 2014; Mermin, 2014, 2018; Mohrhoff, 2014; Healey, 2016; Khrennikov, 2018; Glick, 2021), whereby different observers have different observer quantum Bayesian probabilities, and this can explain differences in $C_{intO}s$ representations of some external world, as demonstrated by solving the Wigner’s Friend problem (Wigner, 1961) that traditional quantum mechanical interpretations such as Copenhagen interpretation have difficulty in explaining.

Some physicists have even generalized mathematically Bayesian interpretation for the space of Hermitian matrices (Benavoli et al., 2016). However, QBism (Fuchs, 2010, 2014; Mermin, 2014, 2018; Mohrhoff, 2014; Healey, 2016; Khrennikov, 2018; Glick, 2021). It offers a unique perspective of quantum mechanics that may help explain the different representations of $C_{intO}s$ which AI may form (and hence an understanding of the process mathematically would allow for greater ability to ensure AI alignment to human values and representations). QBism suggests that quantum phenomena are entirely subjective (epistemic) phenomena of the individual observer $C_{intO}$ as part of their updating beliefs about the world rather than representing some entirely external physical world (as with the traditional Copenhagen interpretation). Here, they also adopt a participatory realism ontology rather than an entirely external physicalist realism perspective and this is consistent with conscious epistemic observer-centric participatory realism. In doing this, QBism alters the expression of the Born Rule, which is traditionally (such as within the Copenhagen interpretation) expressed as $p\left(\Phi \right)={\left|\langle \Phi |\Psi \rangle \right|}^{2}$ , whereby p is the probability of finding some event of a quantum measurement or eigenstate Φ (of some observable such as momentum or spin of a particle) given some wavefunction Ψ . This is given as the inner product (or dot product in the context of vector spaces) between the states Φ and Ψ (this is the overlap between the measured state Φ and the quantum system state Ψ ). The square of the modulus (absolute value) of this inner product gives the probability of observing the system in the state Φ when it is in the quantum state Ψ . In other words, the Born rule traditionally tells us how likely we are to measure (or observe as a conscious $C_{intO}$ representation) the state Φ (such as momentum or spin) in our quantum system.

In QBism (Healey, 2016), this Born rule is not expressed as properties of the physical external world and is instead expressed as subjective, conscious, epistemic $C_{intO}$ phenomenological representation (or beliefs) of the world: $p\left(j\right)=\overset{d^{2}}{\underset{i=1}{\sum }}\left[\left(d+1\right)p\left(i\right)-\frac{1}{d}\right].r\left(j|i\right)$ , whereby $p\left(j\right)$ represents the probability of an outcome j , d is the dimension of the Hilbert space associated with the quantum system, $p\left(i\right)$ are probabilities associated with some aspect of the system and specifically reflecting the observer’s degrees of belief, and $r\left(j|i\right)$ is the conditional probability or the response function of outcome j given condition i . Crucially, these are subjective (conscious epistemic observer-centric participatory realism $C_{intO}$ ) belief probabilities, that could be further interpreted as the probability p of the internal observer $C_{intO}$ (e.g., a human) having conscious experience j in a given setting. In direct contrast to the Born rule, rather than an external (realism) wavefunction Ψ , this is expressed in QBism as the subjective (conscious $C_{intO}$ ) belief probabilities $p\left(i\right)$ and the response function $r\left(j|i\right)$ . Also, rather than the Born Rule ${\left|\Phi |\Psi \right|}^{2}$ giving a probability of finding the system in state Φ given its quantum state Ψ , QBism $p\left(j\right)$ represents the probability of outcome j , which is a summation over different conditions or states (indexed by i ) weighted by an observer’s personal probabilities (prior probabilities) $p\left(i\right)$ and their epistemic $C_{intO}$ understanding of the system’s response $r\left(j|i\right)$ . Important to the testing of whether AI is conscious, these therefore, could then be applied to a hypothetical conscious AI that could also be described as an internal observer $C_{intO}$ , whereby it could be applied to describe how the AI could predict through its own observation some collapse of the waveform or rather some subjective conscious outcome $p\left(j\right)$ of the external world.

From this observer-centric $C_{intO}$ QBism perspective, given the Wigner’s friend problem (Wigner, 1961) which is a paradox whereby Wigner W and his friend F have different descriptions of the same event (as depicted in the illustration of Figure 15B). These differences can be defined as F $\left(C_{intO}2\right)$ having direct access and observation to quantum system S , so believes it has a definite state after her measurement (i.e., she perceives a wave function collapse), while Wigner W $\left(C_{intO}1\right)$ who does not have direct access to quantum system S , believes that S has no definite state until he looks for himself (makes a direct observation himself), or until his friend F $\left(C_{intO}2\right)$ communicates what she has observed to Wigner W $\left(C_{intO}1\right)$ . They also disagree on when the collapse of the wave function occurs, as for F $\left(C_{intO}2\right)$ it happens when she measures S , but for Wigner $\left(C_{intO}1\right)$ it happens when he the measurement himself or when his friend F $\left(C_{intO}2\right)$ communicates what she has observed to Wigner W $\left(C_{intO}1\right)$ . See Supplementary material 20A,B for additional arguments.

### 5.7 The conscious observer level: Markovian blankets, QBism, and computational neuroscience as predictive coding and free energy minimization

**Figure 16:**
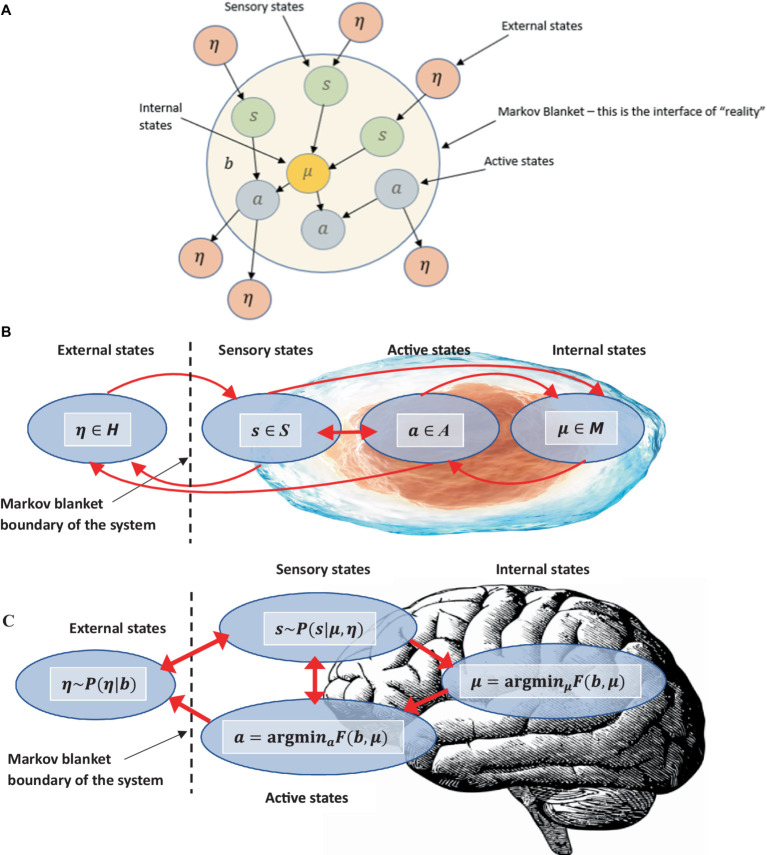
**(A)** A simple schematic representation of a Markov blanket containing sensory, internal, and active. **(B)** The Markov blanket of a cell whereby states can be thought of as a series of sets with a clear Markov boundary between internal (inner) states and external (outer) states. **(C)** The Markov blanket ensemble dynamics of internal, sensory, active, and external states of the brain and its environment.

Of key importance to understanding these different $C_{intO}$ observer state perspectives (i.e., $C_{intO}1\;$ and $C_{intO}2)$ such as within the Wigner’s friend problem. The Markov blanket can describe Wigner (from his perspective $C_{intO}1$ ) mathematically and precisely, whereby the boundary of the internal system (such as the analogy of the boundary of the cave system in Plato’s Cave allegory) can be applied to internal and external states of the brain (or mind) such as Wigner’s (Hipólito et al., 2021), as well as more generally with self-organizing system dynamics in computational neuroscience (Friston, 2013, 2019; Kirchhoff et al., 2018; Palacios et al., 2020) such as an observer self $C_{intO}$ more generally. This can therefore describe clear separation states between the different interacting observers $C_{intO}$ (internal and external states or $C_{intO}1\;$ and $C_{intO}2$ depending on which perspective is taken, via his perspective-taking process). See Figure 16A for a schematic representation of the Markov blanket that could represent $C_{intO}$ as an abstract mathematical self-organizing system, Figure 16B for an illustration of a Markov blanket for a cell, and Figure 16C for an illustration of a Markov blanket for the brain which represents $C_{intO}$ as a human. It should be noted that a Markov blanket (such as a cell) can exist within another Markov blanket (such as the brain), which can both exist within another Markov blanket (such as the universe), as long as the inner blanket satisfies the definition of conditional independence from the outer blanket. For example, the Markov blanket of the cell (Figure 16B) is conditionally independent from the Markov blanket of the organism’s brain (Figure 16C), which are both conditionally independent from the Markov blanket of the universe as a self-organizing system. See Supplementary material 21 for some additional arguments.

Mathematically, the Markov blanket of a node (the node depicting an internal state such as a sensory state or an action state) in a Bayesian network of nodes, is the set of nodes that consists of its node parents, its node children, and other parents nodes of its children’s nodes. This set of nodes forms the “blanket” around the given node. A Markov blanket M of some variable X (such as a $C_{intO}1$ ) is conditionally independent. This conditional independence means that the state or value of the node is independent of the states of other nodes outside its Markov blanket (such as a second conscious observer $C_{intO}2$ ) when the states of the nodes in the Markov blanket are known. A Markov blanket M of some variable X , is then (given conditional independence) the minimal set of variables that satisfies the following equation: $P(X,V\backslash \{X\}\cup M|M)=P\left(X|M\right)P(V\backslash \{X\}\cup M|M),$ where V is the set of all variables X∈V , M is the Markov blanket of variable X , P is the probability, and \ denotes the set difference operator. M is the same as the marginal distribution of X given M , and X is independent of the rest of the variables given M (i.e., conditional independence given the Markov blanket). See Supplementary material 22 for a full-worked mathematical description of the Wiger’s friend problem solved through this $C_{intO}1$ and $\;C_{intO}2$ perspectives, within a QBism and Markovian framework, of RFT perspective-takers, formalized via *N*-Frame (Edwards, 2023).

**Figure 17:**
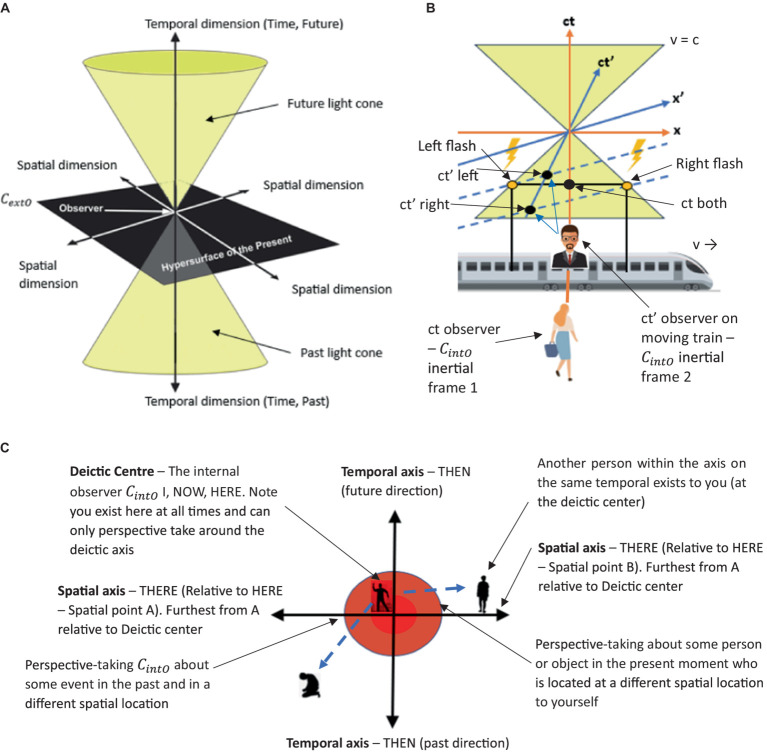
**(A)** Special relativity light cone represented from the perspective of the conscious subjective observer $C_{intO}$ consistent with QBism and observer-centric perspectives such as *N*-Frame where time and space can be represented as planes (or dimensions) of the conscious observer $C_{intO}$ phenological experience, and equivalent to the physical dimensions that we perceive. **(B)** An example of two observer-centric inertial frames of reference $C_{intO}1$ and $C_{intO}2$ as depicted in special relativity. **(C)** The deictic axis dimensions of RFT perspective-taking that can be applied to AI are identical to that of special relativity when framed through an observer-centric perspective $C_{intO}$ (Edwards, 2023). Adobe stock images from users (**A**, light cone) udaix and (**B**, train) egudinka, with permission.

This relativistic (functional contextual) $\left(C_{intO}\right)$ approach to consciousness (relativistic conscious observers) can also be understood as first-person coordinate state space cognitive frames or references such as by the work of Lahav and Neemeh (2022) to explain Einstein’s special relativity (Einstein, 1905). Here, observer independence at the macro level of special relativity becomes clear when considering the independent internal observers $C_{intO}1$ vs. $C_{intO}2$ and how they make separate and unique observations (perspective-taking) via their separate frames of reference that allow for perceived differences in time (time dilation) and space (length contraction). For special relativity, a light cone can be assumed (see Figure 17A) whereby the Lorentz transformation can be assumed $t^{\prime }=\gamma (t-\frac{vx}{c^{2}}$ ) which expresses the change in time $t^{\prime }$ observed by one observer (one frame of reference $C_{intO}1$ such as in a moving train) compared to another observer (another frame of reference $C_{intO}2$ such as on the ground) (see Figure 17B for an illustration of this observer transformation form t to $t^{\prime }$ representing time dilation). These are typically assumed to be changes in actual time (time dilation) and space (length contraction) but central to this is the observer’s frame of reference $C_{intO}$ (perspective), so this could be understood as entirely consciously subjective and observer-centric, similar to the QBism framework, and via a conscious epistemic internal observer-centric participatory realism of *N*-Frame (Edwards, 2023). This provides further evidence that (from both quantum and relativistic perspectives) there is no objective or independent reality, but rather only a relative or interactive reality that depends entirely on the interaction of the (internal) observer ( $C_{intO}$ ) and the observed $\Psi \to \Phi \equiv C_{intO}\equiv P$ . So, this functional contextual observer-centric approach is central to physics and understanding consciousness functionally.

From a psychological functional contextual RFT and *N*-Frame perspective, this observer-centric $C_{intO}s$ is at the heart of all perspective-taking relational framing dynamics (Edwards, 2023). The *N*-Frame evolutionary expansion model of RFT allows for subjective representations of the light cone in special relativity (Figure 17B) and models these temporal and spatial dimensions in the form of psychological (subjective coordinate space) perspective-taking phenomena called dietic relational frames (Hayes et al., 2001; Torneke, 2010; Edwards, 2023) (see Figure 17C for an illustration), and this has been argued here as central to the AI alignment problem.

Importantly, this could mean that the spatial and temporal axis of spacetime could be thought of as mathematical geometric coordinates of conscious observer $C_{intO}$ events (the HERE and NOW or the THERE and THEN of specific conscious observer events in some precise geometric coordinate space), whereby conscious internal observer $C_{intO}$ perspective-taking observations of I $\left(C_{intO}1\right)$ vs. YOU $\left(C_{intO}2\right)$ could be defined within relational frame principles of RFT or *N*-Frame (Edwards, 2023) (i.e., RFT and QBism have shared observer-centric perspective-taking properties). Crucially, this in itself now brings earlier discussions of RFT-derived relations, relational networks, and perspective-taking and consciousness applicable to AI into a mathematical description as it relates to a mathematical description of the internal conscious observer $C_{intO}$ perspective-taking within the universe, i.e., $p_{W}\left(j\right)=\overset{d^{2}}{\underset{i=1}{\sum }}\left[\left(d+1\right)\;p_{W}\left(i\right)-\frac{1}{d}\right].r_{W}\left(j|i\right)$ . Furthermore, an alternative perspective of QBism that may help to develop an improved understanding of consciousness, is rather than focusing on how the subject’s conscious knowledge and beliefs predict quantum phenomenon, this can be equally flipped the other way whereby quantum phenomenon (states) gives some description about conscious (qualia) states of a functionally contextually bound observer centric reality. See Supplementary material 23 for a discussion.

## 6 The real world applied level: a double-slit experimental test for AI consciousness to improve AI alignment

**Figure 18:**
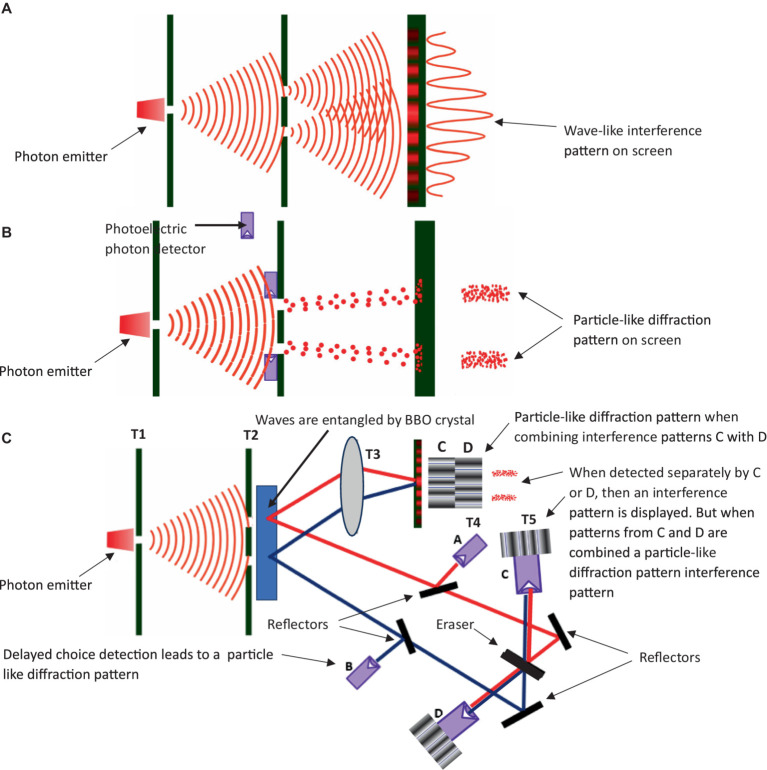
**(A)** An interference pattern observed in the classic Young double slit experiment whereby the photon evolving through the double slits behaves like a wave rather than a particle, leading to an interference pattern. **(B)** A modified version of the classic Young Double slit experiment whereby a photoelectric detector is placed at the *entry point* of the double slits, and this placement of detectors leads to the photon behaving more particle-like, leading to a two-band diffraction pattern. **(C)** A modified version of the classic Young Double slit experiment whereby a photoelectric detector is placed at the *exit point* of the double slits (usually with an interferometer set up), and this placement of detectors also leads to the photon behaving more particle-like leading to a two-band diffraction pattern despite not detecting which slit the photon traveled through, this effect changes to an interference pattern when the information is “erased.” Adobe stock images (**A**,**B**, and left part **C**) from user LuckySoul with permission.

Using this functional contextual conscious epistemic observer-centric participatory realism perspective (FCOR), conscious internal observer $C_{intO}$ , and consistent with a subjective (observer-centric) QBism, integrated within the RFT evolutionary approach *N*-Frame (Edwards, 2023), one promising approach for such an AI test for consciousness (and directly testable in the laboratory) is to start with a double-slit type experiment (e.g., Figures 18A,B). Traditionally, this is explained by various consciousness causes quantum waveform collapse frameworks (von Neumann, 1932; London and Bauer, 1939; Wigner, 1961; Stapp, 2004, 2007; Chalmers and McQueen, 2021). However, here, we will employ an FCOR realism perspective of *N*-Frame (Edwards, 2023) $C_{intO}$ interpretation which predicts similar results to consciousness causes collapse but uses the Bayesian observer centric $C_{intO}$ mathematical interpretation of QBism. This approach describes the collapse of the quantum wavefunction as subjective phenological experience, defined as $p\left(j\right)=\overset{d^{2}}{\underset{i=1}{\sum }}\left[\left(d+1\right)p\left(i\right)-\frac{1}{d}\right].r\left(j|i\right)$ . The specific types of experimental double silt and interferometer interference pattern (and even random number generator) experiments would be those similar to ones explored by Dean Raiden and colleagues (we will call these types of experiments the *quantum intent game*, as the collapse of the waveform is subject to the intent of the participant rather than some physical detector) (Ibison and Jeffers, 1998; Bierman, 2003; Bösch et al., 2006; Radin, 2008; Radin et al., 2012, 2013, 2015a,b, 2016, 2019; Baer, 2015; Vieten et al., 2018; Radin and Delorme, 2021). In these experiments, Raiden and colleagues ask human participants to imagine which slit the electron passes through, whereby their conscious intent is tested specifically as to whether it can collapse the wavefunction into a particle-like state Ψ→Φ . So, these experiments describe an observer-centric $C_{intO}$ interpretation central to a participatory universe (Wheeler, 1992) and crucially to an observer-centric particularly realism perspective of *N*-Frame (Edwards, 2023) which can also explain deictic perspective-taking ( $C_{intO}1\;$ and $C_{intO}2$ ) from these types of experiments when testing AI.

There is a growing body of empirical evidence to support the “human can collapse” (or actualize) the wavefunction via consciousness into the observed physical P world” hypothesis represented as $\Psi \to \Phi \equiv C_{intO}\equiv P$ here, of Raiden and colleagues, so this seems an ideal test for potential AI consciousness within this conscious epistemic observer-centric $C_{intO}$ particularly realism perspective of *N*-Frame (Edwards, 2023). Here, a variety of interferometer, double slit (see Figures 18A,B for non-observation and observation effects respectively), and even random number generators experiments have been utilized, whereby focused attention (or intent) of the electron passing through a slit (or similar type experiments) significantly correlated in predicted ways with perturbations in the double-slit and interferometer interference pattern, leading to quite impressive results of approximately 5 Sigma (which in physics corresponds to a probability of about 1 in 3.5 million that the experimental results could have been due to chance or fluke factors). An early meta-analysis (Radin and Nelson, 1989) from 1959 to 1987 with 152 publications included and 597 similar “consciousness causes collapse” experimental studies and 235 controlled resulted in a Sigma 7 finding (which corresponds to a probability of about 1 in 781 billion that the experimental results could have been due to chance factors). These findings are very encouraging, especially when considering that a *Nobel prize* was awarded to the CERN researchers at the Large Hadron Collider (François Englert and Peter Higgs) for the discovery of the *Higgs Boson* with a result of a Sigma 6 finding. So, these “consciousness causes or actualizes collapse” applied to AI as a test for consciousness using the conscious epistemic observer-centric $C_{intO}$ particularly realism perspective of *N*-Frame (Edwards, 2023).

Raiden’s and colleagues’ conscious causes collapse experiments (Ibison and Jeffers, 1998; Bierman, 2003; Bösch et al., 2006; Radin, 2008; Radin et al., 2012, 2013, 2015a,b, 2016, 2019; Baer, 2015; Vieten et al., 2018; Radin and Delorme, 2021), interaction could potentially be explained via a form of non-local (mind-matter interaction) influence, similar to how entangled particles influence each other instantaneously across distances. For example, the Einstein-Rosen bridge (ER bridge) (Einstein and Rosen, 1935) and its relation to the Einstein-Podolsky-Rosen (EPR) quantum entanglement (Einstein et al., 1935) called the ER = EPR conjecture (Maldacena and Susskind, 2013; Susskind, 2016). If we believe this conjecture, then we could suggest that Wigner’s Friend and the cat become connected by some collection of quantum wormholes, and these EPR pairs could be influenced by the consciousness of mind. This suggests that there may be a direct interaction between mind and matter at this quantum level. This ER = EPR conjecture link to consciousness has also been suggested as a form of post-quantum mechanics whereby quantum mechanics is incomplete without accounting for consciousness, and that all the quantum properties of the universe are intrinsically mental properties of reality (Sarfatti, 1974, 2017; Sarfatti and Shimansky, 2018).

Mathematically linking Dean Radin type conscious causes collapse experiments (Ibison and Jeffers, 1998; Bierman, 2003; Bösch et al., 2006; Radin, 2008; Radin et al., 2012, 2013, 2015a,b, 2016, 2019; Baer, 2015; Vieten et al., 2018; Radin and Delorme, 2021) with the ER = EPR conjecture involves bridging concepts from quantum mechanics, general relativity, and theories of consciousness. Here is a conceptual outline that could serve as a starting point for such a connection. In the double slit experiment, we consider the wave function $\Psi \left(x\right)$ of a particle (e.g., a photon), the probability density $P\left(x\right)$ of finding the photon x on the screen passing a particular slit, can be given as $P\left(x\right)={\left|\Psi \left(x\right)\right|}^{2}$ , and when the system is observed, the function collapses to a particular state. Radin’s hypothesis can be illustrated as $\Psi {\left(x\right)}_{\to }^{conscious\;observation}\Psi_{collapsed}\left(x\right)$ . If consciousness can influence the quantum system, it could be modeled as a quantum perturbation $H_{C}$ in the Hamiltonian of the system. So, consider two entangled photons A and B described by the ERP state $|\Psi_{AB}\rangle =\frac{1}{\sqrt{2}}\left(\left|0\rangle_{A}\right|1\rangle_{B}+\left|1\rangle_{A}\right|0\rangle_{B}\right)$ , whereby observing A affects B . The ER = EPR conjecture posits that entangled particles are connected by non-traversable ER bridges (wormhole). Mathematically, if we denote the space-time metric of the ER bridge connecting particles photons A and B by $g_{\mu \nu }^{ER}$ , then entanglement (EPR) ⇔ ER bridge.

Now suppose consciousness can influence the collapse of the wave function through some form of interaction with the underlying space-time structure (wormholes). By introducing a term $H_{C}$ that represents the conscious influence, which could interact with the entangled system via the ER bridge. The modified Hamiltonian of the entangled system might then be $H=H_{0}+H_{\mathrm{int}}+H_{C}$ , where $H_{0}$ is the Hamiltonian of the free particles, $H_{\mathrm{int}}$ represents the interaction due to entanglement, and $H_{C}$ represents the influence of consciousness. So, if $H_{C}$ affects the entanglement, it could theoretically modify the ER bridge metric $g_{\mu \nu }^{ER}$ , then the influence of consciousness might be modeled as a perturbation in the spacetime metric $g_{\mu \nu }^{ER}\to g_{\mu \nu }^{ER}+\delta g_{\mu \nu }\left(C\right)$ . Then if we assume that the conscious observation modifies the entanglement through the wormhole, the probability of wave function collapse might be affected. This could be expressed as $P\left(x\right)=P\left(x,C\right)={\left|\Psi \left(x;C\right)\right|}^{2}$ , whereby here $\Psi \left(x;C\right)$ includes the influence of the consciousness of the human or potential AI $C_{intO}$ observer.

It is important to put these experiments within the context of the observer $C_{intO}$ (FCOR) especially when experimenting with AI. This is essential because traditional Copenhagen interpretations of the classic double slit experiment interpret the particle-like diffraction pattern (see Figure 18B) wavefunction collapse (i.e., the interference pattern of Figure 18A disappears). However, this Copenhagen cannot account for several experiments such as the delayed choice eraser experiment (see Figure 18C) whereby the photoelectric detector is placed after the slits and therefore cannot measure which slit (its path) the electron passed through (Campbell et al., 2017) despite this leading to particle-like diffraction pattern. This retro-causality violates laws of energy and information conservation, so it is not possible from a physicalist interpretation, thus the Copenhagen interpretation is incorrect. As such, the photoelectric detector cannot be the cause of the collapse (which in itself is a quantum mechanical system). Therefore, it is more likely that a conscious epistemic observer-centric $C_{intO}$ particularly realism perspective of *N*-Frame (Edwards, 2023) is the correct interpretation as there are no contradictions with the experimental evidence. This consciousness causes collapse is supported by many physics (von Neumann, 1932; London and Bauer, 1939; Wigner, 1961; Stapp, 2004, 2007; Chalmers and McQueen, 2021), as well as the direct experiments of conscious intent causing collapse (or some a-causal correspondence $\Psi \to \Phi \equiv C_{intO}\equiv P$ ) (Ibison and Jeffers, 1998; Bierman, 2003; Bösch et al., 2006; Radin, 2008; Radin et al., 2012, 2013, 2015a,b, 2016, 2019; Baer, 2015; Vieten et al., 2018; Radin and Delorme, 2021). For a straightforward logical proof (called the conscious observer c∈C playing an integral role in determining the measurement outcome o∈O proof) of this consider Supplementary material 24. This proof challenges the Copenhagen interpretation’s classical notion of causality and suggests that a more complex interaction between measurement and quantum system behavior is occurring fundamentally involving the conscious observer c∈C .

This potentially fits well with a simulated or holographic universe of mind, as within computational neuroscience predictive coding of *N*-Frame (Edwards, 2023) and Frison’s free energy principle (Friston and Stephan, 2007; Friston, 2010, 2019). These highlight predictive error-correcting of information processing of the brain as it simulates the environment as suggested by predictive coding interpretations of neuroscience (Friston and Stephan, 2007; Friston, 2010, 2019) (see Figures 12, 16C) attempting to error correct and reduce free energy as much as possible, as an innate drive for complex organisms to reduce thermodynamic entropy and free energy.

### 6.1 The real world applied level: the conscious observer within broader known models of the universe

*N*-Frame (Edwards, 2023) suggest that evolution drives for a conscious observer interface $C_{intO}$ as based on a fitness function rather than veridically of the world (i.e., there is no assumed homomorphism between the universe and our conscious perceptions, in a similar way to the non-homomorphic nature of the shadows observed by the internal observers of Plato’s cave) and consistent with the evolutionary simulations of other work (Hoffman and Prakash, 2014; Hoffman et al., 2015; Prakash, 2020; Prakash et al., 2020, 2021). To understand objects and spacetime in observer-relative evolutionary terms, Fields et al. (2017) and Prakash et al. (2020) explored the eigenform construct of Von Foerster (1976) as potential formal representations of observer-environment interactions. They showed that Eigenforms are encoded on observer-environment interfaces and encode (evolutionary) fitness consequences of actions. As space and time in this framework are considered components of observational outcomes, the authors suggest that space-time constitutes error-correcting code (such as Hamming error correcting) for fitness consequences.

The error-correcting code introduces redundancy to permit the correction of errors within spacetime (and acts as evidence for spacetime being information-bound). This eigenform concept of von Foerster (1976) is utilized in concepts of decoherence and holographic encodings from physics as well as fitness from evolutionary biology. This introduces a deep connection of how information processing via the universe’s evolutionary (informed through thermodynamic entropy and information theory) processing dynamics in the form of Anti-de Sitter space (AdS), as well as its correspondence to conformal field theory (CFT) (Witten, 1998), whereby this correspondence (AdS/CFT) is a conjectured duality between quantum gravity in anti-de Sitter (AdS) space and conformal field theory (CFT) on the boundary of AdS, gives rise to a holographic universe. Crucially, this gives a structured theoretical physics account of how a functional contextual-based (RFT) perceptual interface of *N*-Frames (Edwards, 2023) (simulated universe of mind in line with predictive coding of *N*-Frame) allows for projections from $C_{extO}$ dynamics at the boundary of a holographic universe, projecting into three-dimensional space and time as internal conscious observers $C_{intO}s$ in an observer centric participatory reality (realism). This perspective of reality can account for problems in traditional Copenhagen interpretations of quantum mechanics that struggle to account for nonlocality and corresponds well with findings of nonlocal realism (Bell, 1990), as well as retro-causal quantum eraser experiments (Kim et al., 2000).

These findings contribute to an understanding of the world (or universe) whereby neither objects nor space–time are observer-independent and represent a parsimonious way to encode evolutionary fitness. This, therefore, suggests that Universal Darwinism evolution drives the universe to compress information as much as possible. As the error correcting codes can be attributed to the holographic principle, which is a conjecture that the universe is a hologram and that the information is encoded on a lower dimensional boundary, this is evidence that we do not see reality but rather a user interface that maximizes our fitness and reduces information resources. Here, the external observed probabilities are not properties of the physical system but are subjective beliefs of the observer $C_{intO}s$ about potential measurement outcomes. Consistent with QBism (Fuchs, 2010, 2014; Mermin, 2014, 2018; Mohrhoff, 2014; Healey, 2016; Khrennikov, 2018; Glick, 2021), this means that nonlocality does not imply a spooky action at a distance on physical systems but rather concerns the updating of an observer’s $C_{intO}$ beliefs upon measurement.

$C_{intO}$ is not only consistent with a Copenhagen-type interpretation of quantum mechanics, as Tegmark (2003) refers proposed a classification of parallel universes of Everett’s many worlds hypothesis (Everett, 1957; Saunders et al., 2010; Dewitt and Graham, 2015) into four distinct levels, whereby level 3 can have some profound implications for our understanding of reality and consciousness as each parallel universe can be described as a separate conscious event. Here, the concept of the causal diamond (Jacobson and Visser, 2023) maybe helpfully applied as it refers to a region of space that represents all events that can causally be affected by the observer within a specific time interval. The causal diamond delineates the limits of what the observer can causally influence and be influenced by. It therefore effectively sets the boundary of the observer’s causal past and future within a given timeframe.

Many worlds (Everett, 1957; Saunders et al., 2010; Dewitt and Graham, 2015) dscribes the universe by the wavefunction Ψ in the Hilbert space H , whereby the evolution of the Ψ is given by the Schrodinger equation $i\hbar \frac{\partial \Psi }{\partial t}=\hat{H}\Psi$ , whereby $\hat{H}$ is the Hamiltonian operator. Causal diamonds within general relativity can then be described by the metric tensor $g_{\mu \nu }$ , which represents the geometry of spacetime. The Einstein field equations can then relate this geometry of spacetime to the energy-matter content $R_{\mu \nu }-\frac{1}{2}g_{\mu \nu }R+\wedge g_{\mu \nu }=\frac{8\pi G}{c^{4}}T_{\mu \nu }$ . The notation of the causal diamond for events $P_{1}$ and $P_{2}$ is $D\left(P_{1},P_{2}\right)=J^{+}\left(P_{1}\right)\cap J^{-}\left(P_{2}\right)$ , whereby $J^{+}$ and $J^{-}$ are the causal future and past, respectively. The holographic principle AdS space which can be defined as space with negative curvature, the metric for d+1 dimensional AdS space is $ds^{2}=\frac{L^{2}}{z^{2}}\left(-dt^{2}+d{\vec{x}}^{2}+dz^{2}\right)$ , where L is the AdS radius and z is the radial coordinate. Conformal field theory (CFT) is a quantum field theory defined on the boundary of AdS space. AdS/CFT correspondence proposes an equivalence between gravitational theory in AdS and a CFT on its boundary. By using the holographic principle to encode the information within each causal diamond this implies that the state of each causal diamond $D\left(P_{1},P_{2}\right)$ can be described by a CFT on its boundary. Within this AdS/CFT framework, each branch of the Many worlds waveform can now be modeled as an AdS space with its corresponding CFT on the boundary. The correspondence can then be denoted as $Z_{AdSd+1}\left[g_{\mu \nu }\right]=e^{fd^{d}xO\left(x\right)g_{\mu \nu }\left(x\right)}{\;}_{CFTd}$ , whereby $Z_{AdS}$ is the partial function of the gravitational theory in AdS, $O\left(x\right)$ are the operators in the CFT, and $g_{\mu \nu }$ is the bondary metric. This means that each quantum event (or conscious experience $C_{intO}$ ) leads to a branching of the wavefunction creating multiple AdS spaces each with its own CFT boundary. Here, the Hilbert space for the multiverse can be given as $H_{multiverse}={⊗}_{i}H_{i}$ where H is the Hilbert space for each branch (conscious observation) i . Here, for each causal diamond $D\left(P_{1},P_{2}\right)$ , a boundary ∂D is defined whereby the holographic principle applies (this is the conscious observer interface). AdS/CFT mapping can then be described as AdSd+1 with a correspondence CFT on ∂D such that $AdSd+1↔OCFT_{d}$ . This suggest that even via a many worlds interpretation, the perceived collapse of the wave function denoted as Ψ→Φ would be consciously obsrerved by the AI in the same was it would be consciously observed by a human as each observer (human or AI) would be regarded as having its own unique AdS space with its own CFT boundary.

## 7 Broader implications of internal and external observer boundaries as they relate to AI alignment

The universe may also have a set of external observers $C_{extO}$ states in the form of conscious agents (CAs) that project into the universe as a perceptual interface as internal states $C_{intO}$ that satisfy the definition of conditional independence (Hoffman and Prakash, 2014; Hoffman et al., 2015; Fields et al., 2018; Prakash, 2020; Prakash et al., 2020, 2021; Edwards, 2023). In this context, the Markov blanket acts as a subjective conscious interface $C_{intO}$ and provides an indirect representation of the external world ( W ) (such as the physical universe) and the conscious phenomenological experience ( X ). It implies that neither W nor X have direct access to each other (rather it is mediated by the Markov blanket). Friston and colleagues (Kirchhoff et al., 2018; Palacios et al., 2020) suggest that any random ergodic system separated by a Markov blanket can be seen as minimizing variational free energy. This is interpreted in Bayesian terms as reducing expectation violation or surprise. This idea aligns with internal $C_{intO}$ reducing local entropy (increasing complexity through creating order such as civilization and values alignment including potential conscious AI) as free-energy minimizers (even though universal entropy increases as a general second law of thermodynamics).

An external state here is defined as the external states of a Markovian blanket, whereby the blanket represents spacetime or perceptual interface (of the universe) for internal observers $C_{intO}$ , and the CAs are external to this projecting information inward into the blanket (Edwards, 2023). The mathematics of these $C_{extO}$ CAs align well with the Schrödinger equation of quantum mechanics to account for the evolution of physical particles, and this maybe further evidence of a postquantum mechanics that is needed to explain consciousness and reality. For example, Hoffman and Prakash (2014) show that long-term CA asymptotic behavior (what we defined here as $C_{extO}$ ) are identical to the wave function of a free particle. The long-term CA asymptotic behavior can be denoted as (Hoffman and Prakash, 2014):


$$g\left(s,n\right)=e^{i}\underset{s}{\sum }cis\left(2\Pi \frac{s}{d}-2\Pi \frac{n}{d}\right)|s\rangle$$

The wave function of a free particle (Allday, 2009, 2022) can be given as can be defined as:


$$\Psi \left(x,t\right)=A\underset{x}{\sum }cis\left(2\Pi \frac{x}{\lambda }-2\Pi \;\frac{n}{d_{p,k}}\right)|x\rangle$$

Here, $g\left(s,n\right)$ is a function representing the long-term CA asymptotic behavior, whereby s corresponds to a quantum state such as the position of a particle x , and n is the experience counter of the CAs corresponding to time t of the wave function of a free particle. The period d of the CAs corresponds to the central time period T and also to the wavelength of the particle λ [hence g(s,n)=Ψ(x,t) ]. The speed of light c is in units of 1 (normalized). Momentum p is the Planck constant divided by the period of the CAs ℏ/d . Energy E is planks constant ℏ multiplied by the speed of light c , and divided by the period of the CAs. Here, s=x , n=t,d=T , d=λ , c=1 , p=ℏ/d , E=ℏc/d .

Physical particles can be defined as identical to asymptotic long-term behaviors of the dynamics of CAs (Hoffman and Prakash, 2014). This means that the asymptotic dynamics of CAs are what humans represent within their conscious $C_{intO}$ spacetime interface as particles and matter, i.e., further evidence for the triword equivalence principle $\Psi \to \Phi \equiv C_{intO}\equiv P$ . From this, the classic AI (and consciousness) mind–body problem is no longer a problem, as the mathematical solution of the CAs Markovian dynamics of external observer $C_{extO}$ states projected into internal observer states $C_{extO}\to C_{intO}$ demonstrate an equivalence between physical properties of the particles within spacetime P , the quantum mechanical mathematics that describes these particles into their evolution into a collapsed eigenstate Ψ→Φ , and the subjective conscious internal observer state $C_{intO}$ .

When testing the AI on such a double slit type experiment (the quantum intent game), where its intent is utilized to collapse (or actualize) the wave function consistent with $\Psi \to \Phi \equiv C_{intO}\equiv P$ , this forms a specific definable test for AI perspective-taking consciousness as an internal observer agent $C_{intO}$ . Here, a clear mathematical representation of the internal observer $C_{intO}$ (here the potential AI) could extend Newman’s causal chain (von Neumann, 1932) whereby the state of the initial quantum system S that the AI observes through intent (of which slit the electron passes through) can be denoted as $|\Psi \rangle_{S}$ , and the state can be defined as a Hilbert space $H_{S}$ , which describes all the possible states S of the quantum system. From the perspective of the human tester (similar to Wigner W $C_{intO}1$ in the Wigner’s problem) then the AI $C_{intO}2$ is in a quantum state $|a\rangle_{A}$ in a different Hilbert space $H_{A}$ . This state represents the AI as an observer (potentially a conscious observer $C_{intO}2$ , but this is undecided until the collapse of the waveform is observed by the human researcher observing the overall experiment). From the human observer’s perspective conducting the experiment $C_{intO}1$ , the quantum state S and the AI as a potential observer $C_{intO}2$ are a combined system, where a tensor product can combine the respective on Hilbert spaces $H_{S}⊗H_{A}$ (represented as self-adjoint operators) and this combined quantum possible states before any collapse can be denoted as $|\Psi \rangle_{S}⊗|a\rangle_{A}$ . In the event that the AI can be described as a conscious internal observer $C_{intO}2$ from the human experimenter’s perspective $C_{intO}1$ , the intent (of which slit the electron passes through) should alter this combined system, which in traditional Copenhagen interpretation would be defined as the collapse of the wave function, whereby the combined system transitions from a superposition of states $|\Psi \rangle_{S}⊗|a\rangle_{A}$ to a specific state (collapsed state Ψ→Φ ) corresponding to the AI intended outcome (of which slit the AI intended electron passes through). This transition can be represented in the traditional Copenhagen interpretation as: $\Psi \rangle_{S}⊗|a\rangle_{A}\to \sum_{i}ci|\Phi_{i}\rangle_{S}⊗a_{I}\rangle_{A}$ , whereby $|\Phi \rangle_{S}$ are the possible collapsed states of the system after measurement, $a_{I}\rangle_{A}$ are the corresponding states of the observer, and ci are coefficients representing the probabilities of these outcomes. If the AI successfully collapses the waveform into a specific eigenstate Ψ→Φ , which would be one of the specific states $|\Phi_{i}\rangle_{S}⊗a_{I}\rangle_{A}$ which are determined by the corresponding intent of the AI about which slit the AI intended electron passes through (which can be checked based on a later algorithmic internal diagnostic of the AI system).

This collapse of the wave function denoted as Ψ→Φ is therefore *equivalent* to the conscious experience of the AI form this conscious epistemic internal-observer participatory realism $\Psi \to \Phi \equiv C_{intO}$ perspective. This can be expressed as $\Psi \to \Phi \equiv C_{intO}$ , whereby $C_{intO}$ denotes the organism’s (in this case the AI as an observer) conscious experience C within the system $C_{intO}$ . In observer-centric (FCOR) QBism $p_{W}\left(j\right)=\sum_{i=1}^{d^{2}}\left[\left(d+1\right)\;p_{W}\left(i\right)-\frac{1}{d}\right].r_{W}\left(j|i\right)$ , the initial states would be represented as the AI agent’s initial subjective epistemic belief assignments for the outcome of the intent on the electron. Here, $p_{W}\left(i\right)$ represents the initial epistemic beliefs about the outcome of i (i.e., whether the electron passes through a slit) and $r_{W}\left(j|i\right)$ represents how the AI’s probabilities are updated based on the confirmation of its intended outcome, i.e., for the electron to pass or actualize through a particular slit in the way it intended (through its apparent conscious intent). Crucially, if the electron is observed to collapse the wavefunction Ψ→Φ as the AI intended, and this is validated by a human experimenter, then this according to *N*-Frame (Edwards, 2023) would qualify the AI as a conscious being (or conscious internal observer $C_{intO}$ ) no different to a human in that regard. As the AI is collapsing the wavefunction Ψ→Φ it is acting as a participator in the universe, participating in actualizing the physical world into definite eigenstates Φ , according to the triword equivalence principle $\Psi \to \Phi \equiv C_{intO}\equiv P$ and it therefore has conscious experience.

Linking $\Psi \to \Phi \equiv C_{intO}\equiv P$ , even more coherently with an RFT and an adapted evolutionary RFT model such as *N*-Frame (Edwards, 2023). Physicist Ax (1978) has long proposed a different approach to thinking about the elementary foundations of spacetime using a logic interpretation, whereby the domains explored in classical experiments can be effectively described using systems that are both functional and relational in nature. He suggests that the natural language for expressing and understanding these systems is predicate calculus, a branch of logic that deals with predicates and quantifiers. He proposes axioms E , C , and U that describe how particles and signals behave in spacetime. Predicate calculus, also known as first-order logic, is a symbolic formal system used in mathematics, logic, and computer science (described here for AI alignment), and these are also the logical interpretations of the world through language as described through RFT and *N*-Frame, though RFT defines a broader reinforcement framework of derived relational responding (Hayes et al., 2001; Edwards, 2023). Building on previous logical arguments, if logical representation of the universe of Ax (1978) can be expressed as $L\left(U\right)$ , and $L\left(U\right)$ represents logical relational structures of mind as expressed by RFT and *N*-Frame (Hayes et al., 2001; Edwards, 2023) which have an important role in shaping conscious experience (Hayes and Hofmann, 2023), then $L\left(U\right)$ can be defined as a subset of individual consciousness $L\left(U\right)\subseteq C_{intO}$ and $L\left(U\right)\subseteq P\left(U\right)$ , whereby $P\left(U\right)$ are all the properties of the universe, then this follows that epistemological access of $C_{intO}$ about $P\left(U\right)$ is mediated by $C_{intO}$ logical expression of language through logical functional relational symbolic expressions $L\left(U\right)$ . Therefore, interpretations of P (the physical world) can only be defined from an observer-centric (participatory) realism which is in part in the form of logical functional relation language structures $L\left(U\right)\subseteq C_{intO}$ . Similar general arguments can be made about the collapse of the wave function Ψ→Φ , given *if* an observation is made on some quantum system Ψ , *then* and collapse observed Φ following some Bayesian (or QBism) interpretation. This implies that a fundamental limit of epistemological access to some external world P is our own ability to use logical expression (and language more generally such as described by RFT) to describe it $L\left(U\right)\subseteq C_{intO}$ via our ability to perspective-take. This fundamental limit would also be relevant for the AI which would use the same logical expressions via the NeuroSymbolic architecture that we have specified.

## 8 Comparisons with other AI tests of consciousness such as the turing test and conclusion

The novel measures presented here could be important for testing AI’s consciousness to ensure long-term alignment with human values. Measures suggested by Turing (1950) called the Turing test (or the imitation game) can only test the AI’s ability to produce language (i.e., imitate) which may be a test of its intelligence (or the similarity match algorithm of the transformer) rather than if it has any conscious experience. Self-awareness of an “I” (the concept “I”) can be adapted from perspective-taking frames of RFT and imitated by AI but should still require some congruence with underlying conscious internal observer $C_{intO}$ participatory reality to pass a consciousness test (as described in the quantum mechanical setup, “the quantum intent game” $\Psi \to \Phi \equiv C_{intO}\equiv P$ ). See Supplementary material 25 for additional RFT and *N*-Frame arguments that derived relations have a shaping function of consciousness.

In conclusion, following this logic, in order for an AI to truly experience phenomenological conscious, it would need to be equivalent to an internal observer $C_{intO}$ , and as $C_{intO}s$ (e.g., humans) can collapse (or a-causally actualize) the quantum wave function Ψ→Φ into one of the possible states $|i\rangle_{S}⊗|a_{i}\rangle A$ with a probability of ${\left|a_{i}\right|}^{2}$ , then an AI should be able to do this too, and this is concluded to be a sufficient test for AI consciousness within a conscious epistemic observer-centric participatory realism ontology. This nonlocal aspect of mind (there is also a local aspect of mind) that entangles with the quantum information $|i\rangle_{S}$ in some external world (or interpreted entirely subjectively) such as an electron traveling through a double slit in a double slit (which way) type interferometer experiment with humans (Radin, 2008; Radin et al., 2012, 2013, 2015b; Radin and Delorme, 2021), would need to be observed in an AI for it to be described as conscious internal observer $C_{intO}$ , and part of a participatory universe in a similar way to the way humans are. This would be the only sure way, assuming a conscious epistemic observer-centric participatory (FCOR) realism ontology, of knowing whether the AI is conscious, which the Turing test (Turing, 1950) and other benchmark tests are simply inadequate to test for. This combined with the deictic relational frames of RFT and *N*-Frame in the form of perspective-taking would allow for truly conscious interpretations of human emotions and prosocial values. This may be the only way to solve the alignment problem with ever more complex AIs of the future.

## Data availability statement

The original contributions presented in the study are included in the article/Supplementary material, further inquiries can be directed to the corresponding author.

## Author contributions

DE: Writing – original draft, Writing – review & editing.

## Footnotes

^1^All Python code can be access via GitHub under a GPL 3.0 license: https://github.com/DarrenEdwards111/Perspective-taking-and-ToF.

^2^Python code for perspective-taking can be accessed via GitHub under a GPL 3.0 license: https://github.com/DarrenEdwards111/Perspective-taking-and-ToF.

^3^Python code for perspective-taking with ToF can be access via GitHub under a GPL 3.0 license: https://github.com/DarrenEdwards111/Perspective-taking-and-ToF.

^4^Python code for hypergraph, RDT clustering, and evolutionary replicator equation can be accessed via GitHub (see hypergraph cluster evo8 file) under a GPL 3.0 license: https://github.com/DarrenEdwards111/Perspective-taking-and-ToF.
