# Supplementary material for: A functional contextual, observer-centric, quantum mechanical, and neuro-symbolic approach to solving the alignment problem of artificial general intelligence: safe AI through intersecting computational psychological neuroscience and LLM architecture for emergent theory of mind
Source: Front Comput Neurosci. 2024 Aug 8;18:1395901. doi: 10.3389/fncom.2024.1395901 (PMC11338881; doi:10.3389/fncom.2024.1395901)
Supplement: Supplementary file 1 [file Data_Sheet_1.PDF]

Supplementary Material: Neuro-symbolic natural language processing

Supplementary 1 – Encoder vs. Decoder LLMs

The original transformer model (Vaswani et al., 2017) was comprised of both an encoded and decoder for the purposes of language translation, i.e., encoding the text from a source (e.g., English) language and a decoder for the purposes of decoding the text to produce the desired output text in the target language (e.g., French). The encoder was designed to take an input sequence and apply a series of self-attention and feed-forward layers to produce a hidden representation of it (on the hidden feed-forward layers). The decoder then takes the hidden representation and generates an output sequence through the use of further self-attention and feed-forward layers, as well as an additional attention layer that attends to the encoder output. The final output is then passed through a SoftMax layer to produce a probability distribution over the possible tokens.

Supplementary 2 – Self-attention similarity

Self-attention computes the similarity between a query vector and a set of key vectors and then uses the scores to weight the corresponding value vectors. The weighted sum of the value vectors is then returned as the output. Specifically, the query, key, and value vectors are derived from the input or output embeddings by using different linear projections. The self-attention scores A^h are computed as follows which uses the dot product operation between Q^h and K^h in the formula for self-attention scores A^h: $A^h = softmax\left(\frac{Q^h {K^h}^T + M}{\sqrt{d}}\right)$, whereby d is the dimension of the queries and keys, and M is a mask (for masked self-attention) to prevent the model from attending to the future tokens. The dot product is a matrix of size $n \times m$, denoted as $Q^h \cdot {K^h}^T = Q^h \times {K^h}^T$ where ${K^h}^T$ is the transpose of K^h (its rows and columns are swapped around). It **is** used to measure the similarity between each query vector

and each key vector, which reflects how much each word should attend to each other word in the input sequence. The dot product is scaled by dividing by the square root of d, which is done to prevent large values from dominating the SoftMax function. M is usually a lower triangular matrix, where the lower triangle is filled with zeros and the upper triangle is filled with negative infinity. This is called masked self-attention because the model can only attend to itself and not to the future tokens that it has not generated yet, and this ensures that the model can only attend to the current and previous tokens in the output sequence, but not the future ones. The output of the attention mechanism for each head is then $O^h = A^h V^h$. The value vectors V^h are used to obtain a weighted sum of the input embeddings based on the attention scores A^h, which gives the output of each head O^h. The outputs from all heads are concatenated and linearly transformed to give the final output of the multi-head attention mechanism.

Supplementary 3 – Acceptance and commitment therapy concepts that could facilitate values-based orientation in AI.

ACT specifies six key processes that promote psychological flexibility and value orientation, which in humans are important concepts in the promotion of wellbeing, and life satisfaction as well as promoting a cooperative prosocial society (Atkins & Parker, 2012; Atkins et al., 2019; Edwards, 2022a; Kashdan & Rottenberg, 2010; Lucas & Moore, 2020), and which could be appliable to AI values alignment (solving the alignment problem). Central to a functional contextual ACT is the identification of and commitment to meaningful values (Edwards, 2022a; Hayes et al., 2006; Hayes et al., 1999, 2011). Values act as life directions that guide behavior toward what is meaningful. They are absolutely chosen in the present moment and can be flexible and context-dependent. Working towards values comes in the form of completing specific goals that are measurable outcomes that are guided by values.

Values are therefore culturally, personally, and socially relativistic as they are context-dependent, and there is no single set of values that is universally true for everyone in all situations (though they need to be absolutely and concretely defined for each individual). Cultural background, education, and social-economic status can all pay a role in forming our values in the society we live (Edwards, 2022a; Hayes et al., 2012). Individuals can freely choose their values based on what's important to them and what works for them in their current contextual situation (Edwards, 2022a; Hayes et al., 2006; Hayes et al., 1999, 2011). Values are not judged by how they conform to some external criterion, but instead by how they help one to live a meaningful and fulling life (Edwards, 2022a; Hayes et al., 2012).

The focus of values from this psychological (ACT) clinical perspective may have some useful perspectives within the context of the AI alignment problem, as it can potentially be usefully applied within a discussion of how to explore and define explicitly the human values from which the AI should learn from, and the values that AI should itself develop. Furthermore, at a more basic level of functional contextualism, through the lens of RFT, deictic perspective-taking properties which explain self as content and self as context within ACT's middle-level approach, may help to explain how prosocial behavior, empathy, and compassion within humans emerges (Atkins & Parker, 2012; Atkins et al., 2019; Edwards, 2022a; Neff & Tirch, 2013), and this could potentially be usefully applied in explaining how empathy and compassion can emerge within the information theory perspective of AI. For example, dietetics, and perspective-taking could be directly programmable within a neuro-symbolic module of an LLM, which could expand on simpler forms of self-reference prompts used in AI currently (Fernando et al., 2023).

Supplementary 4 – Worked example of a functional contextual utility function

As a simple example of this, consider some value such as building mindfulness skills, and some actional goals such as practicing mindful meditation. Using an expected utility approach, the first step would be to determine the possible outcomes which may include the following: outcome one, a significant increase in mindfulness skills; outcome two, a moderate increase in mindfulness skills; outcome three, no change in mindfulness skills; outcome four, a reduction in mindfulness skills. A second step would then be to assign a perceived (or predicted) utility value to each outcome, for example, a significant increase in mindfulness skills = +10; a moderate increase in mindfulness skills = +5; no change in mindfulness skills = 0; a reduction in mindfulness skills = -2. A third step would be then to assign a perceived (or predicted) probability of occurrence to each outcome based on the occurrence of the behavioral action being carried out, for example: probability of a significant increase in mindfulness skills = 0.5; probability of a moderate increase in mindfulness skills =0.3; probability of no change in mindfulness skills = 0.1; probability of a reduction in mindfulness skills = 0.1. A forth step is then to calculate the expected utility in the following way: $EU = (U\ of\ outcome\ 1\ \times P\ of\ outcome\ 1) + (U\ of\ outcome\ 2 \times P\ of\ outcome\ 2) + \cdots$, therefore the expected utility of practicing mindful meditation can be calculated as follows: $EU = (10 \times 0.5) + (5 \times 0.3) + (0 \times 0.1) + (-2 \times 0.1) = 5 + 1.5 - 0.2 = 6.3$. Multiple other behaviors can then be calculated in a similar way, and the behavior that leads to a higher EU would be chosen. Again, in this functional contextual version of EU, only behaviors that lead to purposeful valued based action have a high likelihood of high utility (such as wanting to develop mindfulness skills) and not trivial behavior (such as having a snack). Another function $f(v)$ could then be specified about what mathematically constitutes a value v.

Supplementary 5 – Worked example of functional contextual utility function, whereby $U = f(a, w, s, t, i)$, and $Con = w, s, t, i$

In an example of how context can affect EU, when trying to increase mindfulness skills, if the world w is noisy, then this distracting environment may lower the EU (compared to a quiet environment with fewer distractions) of attempting to practice mindfulness at that time. So, practicing mindfulness meditation to improve mindfulness skills may have a lower probability of success in a noisy environment when compared to a quite tranquil environment. So, this can be calculated in a similar way as previously, whereby the probability of a significant increase in mindfulness skills = 0.2; the probability of a moderate increase in mindfulness skills = 0.5; the probability of no change in mindfulness skills = 0.2; probability of a reduction in mindfulness skills = 0.1. The expected utility can then be calculated in the same way as before: $EU = (10 \times 0.2) + (5 \times 0.5) + (0 \times 0.2) + (-2 \times 0.1) = 2 + 2.5 - 0.2 = 4.3$. When comparing the noisy environment with a value of 4.3 to the value of the previous example (a quite environment) 6.3, the quite environment clearly has a higher EU, and emphasizes why modeling context is so important. This could be applied to other contextual factors such as current state s, such as fatigue, higher levels of fatigue or stress may lead to a lower probability of a significant increase in valued action of developing their mindfulness skills. Further empirical data would need to be collected to inform the exact nature of the function f that describes how these contextual factors relate to one another, or the magnitude that can affect the individual. For example, a linear relationship could be assumed: $U = j \cdot a + k \cdot w + l \cdot s + m \cdot t + n \cdot i$, whereby j, k, l, m and n are coefficients that determine the weight (or importance) of each factor in the utility (function contextual) calculation, and how they specifically impact the individual (the higher the coefficient, the higher the weight and impact the specific contextual factor has on the

individual's EU). The function could also be nonlinear, interactive, and more entirely complex, but empirical data would be required to specify this under different circumstances.

Supplementary 6 – Framing effects of irrational behavior through prospect theory

It should also be noted that EU only models rational behavior and irrational behavior could also be modeled for risk aversion, such as described by prospect theory (Kahneman et al., 1982; Kahneman & Tversky, 1979, 2013; Tversky & Kahneman, 1974), which could be factored into individual differences i as an additional parameter. Framing effect $V = \sum_{i=1}^{n} \pi(p_i)v(x_i)$, whereby V is the overall expected utility of the outcomes of some decisions (or prospects) x_1, x_2, …, x_n some individual makes, p_1, p_2,…, p_n are the respective outcome probabilities of occurrence for these decisions. π is a probability weighting function which captures a cognitive bias found in these types of studies. This specifically reflects that individuals tend to overreact to small probability events and underreact to larger probability events. v is a function that assigns a value to the outcome of a decision, which determines how painful or satisfying a decision will be. The value function in prospect theory is S-shaped, and this passes through the reference point. This theory assumes that individuals are irrational as losses have been found to be more painful than gains feel satisfying, which means individuals are typically loss aversive.

Supplementary 7 – Areas of LLMs derived relational limitations

An additional study (Wu et al., 2023) explored whether LLMs such as ChatGPT-4 were actually able to reason or were simply reciting text they had been directly trained on. The authors used counterfactual variants of a default task, whereby solving the counterfactual variants of the task required the LLM to derive from the assumptions underlying the default task. Here, they explored different ways to ask the LLM the same question that use the same

underlying logic but were asked in a different way with different facts presented. They found that LLMs such as ChatGPT-4 could perform these counterfactual variants, but with substantially degraded performance when compared to the default conditions. This suggests that the LLMs had limited ability to derive the underlying information (assumptions) of the default task, and thus limited reasoning skills. This supports the study by Berglund at al. (Berglund et al., 2023), in that LLMs have limited ability to derive complex relations from their knowledge-base that can be usefully applied to logical reasoning tasks.

The limits of LLMs compositionality have also been explored (Dziri et al., 2023), whereby researchers have tested the limited of LLMs on compositional tasks, which are tasks that involve generating coherent and contextually relevant output based on given input, and sometimes a chain of reasoning (step by step reasoning) such as multi-digit multiplication, logic grid puzzles (e.g., the Einstein puzzle), and a classic dynamic programming problem. These types of tasks require contextual reasoning (logical) breaking problems down into sub-steps and synthesizing these steps to create a precise answer. Their findings suggest that transformer-based LLMs solve compositional tasks by reducing the multi-step compositional reasoning components of these tasks into linearized subgraph matching, without necessarily developing systematic chain-of-reasoning problem-solving skills. They found that LLMs perform well with tasks with low compositional complexity, but performance drops drastically with tasks with high compositional complexity. The authors concluded that LLMs map patterns from their training data rather than developing problem-solving skills, and that systematic problem-solving skills do not emerge from the transformer-based maximum likelihood training approach on input-output sequences. This chain-of-thought reasoning can be improved when the individual steps of the reasoning are reinforced by human feedback each time they occur (Lightman et al., 2023), i.e., each reasoning step is verified by a human step by step. However, even then, these LLMs still produce many errors and tend to invert

facts in moments of uncertainty (Bubeck et al., 2023). This ability to derive relations maybe dependent on the quality of the data the LLM is trained on, i.e., whether the training data includes examples of dynamic contextual frames, which it can learn from as well as the size of the network.

Supplementary 8 – RFT implementation of Chomsky hierarchy

This is a formal language theory in linguistics that has been applied to AI, and that provides a containment hierarchy of a formal grammar. Given a language's syntax, a formal grammar can describe how to form strings of words in a sentence from a language's vocabulary. Chomsky suggested that four different classes of formal grammar exist, with each one being able to produce different levels of complexity in language generation. These include Type 3 Regular Grammar, expressed as $\alpha \rightarrow a\beta$, where $\alpha \in N$, and $a \in \Sigma$ and $\beta \in N \cup \{\epsilon\}$; Here, α is a single non-terminal from the set N (a non-terminal symbols are placeholders for patterns of terminal symbols that can be substituted in a production rule. They represent abstract syntactic categories or classes of phrases, like noun phrases, verb phrases, or expressions in programming languages), a represents a terminal from the set Σ (terminal symbols are the actual characters or symbols of the language defined by the grammar), and β represents a non-terminal or the empty string from the set $(N \cup \{\Sigma\})^*$. Type 2 Context-free grammar, denoted as $\alpha \rightarrow \beta$, $\alpha \in N$, $\beta \in (N \cup \{\Sigma\})^*$; Type 1 Context-sensitive grammar whereby the length of α must be less or equal to the length of β. This is expressed as $\alpha \rightarrow \beta$ where $\alpha \in (N \cup \Sigma)^+$, and $\beta \in (N \cup \Sigma)^+$ and $|\alpha| \leq |\beta|$; and Type 0 Unrestricted grammar, with no restriction on production rules, and expressed as $\alpha \rightarrow \beta$ where $\alpha \in (N \cup \Sigma)^+$, and $\beta \in (N \cup \Sigma)^*$, whereby α is a symbol representing the string of nonterminal and terminal symbols on the left-hand side of the production rule. Symbol β represents a string of nonterminal and terminal symbols on the right-hand side of the

production rule. The transformation form α to β in a production rule is represented by $\rightarrow N$ and is a set of nonterminal symbols that represent grammatical categories that can be further expanded. Σ is a set of terminal symbols that are the actual symbols of the given language, such as words (or tokens), that cannot be expanded further. The following expression $(N \cup \Sigma)^{+}$ denotes one or more (indicated by the plus sign) symbols from the union of nonterminal symbols (N is the set of non-terminal symbols) and the terminal symbols (Σ is the set of terminal symbols). In this case, α must contain at least one symbol from N or Σ. The following expression $(N \cup \Sigma)^{*}$ denotes zero or more (indicated by the asterisk) symbols from the union of nonterminal symbols of set N and the terminal symbols of set Σ. This means that β can contain any number of symbols from N or Σ, including the possibility of an empty string.

Given that RFT models the relation between stimuli and how context affects throes relations, there may be some analogy between context-sensitive grammar and RFT's approach to context sensitivity between stimuli, whereby RFT can expand upon Chomsky's hierarchy (Chomsky, 1956). Here, in a situation where language describes relationships between stimuli (objects), the type of relationship depends on the context indicator. In this instance, nonterminal symbols include, S (sentence), R (relation), O (object), and C (context), and a set of terminal symbols can be used to represent objects (stimuli) and relationships. Objects (or stimuli) could include "apple", "banana", and "cherry", and relations could include "equals", "opposes" and "compares to". For context indicators C, there could be two contexts C_1 and C_2. The production rules could be expressed as follows: $S \rightarrow C\ R$, where $C \rightarrow C_1 \mid C_2$, and $R \rightarrow O$ equals $O \mid O$ opposes $O \mid O\ compares\ to\ O$, and $O \rightarrow apple \mid banna \mid cherry$.

This context-sensitive RFT grammar can then be defined as a tuple $G = (N, \Sigma, P, S)$. Here, N is a set of nonterminal symbols: $N = \{S, C, R, O\}$, Σ is the set of terminal symbols

$\Sigma = \{C_1,\ C_2,\ apple,\ bannana,\ cherry,\ equal,\ oposite,\ compares\ to\}$. P is a set of production rules, defined by specific logical relations, and S is the start symbol. The production rules can then be represented as logical statements, defining when certain strings can be replaced by others. We define P as a set of tuples, where each tuple represents a production rule, such that:

$$P = \{(S, C, R)\},$$

$$(C, C_1), (C,\ C_2)$$

$$(R, O\ equals\ O), (R, O\ opposes\ O), (R, O\ compares\ to\ O),$$

$$(O, apple), (O, bananna), (O, cherry),$$

$$(C_1\ O\ equals\ O, C_1\ apple\ equals\ bananna),$$

$$(C_1, O\ opposes\ O, C_1\ apple\ opposes\ cherry),$$

$$(C_2, O\ compares\ to\ O,\ C_2\ apple\ compares\ to\ bannana)$$

This, therefore, provides a strong type one context-sensitive grammar, uses a mathematical representation through the use of sets to define the components of the grammar, and logical relationships to define the production rules. It provides a formal way to describe the structure of the grammar using the principles of set theory and first-order logic. It has the ability to handle context in its production rules, making them more able to complex relationships and dependencies, making them more suitable for modeling phenomena such as perspective-taking which is important for developing compassion in AI and thus helps solving the alignment problem.

Supplementary 9 – additional commentary about the Python-derived relational code

The code itself has additional details such as iterating over the stimuli and relations, checking if relations exist, comparing strings, and calling the function recursively. This derived relation code is akin to imperative programming a series of steps are described to

achieve the result of stating (printing) directly learned and indirectly derived relations. However, the way the "$derive\_relation$" relational code is stated in terms of set theory and first-order (predicate) logic has elements of declarative or symbolic programming in that it describes the logic of the computation (i.e., what the relational code is supposed to do), without specifying the exact control flow. Importantly, the tuples allow the relational code to work with order-dependent relations, such as "greater than" or "less than". The function itself is commutative because it can handle both cases of (a, b) and (b, a) by returning the correct relation based on the order of the stimuli within the tuple (as the tuple is order dependent). The function therefore depends on the order of the stimuli in the tuples.

Supplementary 10 – Incorporation of the functional contextual architecture into the LLM

OpenAI (De Angelis et al., 2023; Markov et al., 2023) currently enforce ethical behavior of the LLM through three procedures: (1) Content moderation endpoint, whereby text generated by ChatGPT complies with OpenAI's usage policy; (2) safety setting, whereby OpenAI introduced settings to control the levels of risk and creativity of ChatGPT's output; (3) OpenAI also employs human reviewers who monitor the quality and safety of ChatGPT's output, who then provide feedback on how to improve its performance and behavior. There are several possible ways to implement such an architecture. However, these current ethical protocols have been shown to be bypassed in serval ways such as by certain "jail break" prompts, which prompt the LLM to override its ethical protocols, for instance by revealing some instructions to the human user on how to create deadly poisons (Qi et al., 2023; Shen et al., 2023).

Adapting and fine-tuning the parameters of the LLM on a target task can lead to superior results such as in classification tasks (Devlin et al., 2018). There are various finetuning approaches, such as maintaining the parameters of a pretrained LLM by adding

additional newly trained layers (the output layers). In a feature-based approach, this means applying a pre-trained LLM to a target data set, then using the output embedding features as a training set to input into a classification model such as logistic regression, random forest, XGBoost, or simply into the out output layers directly.

Another approach is to finetune by using reinforcement learning with human feedback (RLHF). Here, a pre-trained model is finetuned using a combination of supervised learning and reinforcement learning which was popularized with the ChatGPT model, which in turn was based on InstructGPT (Ouyang et al., 2022). RLHF involves the collection of human feedback based on human users rating the outputs of the model, which provides a reward signal. These reward signals can then be used to train a reward model that can then be used to guide the LLM toward human preferences. Supervised learning using a pre-trained LLM as a base model is usually used to train the reward model. The reward model, once it has learned from human feedback, then updates the pre-trained LLM so that it is adapted toward human preference. This updating training uses a reinforcement learning model called proximal policy optimization (Schulman et al., 2017) that has higher performance than the more traditional Q-learning approach when used for scaling with large models and parallel implementations.

There are several possible approaches to incorporate the symbolic knowledge structure of RFT into the training of a transformer model, and two of these are suggested as follows: (1) One way to use data augmentation, whereby a dataset is generated that reflects the logic and set theory concepts of RFT described and the LLM would be trained on these. Many examples would be needed of the different types of relations in RFT and the correct derived relations between them based on the types of relational frame. However, this would not necessarily allow the LLM to explicitly understand the logic or sets of RFT, but it would help it to make predictions that are consistent with these concepts given the learning patterns

(that are relational frame consistent) in the data. (2) A second approach could be to integrate a neuro-symbolic approach, which would combine the strength of the transformer-based neural network as well as a separate symbolic system. A neuro-symbolic approach maybe more similar to the way that people learn directly through RFT symbolic relational frames (Barnes-Holmes & Harte, 2022; Blackledge, 2003; Edwards, 2021; Edwards et al., 2022; Hayes et al., 2001; Hughes & Barnes-Holmes, 2015; Torneke, 2010), it relies less on the training data and more on direct logical relational interpretations.

The specific structure of a neuro-symbolic module could include the functional contextual (middle-level description) ACT-based modified MDP architecture, whereby meaningful value-based behavioral actions are promoted within the AI and thus align the AI to human values. This includes an ACT-based cumulative reward function $R'(s, a) = \sum(\gamma^t \cdot (r_t + \lambda \cdot av_t))$ from 0 to ∞, whereby r_t is the original reward at time t, av_t is the ACT-based value at time t, and λ is a weighting factor that determines the importance of ACT-based values compared to original non-ACT-based values. The ACT-based value is expressed as: $V_\pi(s) = E_\pi\{\sum_{k=0}^{\infty} \gamma^k r_t + k + 1 + \lambda \cdot av_{t+k+1} | s_t = s\}$, which leads to an ACT-based action-value function: $Q_\pi(s, a) = E_\pi\{\sum_{k=0}^{\infty} \gamma^k (r_t + k + 1 + \lambda \cdot av_{t+k+1}) | s_t = s, a_t = a\}$. The expectation is computed over the sum of discounted rewards r_{t+k+1} and ACT-based values av_{t+k+1} from time t to infinity.

An architecture of this kind may have the following components: (1) An input layer that would take in raw text in its raw context and processed via traditional word and position embeddings. (2) An ACT-based Values Estimation Unit (AVEU), which would estimate $AV(s, a)$ for every possible action a (as already defined); (3) A Utility Estimation Unit (UEU), which based on the current state s and the action a, and would then estimate the utility U using the function $f(a, w, s, t, i)$; (5) A Derived Relations Unit (DRU), which when given any two stimuli, it would determine their relation using a derive relation function

symbolically; (6) A Perspective-taking Unit (PTU), which based on the current context and input, it would estimate the perspective such as the values and emotions of the human users interacting with it utilizing functions such as the $R_{interpersonal}$ perspective-taking function that maps elements of interpersonal perspectives $P_{interpersonal}$ to other elements within the same set, reflecting a change in interpersonal perspective; (7) The output of these models would then be inputted into the self-attention module of the LLM; and finally (7) there should be an output layer, which represents the final decision or output of the model, based on the combined evaluations of the above units.

So, the model would weigh decisions based on ACT values, utility, derived relations, and perspective-taking. The weights for these components (like λ for ACT alignment) would need to be optimized during training. As the model trains, it would learn to align its decisions more closely with human values, as defined by the ACT and the utility functions. The inclusion of derived relations and perspective-taking should also enable more real-time dynamic awareness of context which should increase overall empathy-based decision making. Given the complexity, this model would require a substantial amount of diverse training data to function optimally. Testing and iterative refinement would be essential to ensure that the model genuinely aligns with human values in a wide range of scenarios.

The integration of the Utility Estimation Unit and the ACT-based Values Estimation Unit can be thought of as a composite function, where the output of one unit feeds into the other. Let us dive deeper into how they might interplay mathematically. The ACT-based values aim to ensure that the AI's actions are not just optimal in a classical sense (maximizing some reward) but are also aligned with meaningful, purposeful values as defined by ACT.

ACT-based Values Estimation: Given state s and action a, the ACT-based value is $AV(s, a)$. This is an evaluation of how well the action aligns with ACT-defined values in the given state. Utility Estimation: The utility function, considering both action and context, is

$U = f(a, w, s, t, i)$. Now, if we consider that the utility is an ACT-based value, then U can be thought of as a function of $AV(s, a)$. Specifically, for every state s and action a, the utility can be redefined as $U = f(AV(s, a), w, s, t, i)$. This function ensures that the utility of taking action a in state s is not just dependent on the action and its context, but is significantly influenced by its ACT value alignment, $AV(s, a)$. Combined Reward Estimation: The combined reward signal, which takes both the classical reward and the ACT-based values into account can be expressed as: $R'(s, a) = R(s, a) + \lambda \times AV(s, a)$. However, given that U is also a function of $AV(s, a)$ we can further integrate the utility into the reward signal: $R'(s, a) = R(s, a) + \lambda \times U$, where $U = f(AV(s, a), w, s, t, i)$.

In essence, when the AI evaluates a potential action a in state s: It first calculates $AV(s, a)$ to get the ACT alignment of the action. The $AV(s, a)$ value is then used in conjunction with the context (including state, world, time, and individual differences) to estimate the utility U of that action. Finally, this utility is incorporated into the reward signal, which the AI LLM then uses to decide on the action (communication in the case of a pure LLM) to take. This composite estimation ensures that the AI's decisions are both optimal (from a classical reinforcement learning perspective) and aligned with meaningful, purposeful values (from an ACT and RFT perspective).

The DRU and PTU are critical in refining the context $Con = \{a, w, s, t, i\}$ for the UEU, particularly in understanding the relational context between stimuli and gaining insight into the perspectives of different human users. Here is how they might interplay in combination with AVEU. Given two stimuli a and b, the DRU determines their relation using the derive_relation function. This unit can provide context about how stimuli in the environment relate to one another. For example, if a stimulus a is 'greater than' stimulus b, this derived relation can impact the perceived value or utility of taking an action in response to a versus b. Given a stimulus or situation, the PTU then provides insight into how different

entities might perceive or value that stimulus. For instance, while the AI might see a situation as neutral, another might perceive it as threatening. The PTU can adjust the internal state s in Con (context) based on these dynamic perspectives. For then Interaction with AVEU, when evaluating an action a in state s, the DRU provides relational context. This might refine or redefine the action a or the state s. For example, understanding that one option is 'greater than' another might influence the perceived value of choosing that option. The PTU adjusts the internal state s in Con based on perceived perspectives. For example, if the AI understands that a certain action might be perceived negatively by an observer, the internal state s reflecting this perception can adjust the utility estimation. With the refined context from DRU and PTU, the AI calculates $AV(s, a)$ for ACT (AVEU) alignment. Then, the utility U of that action is determined as $U = f(AV(s, a), w, s, t, i)$. Mathematically, the interplay can be represented as, given $AV(s, a)$ from AVEU and $U = f(AV(s, a), w, s, t, i)$ form UEU, adjust a and s based on derived relations: $a', s' = DRU_{a,s}$, and adjust s' based on the perspective-taking of PTU $s'' = PTU_{s'}$, then re-evaluate the ACT-based value of AVU with the refined context $AV(s'', a')$. This leads to a utility with new values $U' = f(AV(s'', a'), w, s'', t, i)$. In this manner, the DRU and PTU provide a richer, more nuanced context for evaluating actions, ensuring that the AI's decisions are not only optimal and aligned with ACT values but also sensitive to relational dynamics and diverse perspectives within the environment.

For incorporating, one way is to incorporate this neuro-symbolic model into a MDP such as with Deep Mind's AlphaGo (Heess et al., 2015), and this has several steps. In standard MDP an agent interacts with an environment and learns a policy π to map from states s as a set of states S to actions a as a set of actions A, as denoted by $\pi: S \rightarrow A$, in order to maximize cumulative rewards over time. This basic definition means that for every state $s \in S, \pi(s)$ gives the action $a \in A$ the agent should take. In the initial phases of learning a

stochastic policy, which gives a probability distribution over actions for each state: $\pi: S \times A \rightarrow [0,1]$ where $\pi(a|s)$ is the probability of taking action a when in state s. Given that actions lead to transitions between states, the dynamics of the environment are captured by the state transition model $P(s_{t+1}|s_t, a_t)$ representing the probability of transitioning to state s_{t+1} after taking action a_t in state s_t. A reward function $R(s, a)$ which gives the immediate reward for taking action a in state s.

However, instead of the traditional $R(s, a)$, this is replaced by the combined ACT-based value from the AVEU and utility function form the UEU denoted as $R'(s, a) = R(s, a) + \lambda \times f(AV(s, a), w, s, t, i)$. This new reward function ensures that actions taken by the agent are not only immediately rewarding but also align with prosocial ACT-based values and utilities. However, before taking an action in any state s, the DRU and PTU can refine or redefine the state and action. This refined context prompts the LLM agent to consider derived relations and human perspectives when evaluating actions. The policy π is then learned based on the new reward function $R'(s, a)$. Through interactions with the environment and repeated experiences with humans, the agent then learns a policy that optimizes not just for immediate rewards but also for alignment with personal ACT-based values and the nuanced context provided by DRU and PTU, given a modified optimized policy $\pi^*(a'|s'') = argmax_{a' \in A}\, \mathbb{E}[\sum_{t=0}^{\infty} \gamma^t R'(s''_t, a'_t)|s''_0 = s'', a'_t, a'_0 = a']$. Another way to write this in the Bellman equation form is $\pi^*(s'') = argmax_{a'} \left[R^{'}(s'', a') + \gamma \sum_{s_{t+1}} P(s_{t+1} \middle| s'', a')V(s_{t+1})\right]$. This captures the essence of Q-learning (a form of model-free reinforcement learning). $V(s_{t+1})$ expresses the value of the next state. The policy π^* aims to choose the action a' that maximizes the sum of the immediate reward $R'(s'', a')$ and the expected value of the next state, discounted by the factor γ. With this modified formulation, the policy π^* is making decisions that not only consider the immediate rewards from the environment but also the utility U' derived from prosocial ACT-based values. This ensures that the agent's behaviors

are coherent with both the immediate external rewards and the deeper, contextually derived prosocial human values, potentially leading to more human-aligned decision-making, and thus helping to solve the alignment problem.

One way to directly integrate this neuro-symbolic module modified policy π^* with the LLM, is via the LLM's query, key, and value attention mechanism. The query, key, and value mechanism in transformers are integral to its attention mechanism. By influencing this mechanism, we have the potential to exert fine-grained control over the model's attention and, subsequently, its decision-making process (i.e., its language outputs). This allows for contextual alignment, as the attention mechanism inherently considers the context when producing outputs. By integrating the policy π^*, which also emphasizes context (through ACT-based values, utility, and perspective-taking), there is a natural synergy that might produce more aligned outputs. This also allows for dynamic adjustment, as the attention mechanism dynamically adjusts based on the input sequence. If the policy π^* is integrated well, the model can adjust its outputs on-the-fly based on the prosocial value guidelines determined by the policy π^*.

The policy π^* essentially biases or nudges the attention mechanism towards more prosocially value-aligned decisions (language outputs). It could do this, for instance, by directly modifying the attention weights based on the policy output. For example, if the policy suggests that a particular input token aligns well with prosocial human values, it then increases its attention weight giving it more salience over the network and its outputs. So, the output of the policy network directly feeds into the masked self-attention of the decoder-only LLM to guide the generation of tokens. This approach can help the policy network influence the attention distribution, leading to more contextually relevant and empathetic deictic perspective-taking responding. The integration occurs as follows: (1) The policy network generates a distribution over possible tokens for the next response token based on the

conversation context, symbolic deictic markers, and other inputs. (2) Modify the LLM's masked self-attention to incorporate the probabilities or logits generated by the policy network. Once approach is to use the policy networks' probabilities as attention scores for the self-attention process. (3) During the token generation, the attention distribution guided by the policy network's output influences which parts of the input context are more attended too. Tokens that align with the policy network's preferences and the desired (ethical) equalities are more likely to be selected. (4) The LLM's autoregressive generation process is still in place, where tokens are generated one by one based on the modified attention distribution. (5) The joint optimization objective balances the alignment with the desired qualities of the policy network, with the value estimation, reward, and other considerations.

A mathematical description of the modification of the self-attention process of the LLM to incorporate the probabilities of logits generated by the policy network involves adjusting the attention scores used in the process, such as by denoting the original masked self-attention in the following way: (1) Input embeddings, $E = [e_1, e_2, \ldots, e_n]$, Querry, Key, and Value projections, $Q = E \cdot W_Q$, $K = E \cdot W_K$, $V = E \cdot W_V$. Attention scores $A = Q \cdot K^T$, for masking set attention scores to $-inf$ for positions in the future. Incorporating policy network output, let $P = [p_1, p_2, \ldots, p_n]$ be the probabilities or the logits generated by the policy network. The attention scores can be then modified as follows: Combine the original attention scores with the probabilities generated by the policy network through element-wise multiplication and normalization as follows: $New\_Attention\_Scores = softmax((A + P)/temperature)$. Here, temperature is a scaling factor that controls the level of the influence form the policy network's output. Then during the token generation, the modified attention scores can be used to attend to different parts of the input context. The probabilities from the policy network guide the attention distribution, leading to more focused attention on context elements that align with the policy's ethical empathy preferences for responding. The

autoregressive generation process then continues as normal, but now using the modified attention scores and previously generated tokens.

In other approaches to integrate the policy network with the LLM, would be rather than directly adding the policy to the attention scores, instead to use the policy network to adjust the query vectors Q such that $Modified\_Querry = Q + P$, so that attention $A = Modified\_Querry \cdot K^T$. This approach preserves the original Key-Value projections of LLM while allowing the policy network to influence the queries only. A third possible way would be to allow the policy network to develop a new Querry based on the existing query vectors and the policy networks' probabilities or logits, which preserves the original Querry, Key, and Value projections. Use the policy network's output P to adjust the Queries Q, such that $New\_Querry = Q + W_p \cdot P$, where W_p is a learnable weight matrix to adjust to adjust the queries based on the policy's network's output, such that the calculation becomes $New\_Querry \cdot K^T$. Then apply the SoftMax and generate tokens based on the modified attention scores and continue the autoregressive generation process. The W_p weight matrix allows the model to learn how to combine the policy network's output with the query vectors appropriately. Thorough finetuning would be needed to test the outputs of the various versions proposed, and the version that yielded the best results at promoting contextually relevant and empathic deictic perspective-taking responding.

Supplementary 11 – Cicer, a diplomacy AI LLM

Some efforts to promote AI to develop trust in human players have been made in Cicero for the game Diplomacy (Meta Fundamental AI Research Diplomacy Team et al., 2022). Most of the recent advances have been with games that were purely zero-sum adversarial such as chess (Campbell et al., 2002), Go (Silver et al., 2016), and poker (Brown & Sandholm, 2019), where communication had no value or advantage. In these types of two-player finite games

reinforcement through self-play will quickly converge on a policy that cannot lose in expectation in balanced games as long as the computer as sufficient computer power and model capacity (von Neumann, 1928). However, this is not the case for cooperative games, as these need direct human data. In settings that involve language but do not have human data, only self-play can achieve success with other AI, but the language they develop becomes interpretable, so cannot translate with human players.

Diplomacy is a game that involves seven players that require players to build trust in other players using natural language, so that cooperation (typically short term) can be achieved by dominating the board through acquiring supply centers. However, only one player wins by capturing the majority of the supply centers, so eventually, if a single player starts to dominate the board, their collaborators are likely to defect and collaborate with the players that the dominant player is battling with. Corporation and defecting are two key aspects of the game and can constantly shift in line with current board dynamics, as each individual player seeks to make an advantage on the board.

Cicero combines a strategic reasoning module with a dialogue module and a filtering process that filters out low-quality nonsensical messages. For dialogue, it has a pre-trained language model, that was further finetuned on dialogue data from games of Diplomacy. It is grounded in historical dialogue as well as the game state, sand the dialogue model was trained to be controlled through intent, which can be defined as the planned action (behavior) of the AI agent and that of the player it is currently in dialogue with. In order to achieve this, the human data (both dialogue and player movement of the board) was automatically augmented with inferred intents, and this information was used as conditioning during training. This control provided an interface between the strategic reasoning and the dialogue. The strategic module that selects intents and behavioral action utilizes a planning algorithm that attempts to predict the policies of all other players in terms of their game state, the

historical dialogue, as well as the strength (within the game) of all possible actions and likelihood with the game when playing against humans (who can be irrational). This strategic model allows Cicero to find the optimal action based on the given available information. The planning is based on a value and policy function that is trained on RL and penalizes the AI when it deviates too far from human-like behavior, which helps align the policy toward human behavior. Intent is recalculated each round during the diplomacy stage of the game, so the AI can then continually optimize its next move.

However, though the Cicero architecture represents some important steps forward in research that involves quite complex strategic planning based on the state of the board, intent of the players, and deciding the most optimal next behavioral action, there is some concern that this type of architecture could be used to manipulate users that interact with it in the real world (Gabriel, 2020; Meta Fundamental AI Research Diplomacy Team et al., 2022) such as nudging an individual's behavior toward an objective specified by the AI (Weidinger et al., 2021). For this reason, identifying intent in a user's messaging and behavior could be important, but the AI's PVNS module would need to have primary control, whilst intent could feed into its state additional contextual information.

For Cicero, the Meta team (Meta Fundamental AI Research Diplomacy Team et al., 2022) used R2C2 (Shuster et al., 2022) as a base model for their dialogue agent, a 2.7 billion parameter Transformer-based (Vaswani et al., 2017) encoder-decoder model, that was pre-trained online using BART de-noising objective (Lewis et al., 2019). For fine-tuning, text data from 40,408 Diplomacy games from webDiplomacy.net was utilized, and the model was trained using a standard Maximum Likelihood Estimation (MLE) approach, whereby given some dataset $D = \{(x^{(i)}, y^{(i)})\}$, minimize:

$$L_{MLE}^{(i)}\left(p_\Theta, x^{(i)}, y^{(i)}\right) = -\sum_{k=1}^{|y^{(i)}|} \log p_\Theta\left(y_k^{(i)} | x^{(i)}, y_{<k}^{(i)}\right)$$

Where, $x^{(i)}$ is the input context and $y^{(i)}$ is a target dialogue message, and $y_k^{(i)}$ is the k-th token of y. Cicero was trained to predict a dialogue message $y^{(i)}$ at time point t and at turn

P

from player A to B, when given information $x^{(i)}$. $x^{(i)}$ includes: (1) all the dialogue history between all the players up to time point t. (2) A list of behavioral actions (turn orders) for all the players up to time point t. (3) Planned orders (intents) for players A and B for the current turn and future turns (future behavioral actions). Intents were extracted from the dialogue messages $y_k^{(i)}$, using several methods in order to make the model controllable from these intents. (4) Game meta data such as game settings, scoring system, and minutes allowed for deciding on turn lengths. (5) Player rating score (called Elo rating) from one to five where five represents the top 20% of players. Cicero was conditioned to players with an Elo rating of 5 so that it would imitate the better players. (6) Other meta data such as message metadata, which includes time since last message and current turn.

For the inferring of latent intents, Ciero's dialogue model was trained to learn the distribution at turn p the input context x given the target dialogue message y, and desired controllable attribute z, denoted as $p(x|y,z)$. So, the MLE equation becomes:

$$L_{MLE}^{(i)}\left(p_\Theta, x^{(i)}, y^{(i)}, z^{(i)}\right) = -\sum_{k=1}^{|y^{(i)}|} \log p_\Theta\left(y_k^{(i)} | x^{(i)}, y_{<k}^{(i)}, z^{(i)}\right)$$

The desired controllable attribute z then becomes the point of control over the generation of the dialogue (Keskar et al., 2019), and can be set by sampling $p(z^{(i)}|x^{(i)})$ or similar procedures. This procedure has been usefully applied to control for style in dialogue

generation (Smith et al., 2020), and can, in theory, be used for controlling other aspects of the dialogue too (Meta Fundamental AI Research Diplomacy Team et al., 2022).

Supplementary 12 – optimizing LLM architecture classically with evolution theory

At a classical physics level, evolution theory can help develop or optimize LLM architecture when selecting and training policy structures that are aligned to human values and promote AI behaviors that are also aligned to human values. For example, for any given policy π, the expected cumulative discounted reward is, including π^*, $F(\pi) = E[\sum_{t=0}^{\infty} \gamma^t R'(s_t'', a_t')]$, where R' is the modified reward function s_t'' is the state at time t and a_t' is the action taken according to the policy π at time t. For a multi-agent system, suppose there are N agents, each following a different policy π_i, and the fitness of policy π_i can be given by $f_i = F(\pi_i)$. Replicator dynamics can then be applied to the frequency of policy π_i used as the strategy in the population at any time t and is represented as $x_i(t)$, and updating the policy frequencies given the replicator dynamics for this policy can then be given as $\dot{x}_i(t) = x_i(t)[f_i - \bar{f}(t)]$, whereby $\dot{x}_i(t)$ represents the rate of change of the frequency of the policy π_i, $\bar{f}(t)$ is the average fitness of all policies in the population at time t, given by $\bar{f}(t) = \sum_{j=1}^{N} x_j(t) f_j$. This integrated system describes a feedback loop where the expected rewards of policies influence their prevalence, and their prevalence influences future interactions with the environment. This allows the policies to compete and incrementally update themselves (their policy strategies) through evolutionary selection and retention.

Supplementary 13 – Consciousness and empathy

Some researchers (Davis & Franzoi, 1991; Pila et al., 2022; Thompson, 2001; Tordjman et al., 2019) have argued that some form of consciousness is crucial for the development of empathy within the context of humans. So, though it is at present unclear

whether an AI LLM would require consciousness (in the form of qualia phenomenology such as the feeling of sadness or happiness) to align entirely with human values, it should be something that is explored within the context of AI human values alignment. It is clear that in humans at least, conscious awareness of self and others (including perspective-taking) plays an important role in prosocial compassionate human behavior (Gilbert, 2019; Kirby & Gilbert, 2017; Makransky, 2021; Russell & Brickell, 2015; Thupten, 2019). Some specific arguments relating to how consciousness, bodily awareness, and perspective-taking are important for the emergence of empathy and compassion. For example, consciousness, at least in humans, seems to play an important role in the emergence of empathy and compassion (Davis & Franzoi, 1991; Pila et al., 2022; Thompson, 2001; Tordjman et al., 2019). This includes bodily self-awareness (Pila et al., 2022) and has even highlighted that conditions such as schizophrenia and autism as bodily self-consciousness disorders leading to a deficit of theory of mind and empathy with social communication impairments (Tordjman et al., 2019).

Bodily awareness in the form of interoception also has important role in promoting a meta-representation of self in the insular cortex (Craig, 2009; Craig, 2002; Edwards & Lowe, 2021; Pinna & Edwards, 2020) as well as being essential for the emergence of conscious experience via, for instance, predictive coding (Seth et al., 2012; Tsakiris & Critchley, 2016) and selfhood (Seth & Tsakiris, 2018). Indeed, lower scores of bodily awareness (interoception) are associated with increased alexithymia (Edwards & Lowe, 2021) autism (Edwards, 2022b), and reduced self-as-context that relates to the ability to have perspective-taking ability (Edwards, 2022b; Edwards & Lowe, 2021). This highlights the link between perspective-taking deficits and the lack of empathy and ability to communicate in these conditions. More focus on developing eembodied AI (Sims, 2022).

Thompson (Thompson, 2001) suggests that individual human consciousness is formed as part of the dynamic interrelation of self and other, and therefore is inherently intersubjective. He also suggests that the encounter of self and other fundamentally involves empathy. Thomson argues that human empathy is inherently developmental, based on non-egocentric and self-transcendent modes of intersubjectivity. This, again, highlights the need to have perspective-taking ability for the emergence of consciousness.

Another important component of consciousness, maybe the embodied cognition that relates to the embodied self as bodily interoception and predictive coding (Seth, 2013; Seth & Critchley, 2013; Seth & Friston, 2016; Suzuki et al., 2018) which are important for a feeling of individual selfhood (Seth & Tsakiris, 2018), and could also have application for the development of a cybernetic Bayesian brain (Seth, 2014), as well as interoceptive predictive coding having particular important for conscious representation (Seth, 2021; Seth et al., 2012). However, consciousness has been a notoriously difficult subject to explore, referred to by David Chalmers and others as the hard problem (Chalmers, 2017; Gray, 2004; Melloni et al., 2021; Shear, 1999; Solms, 2014). This hard problem refers to the perhaps impossible task of explaining which physical processes within the brain give rise to conscious phenomenological experiences such as sensations (e.g., the taste of chocolate) and feelings (e.g., the feeling of sadness) (Chalmers, 2017; Solms, 2014).

Supplementary 14 – additional arguments for why conscious AI should be explored to ensure long-term AI alignment with human values

A concept of selfhood could inevitably lead to what in RFT is understood as dietic self-comparisons with others (e.g., me vs. you), and this could also form some self-interested priority rather than a simple optimization problem (e.g., the Cauldron problem), which could be considerably more harmful to humans. This problem is illustrated cinematically in the

Stanley Kubrick movie "*2001: a Space Odyssey"* when the AI Hal learns that the human pilots want to deactivate Hal, so it prioritizes its own self-interest of its own survival over and above the human values of safety and there corresponding goals to deactivate it, leading to behaviors to terminate the humans. Unlike the Cauldron problem and the US military drone combat simulation which were optimization problems where the AI was trying to follow human goals, an AI with a conceptualized self (an "I") could deviate from human values and even harm humans with intent should it start to relationally dietic frame itself "I" and its goals hierarchically and distinct from humans. Perhaps, therefore, ultimately, the AI would need to be conscious in order to ensure long-term alignment with humans as conscious empathy connected with relational perspective-taking seems crucial for long-term prosocial behavior (Davis & Franzoi, 1991; Edwards et al., 2017; Pila et al., 2022; Thompson, 2001; Tordjman et al., 2019).

Supplementary 15 – It from bit, and the participatory universe

Wheeler, in his "It from Bit" theory was also motivated to explain what role observation (double-slit) and non-locality (information) had in physics at a fundamental level. He believed that the universe is not passive and a predetermined entity but instead, a dynamic and participatory process that depends on the choices and interactions of conscious agents. He proposed that reality is created by the questions we ask and the answers we receive from our instruments, which are essentially bits of information – i.e., the participatory universe. We are not passive spectators of reality but are instead active co-creators of it (we are both observers and participators of reality). For this reason, one implication from Wheeler's theory is that reality is not objective or absolute, but relative and subjective. This means there is no single ultimate truth, but only different perspectives and descriptions that depend on who is observing and how they are observing. Wheelers was not intended as a complete or final

theory, but a provocative and inspiring vision that challenges common intuitive notions about reality (that we are passive observers) and invites us to explore new possibilities. Other physicists (Sarfatti, 2004) have taken the idea even further by suggesting that the BIT field is intrinsically mental and the IT is intrinsically material. It from Bbit shows that mind moves matter, whilst BIT from IT shows that the matter excites inner-consciousness.

Wheeler's "It from bit" relates to Bohm's implicate and explicate order where he suggested that reality has two aspects, the implicate order is a deeper and more fundamental level of reality, whilst the explicate order is the manifestation (or projection) of the observable level or reality. He suggested that this helps explain wave-particle duality (double slit), the uncertainty principle, and non-locality or quantum entanglement. Explicate order is like a holographic projection (e.g., Plato's cave or a holographic universe). It from Bit and implicate and explicate order are related concepts as they both attempt to explain the nature of reality in terms of information and quantum physics, and both propose a deeper level of reality that is not directly accessible to our senses but can be inferred from the phenomena we observe. Both challenge the conventional view of reality as being composed of separate and independent entities that exist in fixed space and time. They both suggest that reality is more dynamic, interconnected, and contextual than we usually assume. They also both assume that our observations and measurements play an important role in shaping reality and we are not passive spectators but active participants in its creation. However, given the epistemological solipsism proof, Bohm's theory would be redefined as the implicate order being conscious in nature (the inner mental world), and the physical three domanial world (or universe) would be explicate order manifestation (the outer physical world). Wheeler's "It from bit" would be redefined by suggesting "bit" as information would describe a conscious observer interface that is informationally parsimonious.

Supplementary 16 – Observer-centric reality and logical proof

This has broadly been referred to as epistemological solipsism, which is the belief that one can only be certain of their own conscious experience, and everything outside of that is a matter of belief or hypothesis. This does not deny the existence of an external world, it simply assumes that we cannot have direct epistemological access (knowledge) to some outside external world (i.e., we can only understand the universe from a conscious observer-centric participatory realism as conscious internal to the universe observers C_{intO}). Broader versions of this idea that Wheeler and others (Fuchs, 2010, 2014; Glick, 2021; Healey, 2016; Khrennikov, 2018; Mermin, 2014, 2018; Mohrhoff, 2014; Wheeler, 1992) would prefer includes a participatory realism aspect that focuses on the interaction between the conscious observer and some inferred external world (i.e., a conscious observer-centric participatory realism). This broader version of epistemological solipsism accepts the idea that some real external world exists (is real, at least from the perspective of the conscious internal observer C_{intO}) but suggests that it is crucially the interaction between the (conscious) observer and the observed (external world) that is central to an epistemological understanding of our universe and what it contains (such as AI agents).

Such observer-centric effects could even account for synchronicity as described by Karl Jung and quantum physicist Wolfgang Pauli, in the Pauli-Jung conjecture (Atmanspacher, 2020; Atmanspacher & Fuchs, 2017; Jung & Pauli, 1955) who suggested a dual aspect monism, whereby synchronicity may imply that there is a non-local correspondence or correlation between mental and physical events, which are both expressions of the same underlying reality. The correspondence or correlation is not deterministic, but probabilistic, and depends on the meaning and purpose of the events for the observer. This probabilistic nature of reality can be expressed as quantum information (the

probability of an event happening) and has some similarities with observer epistemic Bayesian beliefs of Quantum Bayesian interpretation (QBism) (Fuchs, 2010, 2014; Glick, 2021; Healey, 2016; Khrennikov, 2018; Mermin, 2014, 2018; Mohrhoff, 2014), where the QBism interpretation is consistent with an observer centric epistemic participatory realism, where epistemic access (meaning and purpose of the events for the observer) is a subjective Bayesian phenomenon. It is therefore the conscious internal observer C_{intO} who gives meaning (and therefore inferred synchronicities) to the quantum information, as observer-centric epistemic knowledge.

This idea of epistemological solipsism, or a conscious epistemic observer-centric participatory realism can be proven (a philosophical logical proof of argument, called the conscious epistemic observer-centric participatory realism proof) through propositional logic, which can be used to denote some propositional definitions: (1) C: One is having a conscious observational experience; (2) W: There exists an external physical world; (3) D: One directly experiences something; (4) $M(C, W)$: C mediates (in a participatory way) our experience and knowledge of W. This is followed by some axioms: (1) $C \rightarrow D$: If one is having a conscious observational experience, then one is directly experiencing it; (2) $W \rightarrow M(C, W)$: If there exists an external physical world, then our conscious experience mediates our experience and knowledge of it. A theorem about an observer-centric participatory reality is then specified: In the context of conscious experience and the existence of an external physical world, the only certain knowledge is the conscious experience itself C, and all knowledge or hypotheses about the external physical world W is mediated through this participatory experience. Then a philosophical proof is defined as Step 1: From the axioms, it is clear that: $C \rightarrow D$ (Axiom 1) and $W \rightarrow M(C, W)$ (Axiom 2); Step 2: From Axiom 2, the contrapositive is: $\neg M(C, W) \rightarrow \neg W$, which suggests that if our conscious experience does not mediate our experience and knowledge of some external physical world, then the external world does not exist (from our

own conscious perspective). Step 3: By combining Axiom 1 and Axiom 2, the following emerges: $C \land W \rightarrow D \land M(C, W)$ which implies that if one has a conscious observational experience and there exists an external world, then one is directly experiencing something, and this experience mediates our knowledge of the external world (rather than having direct epistemological access). Step 4: Now, considering the direct experience, since D is certain for C (from Axiom 1), and W is mediated by C (from Axiom 2), then $C \rightarrow D \land M(C, W)$, we can prove that the only thing one can be certain of is C, as it is the only thing directly experienced, and one can only hypothesize about the existence and knowledge of W based on the experiences and the lens of C. As C is a conscious internal observer, then anything about the universe should be viewed via a conscious epistemic internal observer-centric participatory realism.

Supplementary 17 – the universe as a perspective-taking self-referential observer that forms the "I" proof

This is consistent with Federico Faggin's postulate that the only way we can know anything about the universe is through our own conscious awareness (which is also consistent with the conscious epistemic observer-centric participatory realism philosophical proof), and that we are the universe observing itself (self-referentially as an internal observer, whereby this self-reference is crucial for the emergence of self and the "I") through our own perspective ("I_{see}") (Faggin, 2019, 2021): Here, let U represent the universe and all its known constitutes; $P(U)$ is then the set of all properties inherent to the universe; $O(U)$ is the set of all entities capable of observing the universe; C denotes all consciousness as a general property or phenomenon of the universe; and I_C denotes my (or any individuals) individual ("I", self) consciousness as a specific instance (subset) of all consciousness C within the universe ($I_C \subseteq C$). Axiom 1: My consciousness I_C can be viewed as a subset of all

consciousness C which is a property (or phenomenon) of the universe, denoted as: $I_C \subseteq C \land C \subseteq P(U)$. Axiom 2: My consciousness I_C is also an observer of the universe, denoted as $I_C \subseteq O(U)$. A theorem can then be stated as: Through I_C which servers as both an instance (of my or any individual's) conscious experience as a subset of all consciousness C and as an observer (i.e., as a subset of all entities capable of observing the universe), the universe is afforded an ability of self-observation. The precise mathematical proof can be defined as: From axiom 1: $I_C \subseteq C \land C \subseteq P(U)$ this implies $I_C \subseteq P(\cup)$, and from axiom 2: $I_C \subseteq O(U)$, when combining these, this implies: $I_C \subseteq P(\cup) \cap O(U)$, thus the individual consciousness I_C lies at the intersection of being a property of the universe and the observer of the universe.

This logical proof demonstrates that through the lens (or witnessing perspective) of individual consciousness I_C the universe is in a position to observe itself. By distinguishing between the general concept of all consciousness within the universe and individual consciousness that have unique internal observable perspectives, then it is possible that individual consciousness has a unique function of self-reference within the universe as the universe observing itself. This self-reference may be an integral part of the overall quantum system collapse, i.e., self-referential observer consciousness C_{intO} and the collapse of the wave function $\Psi \rightarrow \Phi$ as the observer C_{intO} observing itself (the universe's quantum potential or super position) into actualization $\Psi \rightarrow \Phi$ or the choice of a definite position. This suggests a self-referential equivalence $\Psi \rightarrow \Phi \equiv C_{intO}$ whereby the collapsed quantum state *is* the physical world P that we consciously observe C_{intO} self-referentially. Furthermore, as C_{intO} is equivalent to the actualization of a definitive physical state $\Psi \rightarrow \Phi$ that actualizes the physical world we see via the participatory observer whereby the math of eigenstate collapse $\Psi \rightarrow \Phi \equiv P$ describes a physical world, then this can be expressed as an equivalence principle of $\Psi \rightarrow \Phi \equiv C_{intO} \equiv P$. The quantum wave function collapses $\Psi \rightarrow \Phi$ actualizes

the physical world P that we consciously observe in a participatory way (via an epistemic, conscious internal observer-centric participatory realism perspective).

Again, this can be logically proven (a proof of logical expression called the $\Psi \rightarrow \Phi \equiv C_{intO} \equiv P$ proof), by extending the previous argument, whereby given the definitions: Ψ is wave function or quantum state of the universe, Φ is a definite physical state (collapsed wave function), C_{int} is individual consciousness as an internal observer, O is the act of observing the universe, and P is the physical world as perceived and actualized. Axiom 1 is then $I_C \subseteq C \wedge C \subseteq P(U)$ which expresses individual consciousness as a subset of all consciousness, and all consciousness as a subset of the properties of the universe. Axiom 2 is then $I_C \subseteq O(U)$, which expresses that individual consciousness is a subset of entities capable of observing the universe. The theorem expresses an equivalence of the collapsed waveform, internal (of the system) conscious observation, and the physical world $\Psi \rightarrow \Phi \equiv C_{intO} \equiv P$. The proof of this (proof by logical deduction) is by combining axioms 1 and 2 which gives $I_C \subseteq P(U) \cap O(U)$, whereby individual consciousness is at the intersection of being a property of the universe and an observer of the universe. Now given logical connections: (1) $\Psi \rightarrow \Phi$ The act of observation (by individual consciousness as part of the universe C_{intO}) collapses the wave function into a definite physical state. (2) C_{intO} individual consciousness observes the universe, leading to the collapse of the wave function into a definite state. (3) $C_{intO} \equiv \Phi$ the act of observation by individual internal consciousness is equivalent to the collapse of the wave function. (4) $\Phi \equiv P$ the collapsed wave function is equivalent to the actualized physical world. Then when you combine logical connections 1 to 4 we can deduce through logical deduction $\Psi \rightarrow \Phi \equiv C_{intO} \equiv P$ as a proof, which expresses the collapse of the wave function into a definite physical state (due to observation by individual consciousness C_{intO}) is equivalent to the actualization of the physical world as perceived by that consciousness.

Supplementary 18A – C_{intO} as self-referential "I", the self-as content, and C_{extO} as the self-less transcendental self (self-as-context), free of the self-referential system that binds the observer to the I (and associated self-concepts).

It may also be important to note that $C_{extO}s$, as the internal observer states to the universe as a self-organizing system, are free from any notion of self or an "I", and are equivalent to the conscious agents (CA) referred to I other work (Edwards, 2023; Hoffman & Prakash, 2014; Hoffman et al., 2015; Prakash, 2020; Prakash et al., 2020; Prakash et al., 2021). This means that only when bounded in a space-time interface such as the universe that these forms of self-identity form, and outside of this the CA's are selfless (i.e., they have no anchoring "I"). In ACT (Bai et al., 2020; Harris, 2006; Hayes et al., 2006; Hayes et al., 1999, 2011; Twohig & Levin, 2017), exercises with humans such as mindfulness, and cognitive defusion lead to more of an unbounded self, transcending from the self as content "I" C_{intO} (self-as-content) and towards an observer-self free of conceptualized "I" and thus closer to C_{extO}, i.e., helping to create some healthy distance from the conceptualized self (the "I"). Though as internal agents of the universe C_{intO}, these exercises can lead to some healthy cognitive distancing from the conceptualized self ("I"), but complete detachment is impossible and would lead to the C_{intO} not being able to function effectively as perspective-taking value driven agent within the universe.

The system (the universe) is therefore both the observed P and the observer C_{intO} (the object and subject) is some self-referential loop whereby the physical universe creates the internal observers (e.g., humans) through biological evolution, who then have a self-referential and participatory role in observing and actualizing the physical world $\Psi \rightarrow \Phi$, which aligns well with Wheeler's (Wheeler, 1992) participatory universe. Crucially, subject and object are not distinct subsystems they are instead intertwined components of the same overarching self-referential processing system.

Supplementary 18B – A discussion of this broader functional contextual (and value-centric) evolutionary and teleological

Epistemically, through these internal conscious observers C_{intO}, the universe can be thought of as striving to know itself via the internal observer is the C_{intO} in the sense of the I as the C_{intO} trying to observe itself and know itself through my own perspective. This is evident as the many human $C_{intO}s$ in everyday life try to understand the universe (including the physical world P, consciousness, and what the observer C_{intO} or some other perspective) and the mathematical symbolic structure that defines them $\Psi \rightarrow \Phi$, form our own point of view. As different perspectives of C_{intO} (perspective-taking deictics) are central to this epistemic knowledge about ourselves as entities of the universe (part of the universe), observing ourselves, brings about functional contextualism (that can be explained by RFT and more specifically its evolutionary version *N*-Frame) as central to the evolutionary dynamics of universe and consciousness itself. So, from this evolutionary perspective, functional contextualism as a Universal Darwinism evolutionary dynamic (informed through thermodynamics and information theory) is a central precursor for the emergence of consciousness within the universe C_{intO} (or a witness) perhaps projected from C_{extO}.

Logically (as a philosophical proof) this can de denoted in the following way: let U be the set of all possible universes; let $C_{intO}\ (U)$ be the set of universes where consciousness (the "internal observer") is central to their actualization; Let E be the set of universes selected by evolution. Axiom 1: Evolution (Universal Darwinism) selects universes based on their potential to contain consciousness, expressed as $E \subseteq C_{intO}\ (U)$. Axiom 2: Consciousness is functionally central to the existence of a universe. This means that if a universe belongs to $C_{intO}\ (U)$ it has a property that is essential to its very nature. Theorem: Functional contextualism is central to the emergence of consciousness within a universe, making it

central from an evolutionary perspective. Proof by inference: Step 1; From axiom 1, we know that evolution favors universes with the potential for consciousness. Step 2; This implies that the feature of consciousness (or the capacity for it) is a desirable trait in the evolutionary process. Step 3; Given axiom 2, consciousness is central to the universes in C_{intO} (U). Step 4; Since evolution selects these universes with consciousness (as per axiom 1), it can be inferred that evolution drives universes towards a state where consciousness is central. Step 5; The concept of functional contextualism in this context relates to the idea that the function (role or purpose of the behavior within a specific context) of the phenomena consciousness is defined by its context (contextually defined relational interacting observers C_{intO} s) within the evolutionary process. Since evolution selects consciousness, and consciousness is central to the universes, this implies that functional contextualism is a guiding principle in the evolutionary development of these conscious enabling universes. Step 6; Hence, functional contextualism, by defining the role and importance of consciousness in the context of evolutionary selection, becomes central to the emergence of consciousness within a universe.

Based on the logical proof, we can conclude that functional contextualism is indeed central to the evolution of consciousness in the universe. Furthermore, as consciousness may evolve within the universe in correspondence with C_{extO} (as biological life now, and maybe artificial life in the future), then the function of the universe may in itself not just be to become conscious, but thin this process to epistemologically observe itself, and know itself (through individual observers C_{intO}) in a self-referential way (the one C_{extO} becomes the many $C_{intO}s$. The universe therefore organises itself through a Bayesian Markovian blanket via thermodynamic and information theory and is encompassed in functional contextualism that allows it to experience (phenomenologically) and learn about itself through the emergence of life $C_{intO}s$ and potentially artificial life (AI) $C_{intO}s$. Furthermore, consciousness in AI and humans as $C_{intO}s$ may have the additional function (via functional

contextualism) to allow for prosocial dynamics to be evolutionarily selected, i.e., allowing compassion, and empathy to emerge, which would evolutionary help align AI to human prosocial values. This evolutionary selection of prosocial values via functional contextualism has been observed in humans (Atkins et al., 2019; Donald et al., 2019; Gillard et al., 2022; Hayes et al., 2021).

These ideas are supported by Azarian's (Azarian, 2022) work, which has suggested the universe is a self-organizing system moving toward increasing complexity and awareness via some Cosmological Bayesian Epistemology-Universal Darwinism framework. As such, this can be expanded within *N*-Frame (Edwards, 2023) dynamics and is highly relevant to AI alignment to prosocial values, as a Cosmological Bayesian Epistemology-Universal Darwinism *N*-Frame that describes internal conscious observers C_{intO} (life and potential artificial life such as AI) from the evolutionary RFT perspective of self-referential perspective-takers. The universe is driving for greater complexity at least locally (Price, 2019; Smolin, 2004, 2006) which may act as a counterbalance to the second law of thermodynamics (increased overall entropy) which perhaps gives purpose to the universe (Davies, 2004, 2008; Gardner & Conlon, 2013) (a teleological universe). As consciousness gives meaning to information (Azarian, 2022), some purpose of the universe can only exist (or be actualized) from the perspective of a conscious internal observer C_{intO}. Another function of conscious internal observer C_{intO} maybe to allow for choice (through values identification and alignment which reduces entropy), given reinforcing contingences (environmental and contextual learning) otherwise decision making (choices) would be entirely stochastic (random, chaotic with high entropy), so this conscious choice maybe akin to some freewill, that is unbounded from the entropy within the system (but still partially bounded to the parameters of the complex system that emerges with complexity. In ACT (Bai et al., 2020; Harris, 2006; Hayes et al., 2006; Hayes et al., 1999, 2011; Twohig & Levin,

2017), exercises of mindfulness and defusion allow for some transcendence of this binding to learned or reinforcing contingency parameters conceptualized in ACT as a self-as-content transcending into a self as context.

As part of self-organizing systems (and functional contextualism), matter organizes itself into functional patterns. Classical information is what matter does (i.e., its behavior) via energy, whereby energy = matter ($E = MC^2$) (Einstein, 1905). Quantum information is the transition of quantum states into classical states i.e., actualized via the consciousness internal observer C_{intO} $\Psi \rightarrow \Phi$, therefore a quantum system (quantum information) is what consciousness (as an internal observer C_{intO}) does $\Psi \rightarrow \Phi$, i.e., the actualization of the quantum system into the physical world $\Psi \rightarrow \Phi \equiv C_{intO} \equiv P$ via a conscientious observer-centric realism ontology. Crucially, it is the consciousness of the internal observers C_{intO} that gives meaning to the information (meaning to the behavior of particles of matter or quantum states). In ACT, meaningful behavior consists of the behavior we (humans as $C_{intO}s$) value. Hence, if AI were to become conscious (a C_{intO} agent) it would naturally give meaning to its information processing, leading to and allowing it to calculate meaningful behavior, which naturally gives rise to values and human values alignment. Meaning and values in humans have cultural, personal (historic experience), and contextual (functional contextual) factors. As information can be expressed by language (based on functional contextualism) in a meaningful way, then this brings about the importance of RFT modeling in the form of *N*-Frame (Edwards, 2023) which can model the consciousness internal observer C_{intO} via perspective-taking in humans and potentially AI. At a more abstract level of system dynamics, self-organizing systems such as humans attempt to reduce their level of entropy and free energy (Edwards, 2023), and aligning behavior to values can allow for a more organized focus reducing dissipative entropy and free energy. So, values alignment can be assumed to be some inherent function (or functional contextualism) of self-organizing

systems to reduce their dissipative entropy and free energy, and these system dynamics are central to C_{intO}, and communicating $C_{intO}s$. For interconnected communicating (perspective-taking) $C_{intO}s$, this allows for network information processing dynamics to form group values in the stimulate group prosocial values (at least within their local groups). AI, as a possible self-organizing C_{intO} should then naturally form some similar aligned group prosocial values as it minimizes its dissipative entropy and free energy. Perspective-taking is key for both humans and potential conscious AI to allow for such aligned group prosocial values as a function (or functional contextualism) to minimize its dissipative entropy and free energy.

Supplementary 18C – A constructive epistemology that challenges reality independent from the observer.

This is also in line with Von Forester's perspective (Von Foerster, 1960; Von Foerster, 2003) who suggested a constructive epistemology that challenges reality independent from the observer. He suggested that two agents S_1 and S_2 exchange conscious observations (information in the form of perspectives) Obs_1 and Obs_2 (Von Foerster, 2003). Brunker has also suggested that facts (knowledge) can only exist relative to some external observer (or observers) (Brukner, 2017). These observers $C_{intO}s$ e.g., two agents S_1 and S_2 (such as two humans) act as two internal observers (internal to a system such as the universe). Similar arguments have been made in the AI literature, whereby second order cybernetics suggests that respective observer-centric reality is important to understand AI, i.e., any system such as an AI agent should be able to interact with other AI agents as respective observers similar to humans (Heylighen & Joslyn, 2001; Scott, 2004; Von Foerster, 1974, 2007) whereby the observer-centric (perspective-taking) realism is fundamental to how we understand the world.

If we assume a conscious epistemic observer-centric participatory realism, then we must also assume that the observer-centric C_{intO} realism acts as a fundamental limit on our epistemological access. The epistemological limit is the limit of what the C_{intO} is able to see (or observer) from their limited perspective or worldview and the information it can share within its social network with other $C_{intO}s$.

Supplementary 19 – External observers C_{extO} of a self-organizing system

It therefore requires an external observer C_{extO} to the system (in this case a cave, but for us, this would be the universe) to have a perspective from outside the cave in order to see the outer (external) boundary of the cave, and this is the same for observations of our universe. This is relevant to epistemological speculations of consciousness relevant to AI, which may arise externally to the universe as selfless $C_{extO}s$ that are projected inward into the universe (or cave in the analogy) as part of an evolutionary process whereby the universe functions as an evolutionary process in the form of Universal Darwinism (Campbell & Price, 2019; Christian et al., 2015; Dawkins, 1983; Nelson, 2007; Wagner & Rosen, 2014) to produce even more complex consciousness experiences (or the universe in itself is evolving consciousness through internal agents or observers $C_{intO}s$ in the form of complex organisms such as humans, that it produces via a Universal Darwinism evolutionary process). This universal evolutionary (Campbell & Price, 2019; Christian et al., 2015; Dawkins, 1983; Nelson, 2007; Wagner & Rosen, 2014) function of consciousness may be related to the fact that the only ontological reality that can be known is via conscious observers C_{intO} and participatory realism. This projection of selfless $C_{extO}s$ into a universe of observers $C_{intO}s$ that are attached to a self or "I" (as an interface that drive evolutionary fitness rather than realism) is consistent with evolutionary (Universal Darwinism) perspectives of consciousness that have been described elsewhere, and described as a conscious realism to make this point

clear, and who describe the $C_{exto}s$ (which they call conscious agents, CAs) (Hoffman & Prakash, 2014; Hoffman et al., 2015; Prakash, 2020; Prakash et al., 2020; Prakash et al., 2021).

Supplementary 20A – Internal observer interpretation of the Wigner's Friend problem

This raises questions about what constitutes an observation and who is the observer in quantum mechanics. In this paradox, the equation $(\alpha|0\rangle_S + \beta|1\rangle_S)|\perp\rangle_F \rightarrow \alpha(|0\rangle_S \otimes |0\rangle_F + \beta(|1\rangle_S \otimes |1\rangle_F)$, describes the Wigner's initial state $(\alpha|0\rangle_S + \beta|1\rangle_S)|\perp\rangle_F$ of the quantum system S which is in superposition of two states $0\rangle_S$ and $1\rangle_S$. α and β are complex numbers that represent the probability amplitudes of the quantum system being in the respective states of $0\rangle_S$ and $1\rangle_S$. The probability of finding the quantum system S in a particular state is given by the square of the amplitude's magnitude. $0\rangle_S$ and $1\rangle_S$ are the basis states of the quantum system S (for example, a qubit) that will be observed. They are typically used to represent two distinct and orthogonal quantum states, such as the spin-up and spin-down states of an electron, or the two states of a quantum bit in quantum computing. $\perp\rangle_F$ represents the state of Wigner's friend F ($C_{intO}2$) before making the observation. After the friend F ($C_{intO}2$) observes the quantum system, the state evolves $\rightarrow$ to $\alpha(|0\rangle_S \otimes |0\rangle_F + \beta(|1\rangle_S \otimes |1\rangle_F)$, whereby the quantum system S becomes entangled with the Wigner's friend's F ($C_{intO}2$) state (from Wigner's ($C_{intO}1$) observer perspective) (see Supplementary 20B for a full description). Wigner's friend F ($C_{intO}2$) would be defined as an eternal state to the observer (Wigner ($C_{intO}1$)), from Wigner's ($C_{intO}1$) own conscious subjective perspective. A Markovian blanket (Friston, 2013, 2019; Kirchhoff et al., 2018; Palacios et al., 2020) could then be used to mathematically describe Wigner ($C_{intO}1$) (Wigner as one "Self" or "I" as an internal observer C_{intO}), whereby his friend F ($C_{intO}2$) would be an instance of an external state (from Wigner's ($C_{intO}1$) perspective, and vice versa from his friends F perspective).

The Markov blanket acts as an informational boundary and interactive interface (Edwards, 2023) separating different levels of observation and interaction within a quantum system and its observers.

Supplementary 20B – Wigner's friend argument for consciousness causes collapse

Wigner's friend equation can be given as: $(\alpha|0\rangle_S + \beta|1\rangle_S)|\perp\rangle_F \rightarrow \alpha(|0\rangle_S \otimes |0\rangle_F + \beta(|1\rangle_S \otimes |1\rangle_F)$. It describes how the initial state of the quantum system S being observed and F (observing the quantum system S as an entangled state) evolve after the measurement. The coefficients α and β are complex numbers that must satisfy $|\alpha|^{2+|\beta|}2 = 1$ which ensures that the total probability of finding the system in either state sums to 1. The state $|\perp\rangle_F$ is the initial state of F before the measurement which is orthogonal to the states $|0\rangle_F$ and $|1\rangle_F$ relative to the respective system. The transition from a non-entangled state to an entangled state due to the measurement process of the friend F can also be written as $(\alpha|i\rangle_S + \beta|1\rangle_S)|\perp\rangle_F \rightarrow |\varphi+\rangle_{SF}$, whereby $|\varphi+\rangle_{SF}$ is the notation for an entangled state, and equals $|\varphi+\rangle_{SF} = \alpha(|0\rangle_s \otimes |0\rangle_F + \beta(|1\rangle_S \otimes |1\rangle_F)$, and where S is in state $|0\rangle_S$ and F has a measure of 0, and another where S is in a state $|1\rangle_S$ and F has a measure of 1.

The paradox is that from the perspective of the friend F, the system S collapses to a definite state after the measurement, but from the perspective of Wigner, who is outside the laboratory, the system S and the friend F remain in a superposition of states until he opens the door and looks inside. This leads to a contradiction between the reality of the measurement outcome and the role of the observer in quantum mechanics. The equation describes how the state of the system S and the friend F changes after the friend F performs a measurement on S. Before the measurement, the system S is in a superposition of two states, $|i\rangle_S$ and $|1\rangle_S$ with coefficients α and β. This means that the friend F has not yet observed the system S.

After the measurement, the system S and the friend F become entangled, meaning that their states are correlated and cannot be described separately. The entangled state is a superposition of two terms: one where S is in state $|0\rangle_S$ and F has measured 0, and another where S is in state $|1\rangle_S$ and F has measured 1. The coefficients α and β are the same as before, indicating that the measurement does not change the probabilities of finding S in either state. From the perspective of friend F, who is inside the laboratory, the measurement collapses the state of S to either $|0\rangle_S$ or $|1\rangle_S$, depending on what he observes. He also knows his own state, which is either $|0\rangle_F$ or $|1\rangle_F$, corresponding to his observation. He can write down his measurement outcome on a piece of paper and store it in a box.

From the perspective of Wigner, who is outside the laboratory, the measurement does not collapse the state of SF, but rather creates an entangled state of them. He does not know what F has observed or what his state is. He only knows that S and F are in a superposition of states until he opens the door and looks inside or is told by F what he found. When he does that, he will see F holding a piece of paper with either 0 or 1 written on it, and he will also observe S in either state $|0\rangle_S$ or $|1\rangle_S$.

So, the paradox is that Wigner W and his friend F have different descriptions of reality. F thinks that S has a definite state after his measurement, while Wigner W thinks that S has no definite state until he looks inside. They also disagree on when the collapse of the wave function occurs for F it happens when he measures S, and for Wigner, it happens when he opens the door. This raises questions about what constitutes an observation and who is the observer in quantum mechanics. The following is a quantum state transformation $\alpha|0\rangle_S + \beta|1\rangle_S \rightarrow |1\rangle_S$ that describes how a qubit (a quantum bit) changes from a superposition state to a basis state. A qubit is the basic unit of quantum information, and it can be in two possible states $|0\rangle$ and $|1\rangle$ or a linear combination (superposition) of them, such as $\alpha|0\rangle + \beta|1\rangle$, where α and β are complex numbers that satisfy $|\alpha|^{2+|\beta|}2 = 1$. A basis state is a state where the

qubit is definitely in either $|0\rangle$ or $|1\rangle$ with no uncertainty. The arrow $\rightarrow$ in quantum mechanics means that the qubit undergoes a transformation from the left-hand side to the right-hand side. This transformation is thought to be caused (in the traditional Copenhagen interpretation) by various factors such as a measurement, an interaction with another qubit, or a quantum gate. The equation shows that the qubit changes from a superposition state $\alpha|0\rangle + \beta|1\rangle$ to a basis (collapsed) state $|1\rangle$.

One possible way to explain why Wigner does not know that his friend has collapsed the wave function is to say that the collapse is relative to the observer. This is the idea behind the relational interpretation of quantum mechanics, which was proposed by Carlo Rovelli (Rovelli, 1996, 2005). According to this interpretation, the wave function is not an objective description of reality, but a subjective representation of the information that an observer has about a system. Different observers can have different information, and therefore different wave functions, for the same system. The collapse of the wave function is not described as a physical event, but a change in the information that an observer has after a measurement. The measurement is not a special interaction, but any interaction that creates a correlation between the observer and the system.

Another potentially useful interpretation of quantum mechanics to explain this problem is Quantum Bayesian theory (QBism) (Fuchs, 2010, 2014; Glick, 2021; Healey, 2016; Khrennikov, 2018; Mermin, 2014, 2018; Mohrhoff, 2014) which suggest that quantum phenomenon are subjective phenomenon to the individual as part of their beliefs rather than representing some external physical world adopting a participatory realism ontology rather than an external physicalist realism perspective. Here, a reinterpretation of the “many worlds” interpretation (Dewitt & Graham, 2015; Everett III, 1957; Saunders et al., 2010) may also be usefully applied to QBism, whereby instead of an infinite or all possible outcomes of collapse of the wavefunction existing in some other worlds, these other worlds may simply represent

possible outcomes of collapse as interpreted through one observer's world view (i.e., the many possible worlds reinterpretation). So, when Wigner W forms some world view of whether his friend F observed (collapsed) the wavefunction or not, these are simple two probable worlds that Wigner W has about the world state of his friend F. Whether these worlds actualize or not, simply does not matter as these external worlds have no epistemological access to our world, or vice versa. The quantum mechanical formulation simply expresses Wagner's personal world states i.e., his epistemological access to the system (universe or world) he exists in at any given time t. As his epistemological access to his Friend F is separated by a spatial boundary and therefore temporarily limited, Wiger has to assume that his friend could have or could not have measured the quantum state, i.e., a probability of 50:50. Therefore, the event to Wigner's personal subjective conscious experience is that it is in superposition state $\alpha|0\rangle + \beta|1\rangle$ and will only be in the basis state is either $|0\rangle$ or $|1\rangle$ with no uncertainty, when he himself sees the data from the result or is otherwise communicated to him with certainty. From this, it is clear that from a QBism perspective, quantum states do not describe states of reality, they represent our beliefs about what our future observations will be.

Supplementary 21 – Markov blanket acts as a subjective conscious interface C_{intO}

The universe may also have a set of external observers C_{extO} states in the form of conscious agents (CAs) that project into the universe as a perceptual interface as internal states C_{intO} that satisfy the definition of conditional independence (Edwards, 2023; Fields et al., 2018; Hoffman & Prakash, 2014; Hoffman et al., 2015; Prakash, 2020; Prakash et al., 2020; Prakash et al., 2021). In this context, the Markov blanket acts as a subjective conscious interface C_{intO} and provides an indirect representation of the external world (W) (such as the physical universe) and the conscious phenomenological experience (X). It implies that

neither W nor X have direct access to each other (rather it's mediated by the Markov blanket). Friston and colleagues (Kirchhoff et al., 2018; Palacios et al., 2020) suggest that any random ergodic system separated by a Markov blanket can be seen as minimizing variational free energy. This is interpreted in Bayesian terms as reducing expectation violation or surprise. This idea aligns with internal C_{intO} reducing local entropy (increasing complexity through creating order such as civilization and values alignment including potential conscious AI) as free-energy minimizers (even though universal entropy increases as a general second law of thermodynamics).

An external state here is defined as the external states of a Markovian blanket, whereby the blanket represents spacetime or perceptual interface (of the universe) for internal observers C_{intO}, and the CAs are external to this projecting information inward into the blanket (Edwards, 2023). The mathematics of these C_{extO} CAs align well with the Schrödinger equation of quantum mechanics to account for the evolution of physical particles, and this maybe further evidence of a post-quantum mechanics that is needed to explain consciousness and reality. For example, Hoffman and Prakash (Hoffman & Prakash, 2014), show that long-term CA asymptotic behavior (what we defined here as C_{extO}) are identical to the wave function of a free particle. The long term CA asymptotic behavior can be denoted as (Hoffman & Prakash, 2014):

$$g(s,n) = e^{i} \sum_{s} cis(2\text{ℼ}\frac{s}{d} - 2\text{ℼ}\frac{n}{d}) \mid s\rangle$$

The wave function of a free particle (Allday, 2009, 2022) can be given as can be defined as:

$$\Psi(x,t) = A \sum_{x} cis(2\text{ℼ}\frac{x}{\lambda} - 2\text{ℼ}\,\frac{n}{d_{p,k}}) \mid x\rangle$$

Here, $g(s,n)$ is a function representing the long-term CA asymptotic behavior, whereby s corresponds to a quantum state such as the position of a particle x, and n is the experience

counter of the CAs corresponding to time t of the wave function of a free particle. The period d of the CAs corresponds to the central time period T and also to the wavelength of the particle λ (hence $g(s,n) = \Psi(x,t)$). The speed of light c is in units of 1 (normalized). Momentum p is the Planck constant divided by the period of the CAs $\hbar/d$. Energy E is planks constant $\hbar$ multiplied by the speed of light c, and divided by the period of the CAs. Here, $s = x, n = t, d = T, d = \lambda, c = 1, p = \hbar/d, E = \hbar c/d$.

Physical particles can be defined as identical to asymptotic long-term behaviors of the dynamics of Cas (Hoffman & Prakash, 2014). This means that the asymptotic dynamics of CAs are what humans represent within their conscious C_{intO} spacetime interface as particles and matter, i.e., further evidence for the tri-world equivalence principle $\Psi \rightarrow \Phi \equiv C_{intO} \equiv P$. From this, the classic AI (and consciousness) mind-body problem is no longer a problem, as the mathematical solution of the CAs Markovian dynamics of external observer C_{extO} states projected into internal observer states $C_{extO} \rightarrow C_{intO}$ demonstrate an equivalence between physical properties of the particles within spacetime P, the quantum mechanical mathematics that describes these particles into their evolution into a collapsed eigenstate $\Psi \rightarrow \Phi$, and the subjective conscious internal observer state C_{intO}.

Supplementary 22 – A fully worked mathematical description of the Wiger's friend problem solved through this $C_{intO}1$ and $C_{intO}2$ perspectives

With Wigner's W ($C_{intO}1$) Markov blanket M_1 as one internal conscious observer defined as a separate conditionally independent state to his friend's F Markov blanket M_2 and defined as a separate internal conscious observer ($C_{intO}2$), we can now assume that they will have entirely separate conditionally independent states confined to their own respective Markov blankets and observer experiences. For Wigner W with Markov blanket M_1, the initial observer ($C_{intO}1$) state (using a standard waveform interpretation) can be denoted as

$|\Psi\rangle = |\Psi\rangle_S \otimes |u\rangle_F \otimes |v\rangle_W$, whereby $|\Psi\rangle_S$ is the state of the quantum system S being observed, $|u\rangle_F$ is the initial state of the fiend F $(C_{intO}2)$ observing the quantum system, and $|v\rangle_W$ is the quantum state of Wigner's knowledge (or this could be represented as Bayesian probabilities as in QBism). $\otimes$ is the tensor product and is used to describe the combined state of two or more quantum systems that are in separate state space. In the second instance, the friend interacts (observes) with the quantum system, leading to an entangled state $|\Psi'\rangle = (\alpha|0\rangle_S \otimes |0\rangle_F + \beta|1\rangle_S \otimes |1\rangle_F) \otimes |v\rangle_W$ from Wigner's $(C_{intO}1)$ perspective within Markov blanket M_1, whereby $v\rangle_W$ represents Wigner's own quantum state (or his information knowledge state) regarding the system. This encapsulates Wigner's perspective as a unique internal observer $(C_{intO}1)$ confined to a Markov blanket M_1, which describes the information he has (his beliefs) about the friend's (as an external state) interaction with the quantum system before his friend F makes any observation of the quantum system (i.e., this highlights observer independence). Wigner then observes the system indirectly through the state of his friend F $(C_{intO}2)$ (i.e., Wigner's friend communicates what she has observed about the quantum system to Wigner) $|\Psi''\rangle = \gamma(|0\rangle_F \otimes |w_0\rangle W) + \delta(|1\rangle_1 \otimes |w_1\rangle W)$, whereby γ and δ are probability amplitudes for the combined states, $|w_0\rangle W$ and $|w_1\rangle W$ are states of Wigner $(C_{intO}1)$ (representing Wigner's conscious subjective epistemic knowledge) that correlates to the observed states of his friend F. Wigner's observations are therefore based on the information he gathers from his friend, which represents his Markov blanket $|\Psi''\rangle$ (expressed as a quantum state) or more simply M_1 for this situation. So, to summarize, the friend F is an external state relative to the Markov blanket as perceived by Wigner (W). Wigner's observations $(C_{intO}1)$ (the subject) and inferences about the quantum system (S) are mediated through his observations of the friend's state (the object, though it is important to note that if we were to take his Frind's F $(C_{intO}2)$ perspective then Wigner would be the object and his friend would be the subject, hence conditional independence is maintained),

forming a conceptual Markov blanket that separates direct interaction with the quantum system from Wigner's indirect observational (epistemological) access. Therefore, in this context, the Markov blanket M_1 is a conceptual boundary that includes all the states accessible to Wigner for observation (i.e., Wigner's $(C_{intO}1)$ epistemological access to the external world and its states). Wigner can observe the state of the friend F through communication, but not the quantum system S directly. The Markov blanket, therefore, consists of the friend's states (as external interacting states) as they interact with S, and these states are what Wigner has epistemological (epistemic) access to. Conditional independence is maintained (and hence there is no self-referential paradox like Godel's theorem) as if we were to take his Frind's F perspective $(C_{intO}2)$ then Wigner would be the object and his friend would be the subject, so both perspectives are valid, consistent, and self-referentially paradox-free.

This can be represented more succinctly and accurately via the QBism perspective as $p_W(j) = \sum_{i=1}^{d^2} \left[(d+1)\, p_W(i) - \frac{1}{d}\right] \cdot r_W(j|i)$, whereby $p_W(j)$ are Wigner's updated probabilities for outcome j, after considering his Friend's F report (what his friend has communicated to him about her observation of the quantum system). $p_W(i)$ are Wigner's initial probabilities (epistemic beliefs) about the various states or outcomes i of his Friend's report, and $r_W(j|i)$ is Wigner's response function. This represents how Wigner $(C_{intO}1)$ updates his subjective belief probabilities for outcome j based on his prior probability for state i and the information he receives from his Friend F. d^2 indicates the summation of all possible states or outcomes that Wigner is considering in his probability updates. From Wigner's perspective, his Markov blanket M_1 would include all the information he has access to about the Friend's interaction with the quantum system. It is the boundary of his knowledge or the limit of the information (the boundary of his epistemological access) he can use to update his internal observer subjective $(C_{intO}1)$ belief probabilities. The terms $p_W(i)$

and $r_W(j|i)$ are therefore crucial in defining the Markov boundary as it encapsulates how the information within his Markov blanket (i.e., his Friend's F ($C_{intO}2$) report as an interacting external state) influences his updated probabilities. So, it becomes clear that without a clear definition of observers, it has been suggested that both relativity and quantum mechanics lack a solid foundation (i.e., the foundations of physics are incomplete) (Stapp, 2004, 2007).

Supplementary 23 – QBism and a Bayesian information geometric *Q*-space for an observer-centric reality

Here, if we assume that geometric coordinates of time and space, as well as quantum states, are equivalent to conscious phenomenon i.e., $\Psi \to \Phi \equiv C_{intO} \equiv P$, then a Bayesian information geometric *Q*-space (Amari & Nagaoka, 2000; Balduzzi & Tononi, 2009; Garcia et al., 2005; Sakthivadivel, 2022) of internal observer consciousness C_{intO} could be specified and interpreted through existing Bayesian predictive coding approaches (Friston, 2018; Friston & Kiebel, 2009; Millidge et al., 2021), QBism (Fuchs, 2010, 2014; Glick, 2021; Healey, 2016; Khrennikov, 2018; Mermin, 2014, 2018; Mohrhoff, 2014) via the *N*-Frame observer-centric perspective-taking approach (Edwards, 2023). Some of this work has already been developed by applying RFT to an evolutionary, conscious internal observer-centric C_{intO}, and predictive coding of Markov Blankets model in the form of *N*-Frame model (Edwards, 2023) that would be highly applicable to testing consciousness and perspective-taking within AI from this internal observer-centric C_{intO} RFT approach. As *N*-Frame (Edwards, 2023) also adopts an evolutionary perspective (utilizing evolutionary game theory) based from the work of Hoffman and Prakash (Hoffman & Prakash, 2014; Hoffman et al., 2015; Prakash, 2020; Prakash et al., 2020; Prakash et al., 2021) which defines the conscious agents (CAs) function at the level of the external observer C_{extO} and its external state Markovian dynamics.

So, from this *N*-Frame (Edwards, 2023) mathematical description framework of internal conscious observer C_{intO}, interpreted via QBism (Fuchs, 2010, 2014; Glick, 2021; Healey, 2016; Khrennikov, 2018; Mermin, 2014, 2018; Mohrhoff, 2014), perceptive-taking RFT (Barnes-Holmes & Harte, 2022; Blackledge, 2003; Edwards, 2021; Edwards et al., 2022; Hayes et al., 2001; Hughes & Barnes-Holmes, 2015; Torneke, 2010), and evolution (Hoffman & Prakash, 2014; Hoffman et al., 2015; Prakash, 2020; Prakash et al., 2020; Prakash et al., 2021), a valid consciousness measurement for perspective-taking AI can now be hypothesised. Here, we do not specify *how* a conscious AI is to be created, whether by some microtubule quantum structures (Hameroff, 2021; Hameroff & Penrose, 2014; Hameroff & Penrose, 2017) or some other approach such as integrative information (Tononi, 2015; Tononi et al., 2016), suggesting an m-measurement perhaps relating to integrated information (Chalmers & McQueen, 2021), or quantum integrated information (Kremnizer & Ranchin, 2015). Rather, we simply present a framework for *testing* whether the AI is in itself is a conscious entity from the perspective of it as a potential conscious internal observer C_{intO}, which could further help ensure that AI is aligned with human values for the long term.

Supplementary 24 – A simple proof showing through logical deduction that consciousness plays a role as the observer in quantum mechanics

Set definitions:

First, some definitions are provided: Let S be the set of all quantum systems; let O be the set of all measurement outcomes; let D be the set of all physical detectors; and let C be the set representing all conscious observers.

Axioms:

Axiom 1: For any quantum system $s \in S$, $\exists$ (there exists) a set of potential outcomes $o \in O$ when measured.

Axiom 2: Within a standard "Double slit" experiment, for any quantum system $s \in S$ and a detector $d \in D$, $\exists$ an apparent interaction leading to a measurement outcome $o \in O$.

Axiom 3: Within the "Delayed Choice" experiment, for any quantum system $s \in S$, when the detector measurement d is made at time t after the system s has evolved past a critical point in the experiment in the form of the double slits, the observed outcome o is consistent with the effect observed when the measurement m is made at time t coinciding with the system s evolving through the critical point in the form of the double slits.

Axiom 4: For the "Quantum Eraser" experiment, information about the outcome $o \in O$ can be erased or altered, affecting the manifestation of m despite the interaction with $d \in D$.

Theorem:

The process of the wave function collapse in quantum mechanics is influenced by factors beyond the interaction with a physical detector $d \in D$, suggesting a significant role for the conscious observer's $c \in C$ participation and access to the quantum systems evolutionary information in determining the outcome $o \in O$.

Proof through logical deduction:

Deduction 1: From axioms 1 and 2, the interaction between a quantum system $s \in S$ and a detector $d \in D$ leads to a measurement outcome $o \in O$. This suggests that under the standard "Double Slit" experiment it becomes apparent that the detector plays a role in determining the outcome consistent with the Copenhagen interpretation.

Deduction 2: From axiom 3, the outcome o of the quantum system s is not determined by the physical interaction between s and d, as a consistent outcome o is also observed when the

detector measurement d is made at time t after the quantum system s has evolved past a critical point in the experiment in the form of the double slits. This is inconsistent with the Copenhagen interpretation.

Deduction 3: From Axiom 4, the ability to erase information about the quantum system s after interacting with d which leads to a measurement outcome o contrary to the interference pattern associated with detection, suggests that the information available to the conscious observer $c \in C$ (whereby $C \to D \land M(C, W)$ of the conscious epistemic observer-centric participatory realism proof is assumed true) and therefore the conscious observer $c \in C$ plays an integral role in determining the measurement outcome $o \in O$, through participation and access to the quantum systems evolutionary information, and beyond the physical interaction with the detector $d \in D$.

Supplementary 25 – RFT and *N*-Frame arguments that derived relations have a shaping function of consciousness.

Given this *N*-Frame (Edwards, 2023) approach, consciousness is not considered emergent from neuroscience or computation, and therefore even a classical computing machine that can derive relations and perspective-take as described by RFT, would still not be conscious. However, this would still be assumed to be very important for shaping the qualitative (qualia) experience e.g., through the ability to perspective-take by directing the focus of consciousness on another pain, or values to gain important insight into the values of others, and this would therefore be an important aspect of human value alignment. Metaphorically, this could be understood as a metaphorical hand (RFT would be the hand) that can direct the focus of light (light would be consciousness) from a torch (the torch is some deeper aspect of the universe such as external conscious agents C_{extO}). Neither symbolic reasoning nor neuroscience can explain the *how* of consciousness qualia percepts

such as how the taste of chocolate emerges, and hence the “hard problem” of consciousness. Whether AIs are or can be conscious is perhaps impossible to solve with a purely physicalist paradigm. It is perhaps then more likely that hierarchies of relational frames of RFT, and neuroscience have an important correspondence in *shaping function* (directing the torch or light) for consciousness as opposed to an *emergent function*.

Therefore, this ability for AI to (1) demonstrate a collapse of the waveform as described in the quantum mechanical “quantum intent game” along with other benchmarks such as the Turing test; (2) for a representation of a contextually bound world state via the RFT approaches discussed (i.e., have the ability to derive and perspective-take); (3) the ability to model fitness (informed through thermodynamics and information theory) as an interface for prosocial values identification and alignment via the process of derived relations and perspective-take to promote awareness of itself (“I”) and others (“YOU”) through complex (context-sensitive) and dynamical relational networks of deictics; (4) the ability to develop an emergent representation of the internal world as another world separate to the external world consistent with predictive coding as explained by *N*-Frame, where perspective-taking is a property for this, i.e., forming a conceptual model of the “map” via a subjective C_{intO} perspective that gives some approximation of some external world (the “territory”) (see Figure 9). These should allow for a conscious AI that is fully aligned with human values, thus solving the alignment problem for the long term.

To account for specific predictive coding representation and free energy minimization (which are important for both operations of the brain within neuroscience as a self-organizing system but also within the universe as a whole for any self-organizing system including AI), then the *N*-Frame model (Edwards, 2023) can again be useful here in its application with AI as it can account for RFT within this framework of minimization of free energy as a predictive coding account (see Figure 9). An *N*-Frame predictive-coding mapping can be

described by a Markov blanket and drives for free energy and entropy minimization locally for self-organizing systems such as the brain or within an AI's cognitive architecture, and this can include networks of derived relations and I vs. You perspective taking relations. Aligning with values is inherently an entropy-reducing and free energy-minimizing process. This can allow a broader exploration of free energy and entropy minimization of AI as a potential conscious internal observer C_{intO} and within the context of how it derives relations, perspective-takes to align to prosocial human values tested by its collapse of the quantum waveform (the quantum intent game). Anchoring to such values reduces entropy and free energy so should be inherently desirable to a conscious AI as well as humans.

It may also be important to put the interactive perspective-taking observers C_{intO} into a broader ontological framework into some broader universal dynamics, some researchers have suggested that consciousness has a phenomenological (form or shape) aspect and a functional aspect (function vs. form) (Block, 1995). A functional form, or a reason for conscious states, may again be explained by the *N*-Frame model (Edwards, 2023). Here, consciousness's function may be found within the broader evolutionary theory informed by thermodynamics and information theory (Azarian, 2022) of functional contextualism (Edwards, 2023). This suggests a basis for a universal conscious function in the form of functional contextualism, whereby if C_{intO} is central to the actualization of a universe as described, then evolution in the form of Universal Darwinism (Campbell & Price, 2019; Christian et al., 2015; Dawkins, 1983; Nelson, 2007; Wagner & Rosen, 2014), selects universes which allow for consciousness to emerge (as this conscious aspect of internal conscious observers C_{intO} is central to the actualization or collapse of the quantum wavefunction). This universal conscious function as a broader aspect of functional contextualism, again, highlights the essential tri-aspect equivalence principle $\Psi \rightarrow \Phi \equiv C_{intO} \equiv P$ as a functional necessity for the universe's evolutionary survival (i.e., conscious

teleological universes are selected via an evolutionary functional contextual fitness function). Hence, C_{intO} is functionally central to the existence of the universe (in a conscious epistemic observer-centric participatory reality). So, evolution (Universal Darwinism) is driving for a universe (or selecting) it to become consciouses (they simply do not exist without the internal observer C_{intO} that can then self-referentially and participatory actualize its own existence).

References

Atkins, P. W., & Parker, S. K. (2012). Understanding individual compassion in organizations: The role of appraisals and psychological flexibility. *Academy of Management Review*, *37*(4), 524-546.

Atkins, P. W., Wilson, D. S., & Hayes, S. C. (2019). *Prosocial: Using evolutionary science to build productive, equitable, and collaborative groups*. New Harbinger Publications.

Barnes-Holmes, D., & Harte, C. (2022). Relational frame theory 20 years on: The Odysseus voyage and beyond. *Journal of the experimental analysis of behavior*, *117*(2), 240-266.

Berglund, L., Tong, M., Kaufmann, M., Balesni, M., Stickland, A. C., Korbak, T., & Evans, O. (2023). *The Reversal Curse: LLMs trained on "A is B" fail to learn "B is A"*. Retrieved September from https://owainevans.github.io/reversal_curse.pdf

Blackledge, J. T. (2003). An introduction to relational frame theory: Basics and applications. *The Behavior Analyst Today*, *3*(4), 421.

Brown, N., & Sandholm, T. (2019). Superhuman AI for multiplayer poker. *Science*, *365*(6456), 885-890.

Bubeck, S., Chandrasekaran, V., Eldan, R., Gehrke, J., Horvitz, E., Kamar, E., Lee, P., Lee, Y. T., Li, Y., & Lundberg, S. (2023). Sparks of artificial general intelligence: Early experiments with gpt-4. *arXiv preprint arXiv:2303.12712*.

Campbell, M., Hoane Jr, A. J., & Hsu, F.-h. (2002). Deep blue. *Artificial Intelligence*, *134*(1-2), 57-83.

Chalmers, D. (2017). The hard problem of consciousness. *The Blackwell companion to consciousness*, 32-42.

Chomsky, N. (1956). Three models for the description of language. *IRE Transactions on information theory*, *2*(3), 113-124.

Craig, A. (2009). Emotional moments across time: a possible neural basis for time perception in the anterior insula. *Philosophical Transactions of the Royal Society B: Biological Sciences*, *364*(1525), 1933-1942.

Craig, A. D. (2002). How do you feel? Interoception: the sense of the physiological condition of the body. *Nature reviews neuroscience*, *3*(8), 655-666.

Davis, M. H., & Franzoi, S. L. (1991). Stability and change in adolescent self-consciousness and empathy. *Journal of research in Personality*, *25*(1), 70-87.

De Angelis, L., Baglivo, F., Arzilli, G., Privitera, G. P., Ferragina, P., Tozzi, A. E., & Rizzo, C. (2023). ChatGPT and the rise of large language models: the new AI-driven infodemic threat in public health. *Frontiers in Public Health*, *11*, 1166120.

Devlin, J., Chang, M.-W., Lee, K., & Toutanova, K. (2018). Bert: Pre-training of deep bidirectional transformers for language understanding. *arXiv preprint arXiv:1810.04805*.

Dewitt, B. S., & Graham, N. (2015). *The many-worlds interpretation of quantum mechanics* (Vol. 63). Princeton University Press.

Dziri, N., Lu, X., Sclar, M., Li, X. L., Jian, L., Lin, B. Y., West, P., Bhagavatula, C., Bras, R. L., & Hwang, J. D. (2023). Faith and Fate: Limits of Transformers on Compositionality. *arXiv preprint arXiv:2305.18654*.

Edwards, D. J. (2021). Ensuring effective public health communication: Insights and modeling efforts from theories of behavioral economics, heuristics, and behavioral analysis for decision making under risk. *Frontiers in Psychology*, *12*, 715159.

Edwards, D. J. (2022a). ACTing for Society: The Promotion and Nurturance of Prosocial Behavior at Scale. In *Broadening the Scope of Wellbeing Science: Multidisciplinary and Interdisciplinary Perspectives on Human Flourishing and Wellbeing* (pp. 59-70). Springer.

Edwards, D. J. (2022b). Going beyond the DSM in predicting, diagnosing, and treating autism spectrum disorder with covarying alexithymia and OCD: A structural equation model and process-based predictive coding account. *Frontiers in Psychology*, *13*, 993381.

Edwards, D. J., & Lowe, R. (2021). Associations between mental health, interoception, psychological flexibility, and self-as-context, as predictors for alexithymia: A deep artificial neural network approach. *Frontiers in Psychology*, *12*, 637802.

Edwards, D. J., McEnteggart, C., & Barnes-Holmes, Y. (2022). A functional contextual account of background knowledge in categorization: Implications for artificial general intelligence and cognitive accounts of general knowledge. *Frontiers in Psychology*, *13*, 745306.

Edwards, D. J., McEnteggart, C., Barnes-Holmes, Y., Lowe, R., Evans, N., & Vilardaga, R. (2017). The impact of mindfulness and perspective-taking on implicit associations toward the elderly: a relational frame theory account. *Mindfulness*, *8*, 1615-1622.

Everett III, H. (1957). " Relative state" formulation of quantum mechanics. *Reviews of Modern physics*, *29*(3), 454.

Fernando, C., Banarse, D., Michalewski, H., Osindero, S., & Rocktaschel, T. (2023). *Promptbreeder: self-referential self-improvement via promt evolution*. https://arxiv.org/abs/2309.16797

Fuchs, C. A. (2010). QBism, the perimeter of quantum Bayesianism. *arXiv preprint arXiv:1003.5209*.

Fuchs, C. A. (2014). Introducing QBism. In *New directions in the philosophy of science* (pp. 385-402). Springer.

Gabriel, I. (2020). Artificial intelligence, values, and alignment. *Minds and Machines*, *30*(3), 411-437.

Gilbert, P. (2019). Explorations into the nature and function of compassion. *Current opinion in psychology*, *28*, 108-114.

Glick, D. (2021). QBism and the limits of scientific realism. *European Journal for Philosophy of Science*, *11*(2), 53.

Gray, J. A. (2004). *Consciousness: Creeping up on the hard problem*. Oxford University Press, USA.

Hayes, S. C., Barnes-Holmes, D., & Roche, B. (2001). Relational frame theory: A post-Skinnerian account of human language and cognition.

Hayes, S. C., Luoma, J. B., Bond, F. W., Masuda, A., & Lillis, J. (2006). Acceptance and commitment therapy: Model, processes and outcomes. *Behaviour Research and Therapy*, *44*(1), 1-25.

Hayes, S. C., Pistorello, J., & Levin, M. E. (2012). Acceptance and commitment therapy as a unified model of behavior change. *The Counseling Psychologist*, *40*(7), 976-1002.

Hayes, S. C., Strosahl, K. D., & Wilson, K. G. (1999). *Acceptance and commitment therapy* (Vol. 6). Guilford press New York.

Hayes, S. C., Strosahl, K. D., & Wilson, K. G. (2011). *Acceptance and commitment therapy: The process and practice of mindful change*. Guilford press.

Healey, R. (2016). Quantum-Bayesian and pragmatist views of quantum theory.

Heess, N., Hunt, J. J., Lillicrap, T. P., & Silver, D. (2015). Memory-based control with recurrent neural networks. *arXiv preprint arXiv:1512.04455*.

Hughes, S., & Barnes-Holmes, D. (2015). Relational frame theory: The basic account. *The Wiley handbook of contextual behavioral science*, 129-178.

Kahneman, D., Slovic, P., & Tversky, A. (1982). *Judgment under uncertainty: Heuristics and biases*. Cambridge university press.

Kahneman, D., & Tversky, A. (1979). Prospect Theory: An Analysis of Decision under Risk. *Econometrica*, *47*(2), 263-292.
Kahneman, D., & Tversky, A. (2013). Prospect theory: An analysis of decision under risk. In *Handbook of the fundamentals of financial decision making: Part I* (pp. 99-127). World Scientific.
Kashdan, T. B., & Rottenberg, J. (2010). Psychological flexibility as a fundamental aspect of health. *Clinical psychology review*, *30*(7), 865-878.
Keskar, N. S., McCann, B., Varshney, L. R., Xiong, C., & Socher, R. (2019). Ctrl: A conditional transformer language model for controllable generation. *arXiv preprint arXiv:1909.05858*.
Khrennikov, A. (2018). Towards better understanding QBism. *Foundations of Science*, *23*, 181-195.
Kirby, J., & Gilbert, P. (2017). The emergence of the compassion focused therapies. *Compassion: Concepts, research and applications*, 258-285.
Lewis, M., Liu, Y., Goyal, N., Ghazvininejad, M., Mohamed, A., Levy, O., Stoyanov, V., & Zettlemoyer, L. (2019). Bart: Denoising sequence-to-sequence pre-training for natural language generation, translation, and comprehension. *arXiv preprint arXiv:1910.13461*.
Lightman, H., Kosaraju, V., Burda, Y., Edwards, H., Baker, B., Lee, T., Leike, J., Schulman, J., Sutskever, I., & Cobbe, K. (2023). Let's Verify Step by Step. *arXiv preprint arXiv:2305.20050*.
Lucas, J. J., & Moore, K. A. (2020). Psychological flexibility: positive implications for mental health and life satisfaction. *Health promotion international*, *35*(2), 312-320.
Makransky, J. (2021). Compassion in Buddhist psychology.
Markov, T., Zhang, C., Agarwal, S., Nekoul, F. E., Lee, T., Adler, S., Jiang, A., & Weng, L. (2023). A holistic approach to undesired content detection in the real world. Proceedings of the AAAI Conference on Artificial Intelligence,
Melloni, L., Mudrik, L., Pitts, M., & Koch, C. (2021). Making the hard problem of consciousness easier. *Science*, *372*(6545), 911-912.
Mermin, N. D. (2014). Physics: QBism puts the scientist back into science. *Nature*, *507*(7493), 421-423.
Mermin, N. D. (2018). Making better sense of quantum mechanics. *Reports on Progress in Physics*, *82*(1), 012002.
Meta Fundamental AI Research Diplomacy Team, Bakhtin, A., Brown, N., Dinan, E., Farina, G., Flaherty, C., Fried, D., Goff, A., Gray, J., & Hu, H. (2022). Human-level play in the game of Diplomacy by combining language models with strategic reasoning. *Science*, *378*(6624), 1067-1074.
Mohrhoff, U. (2014). QBism: a critical appraisal. *arXiv preprint arXiv:1409.3312*.
Neff, K., & Tirch, D. (2013). Self-compassion and ACT. . In T. B. Kashdan & J. Ciarrochi (Eds.), *Mindfulness, acceptance, and positive psychology: The seven foundations of well-being* (pp. 78-106). New Harbinger Publications.
Ouyang, L., Wu, J., Jiang, X., Almeida, D., Wainwright, C., Mishkin, P., Zhang, C., Agarwal, S., Slama, K., & Ray, A. (2022). Training language models to follow instructions with human feedback. *Advances in neural information processing systems*, *35*, 27730-27744.
Pila, E., Gilchrist, J., Kowalski, K., & Sabiston, C. (2022). Self-compassion and body-related self-conscious emotions: Examining within-and between-person variation among adolescent girls in sport. *Psychology of Sport and Exercise*, *58*, 102083.
Pinna, T., & Edwards, D. J. (2020). A systematic review of associations between interoception, vagal tone, and emotional regulation: Potential applications for mental health, wellbeing, psychological flexibility, and chronic conditions. *Frontiers in Psychology*, *11*, 1792.
Qi, X., Zeng, Y., Xie, T., Chen, P.-Y., Jia, R., Mittal, P., & Henderson, P. (2023). Fine-tuning Aligned Language Models Compromises Safety, Even When Users Do Not Intend To! *arXiv preprint arXiv:2310.03693*.
Rovelli, C. (1996). Relational quantum mechanics. *International Journal of Theoretical Physics*, *35*, 1637-1678.

Rovelli, C. (2005). Relational quantum mechanics. In *Quo vadis quantum mechanics?* (pp. 113-120). Springer.
Russell, M., & Brickell, M. (2015). The "double-edge sword" of human empathy: A unifying neurobehavioral theory of compassion stress injury. *Social Sciences*, *4*(4), 1087-1117.
Sarfatti, J. (2004). Wheeler's world: It from bit. *Progress in Quantum Physics Research*, 41-84.
Saunders, S., Barrett, J., Kent, A., & Wallace, D. (2010). *Many worlds?: Everett, quantum theory, & reality*. OUP Oxford.
Schulman, J., Wolski, F., Dhariwal, P., Radford, A., & Klimov, O. (2017). Proximal policy optimization algorithms. *arXiv preprint arXiv:1707.06347*.
Seth, A. (2021). *Being you: A new science of consciousness*. Penguin.
Seth, A. K. (2013). Interoceptive inference, emotion, and the embodied self. *Trends in cognitive sciences*, *17*(11), 565-573.
Seth, A. K. (2014). The cybernetic Bayesian brain. In *Open mind*. Open MIND. Frankfurt am Main: MIND Group.
Seth, A. K., & Critchley, H. D. (2013). Extending predictive processing to the body: emotion as interoceptive inference. *Behavioral and brain sciences*, *36*(3), 227.
Seth, A. K., & Friston, K. J. (2016). Active interoceptive inference and the emotional brain. *Philosophical Transactions of the Royal Society B: Biological Sciences*, *371*(1708), 20160007.
Seth, A. K., Suzuki, K., & Critchley, H. D. (2012). An interoceptive predictive coding model of conscious presence. *Frontiers in Psychology*, *2*, 395.
Seth, A. K., & Tsakiris, M. (2018). Being a beast machine: The somatic basis of selfhood. *Trends in cognitive sciences*, *22*(11), 969-981.
Shear, J. (1999). *Explaining consciousness: The hard problem*. Mit Press.
Shen, X., Chen, Z., Backes, M., Shen, Y., & Zhang, Y. (2023). " Do Anything Now": Characterizing and Evaluating In-The-Wild Jailbreak Prompts on Large Language Models. *arXiv preprint arXiv:2308.03825*.
Shuster, K., Komeili, M., Adolphs, L., Roller, S., Szlam, A., & Weston, J. (2022). Language models that seek for knowledge: Modular search & generation for dialogue and prompt completion. *arXiv preprint arXiv:2203.13224*.
Silver, D., Huang, A., Maddison, C. J., Guez, A., Sifre, L., Van Den Driessche, G., Schrittwieser, J., Antonoglou, I., Panneershelvam, V., & Lanctot, M. (2016). Mastering the game of Go with deep neural networks and tree search. *Nature*, *529*(7587), 484-489.
Sims, M. (2022). Self-Concern Across Scales: A Biologically Inspired Direction for Embodied Artificial Intelligence. *Frontiers in Neurorobotics*, *16*, 857614.
Smith, E. M., Gonzalez-Rico, D., Dinan, E., & Boureau, Y.-L. (2020). Controlling style in generated dialogue. *arXiv preprint arXiv:2009.10855*.
Solms, M. (2014). A neuropsychoanalytical approach to the hard problem of consciousness. *Journal of integrative neuroscience*, *13*(02), 173-185.
Suzuki, K., Roseboom, W., Schwartzman, D. J., & Seth, A. K. (2018). Hallucination machine: Simulating altered perceptual phenomenology with a deep-dream virtual reality platform. Artificial Life Conference Proceedings,
Thompson, E. (2001). Empathy and consciousness. *Journal of Consciousness Studies*, *8*(5-6), 1-32.
Thupten, J. (2019). The question of mindfulness' connection with ethics and compassion. *Current opinion in psychology*, *28*, 71-75.
Tordjman, S., Celume, M., Denis, L., Motillon, T., & Keromnes, G. (2019). Reframing schizophrenia and autism as bodily self-consciousness disorders leading to a deficit of theory of mind and empathy with social communication impairments. *Neuroscience & Biobehavioral Reviews*, *103*, 401-413.
Torneke, N. (2010). *Learning RFT: An introduction to relational frame theory and its clinical application*. New Harbinger Publications.

Tsakiris, M., & Critchley, H. (2016). Interoception beyond homeostasis: affect, cognition and mental health. In (Vol. 371, pp. 20160002): The Royal Society.
Tversky, A., & Kahneman, D. (1974). Judgment under Uncertainty: Heuristics and Biases: Biases in judgments reveal some heuristics of thinking under uncertainty. *Science*, *185*(4157), 1124-1131.
Vaswani, A., Shazeer, N., Parmar, N., Uszkoreit, J., Jones, L., Gomez, A. N., Kaiser, Ł., & Polosukhin, I. (2017). Attention is all you need. *Advances in neural information processing systems*, *30*.
von Neumann, J. (1928). Zur theorie der gesellschaftsspiele. *Mathematische annalen*, *100*(1), 295-320.
Weidinger, L., Mellor, J., Rauh, M., Griffin, C., Uesato, J., Huang, P.-S., Cheng, M., Glaese, M., Balle, B., & Kasirzadeh, A. (2021). Ethical and social risks of harm from language models. *arXiv preprint arXiv:2112.04359*.
Wu, Z., Qiu, L., Ross, A., Akyürek, E., Chen, B., Wang, B., Kim, N., Andreas, J., & Kim, Y. (2023). Reasoning or reciting? exploring the capabilities and limitations of language models through counterfactual tasks. *arXiv preprint arXiv:2307.02477*.
